# Structure Prediction of Ionic Epitaxial Interfaces with Ogre Demonstrated for Colloidal Heterostructures of Lead Halide Perovskites

**DOI:** 10.1021/acsnano.4c12713

**Published:** 2025-02-02

**Authors:** Stefano Toso, Derek Dardzinski, Liberato Manna, Noa Marom

**Affiliations:** † Nanochemistry Department, Istituto Italiano di Tecnologia, Genova 16163, Italy; ‡ Department of Materials Science and Engineering, Carnegie Mellon University, Pittsburgh, Pennsylvania 15213, United States; § Department of Physics, Carnegie Mellon University, Pittsburgh, Pennsylvania 15213, United States; ∥ Department of Chemistry, Carnegie Mellon University, Pittsburgh, Pennsylvania 15213, United States

**Keywords:** epitaxy, interface, heterostructure, perovskite, structure prediction, DFT

## Abstract

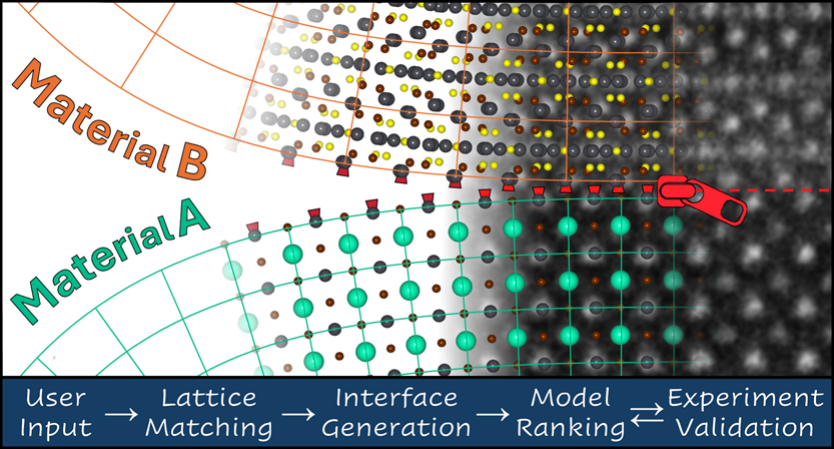

Colloidal epitaxial heterostructures are nanoparticles composed of two different materials connected at an interface, which can exhibit properties different from those of their individual components. Combining dissimilar materials offers exciting opportunities to create a wide variety of functional heterostructures. However, assessing structural compatibility—the main prerequisite for epitaxial growth—is challenging when pairing complex materials with different lattice parameters and crystal structures. This complicates both the selection of target heterostructures for synthesis and the assignment of interface models when new heterostructures are obtained. Here, we demonstrate Ogre as a powerful tool to accelerate the design and characterization of colloidal heterostructures. To this end, we implemented developments tailored for the high-efficiency prediction of epitaxial interfaces between ionic/polar materials, which encompass most colloidal semiconductors. These include the use of pre-screening candidate models based on charge balance at the interface and the use of a classical potential for fast energy evaluations, with parameters automatically calculated based on the input bulk structures. These developments are validated for perovskite-based CsPbBr_3_/Pb_4_S_3_Br_2_ heterostructures, where Ogre produces interface models in excellent agreement with density functional theory and experiments. Furthermore, we use Ogre to rationalize the templating effect of CsPbCl_3_ on the growth of lead sulfochlorides, where perovskite seeds induce the formation of Pb_4_S_3_Cl_2_ rather than Pb_3_S_2_Cl_2_ due to better epitaxial compatibility. Finally, combining Ogre simulations with experimental data enables us to unravel the structure and composition of the hitherto unsolved CsPbBr_3_/Bi_*x*_Pb_*y*_S_*z*_ interface, and to assign a structure to several other reported metal halide- and oxide-based interfaces. The Ogre package is available on GitHub or via the *OgreInterface* desktop application, available for Windows, Linux, and Mac.

## 1 Introduction

Colloidal heterostructures are nanoparticles composed of two materials connected at an interface. Such architectures can profitably combine the properties of their components, and may exhibit unique functionalities emerging from their interaction. Prominent examples are core–shell quantum dots^1−3^ and photocatalytic nanocomposites,^4−7^ whose properties stem directly from the electronic structure of the junction. The increasing availability of colloidal materials has promoted a property-driven approach to the design of new heterostructures, where materials are paired based on desired features like band alignment, plasmon resonance, or other functionalities.^8−12^ However, materials selected based on their properties are not always structurally compatible,^13^ and the absence of suitable structural relations may lead to the formation of amorphous or highly defective interfaces. Such heterostructures might still be useful if the target functionality is insensitive to the nature of the interface, as is the case for protective oxide shells.^14,15^ However, heterostructures expressing electronic, magnetic, and optical properties that arise from quantum interactions at the junction may be impaired.^16−25^

To express their full potential, such heterostructures require epitaxial interfaces that ensure a precise matching between the two materials and can grow virtually defect-free. Indeed, the most successful colloidal heterostructures are fully epitaxial architectures between isostructural materials (e.g., CdSe/CdS, CdSe/ZnSe, InP/ZnS),^2,26−29^ which combine outstanding optoelectronic properties with a simple and predictable growth process. However, isostructural interfaces cover only a small fraction of the wide design space offered by colloidal chemistry, and the increasing number of heterostructures reported between nonisostructural materials, often obtained by chance, suggests that there is much more to explore.^30−43^

Nevertheless, synthesizing new heterostructures is a painstaking process of trial and error, as even compatible materials often tend to crystallize separately, and the copresence of many elements in the reaction medium can lead to competing byproducts. Hence, failing to couple a specific pair of materials raises the question of whether such a heterostructure is intrinsically impossible to grow, or the right conditions have yet to be found. Moreover, the morphology of nanocrystals may evolve during postsynthetic treatments, complicating the identification of facets involved in the interface growth.^44−49^ Finally, the instability of some colloidal nanomaterials under an electron beam can hinder the collection of atomic resolution images, which are needed to verify if a heterostructure is truly epitaxial and determine the interface structure.^50,51^

These challenges can be addressed through computer simulations, which help direct synthetic efforts toward materials that are likely to match epitaxially and can provide models to assist the interpretation of experiments. Several tools for the prediction of interface structures have been developed to this end.^48,52−59^ These often employ hierarchical workflows, in which fast methods like classical force fields, score functions, or machine learning are used for the initial screening of models, followed by density functional theory (DFT) calculations to predict the structure and properties of the most promising candidates.^58−65^ A similar approach is implemented in the Ogre open-source Python package for the prediction of organic and inorganic epitaxial interfaces.^66−69^ Like other tools of its kind, Ogre was initially developed with the goal of predicting the structure of large-area epitaxial interfaces grown by thin film deposition methods, and its first applications focused on semiconductor/metal interfaces.^66^ Such tools are not well-suited to identify prospective interfaces that could be grown by colloidal chemistry, as this requires a quick evaluation of numerous interfaces rather than the detailed simulation of a few. The task is further complicated by the complex compositions and structures adopted by many colloidal materials, like the lead halide perovskites studied here, which make approaches that rely heavily on DFT less suitable. Lastly, most structure prediction codes are designed for specialists, while synthetic chemists would benefit from tools that are easy to use, and do not require coding experience or access to advanced computing facilities.

To address these needs, we have implemented in Ogre a fast workflow for the prediction of epitaxial interfaces between polar compounds, which encompass most colloidal nanomaterials (e.g., CsPbBr_3_, CdS, ZnO, etc.). This includes two new features that take advantage of the predominantly ionic nature of these materials to dramatically accelerate predictions. The first is a preliminary screening of interface models based on charge balance, which reduces the computational load by eliminating unreasonable candidates. The second feature is a classical potential consisting of a Coulomb term^70,71^ and a repulsive Born term,^66,72^ whose parameters are determined automatically based on the bulk structures of the input materials. This potential allows to quickly evaluate the energy of candidate structures, and can produce interface models and energy rankings in good agreement with DFT at a fraction of its computational cost. These strategies enable predicting the structure of epitaxial interfaces between polar materials in just few minutes on a simple laptop, making Ogre an excellent screening tool. We note that this approach is not suitable when the dominant interactions are not electrostatic, as is the case for metallic, covalent, and van der Waals dispersion bonding. To cover these cases, DFT, machine-learned potentials, and alternative score functions are available in Ogre.^66−69^

In what follows, the new Ogre workflow for the prediction of polar epitaxial interfaces is presented and validated extensively for several case studies. In Section 2.1, we illustrate the full workflow in detail, using the CsPbBr_3_/Pb_4_S_3_Br_2_ colloidal heterostructures reported by some of us as an example.^73,74^ For this system, Ogre correctly identifies all the known epitaxial relations, and for the case study of the (100)//(010) orientation it produces an interface model that matches both DFT-based predictions and high-resolution scanning transmission electron microscopy (HR-STEM) images of the heterostructures. In Section 2.2, we demonstrate how Ogre can help elucidate the outcome of a synthesis by revisiting a previous study of perovskite/lead sulfochloride heterostructures, conducted by some of us.^75^ Specifically, the empirical observation that CsPbCl_3_ nanocrystals template the selective nucleation of Pb_4_S_3_Cl_2_ while suppressing the growth of the competing phase Pb_3_S_2_Cl_2_ is rationalized based on Ogre’s predictions of lattice matching and interface stability. In Section 2.3, we revisit several heterostructures involving CsPbBr_3_ to demonstrate how Ogre can help assign structural models to newly synthesized interfaces, taking advantage of characterization results as a part of the prediction workflow.^34,36,76,77^ A prominent example is the CsPbBr_3_/Pb–Bi–S heterostructures recently reported by some of us,^39^ whose hitherto unknown composition and interface connectivity are unraveled by combining information from HR-TEM experiments with Ogre predictions. Finally, in Section 2.4 we demonstrate Ogre’s broad applicability beyond colloidal metal halides by reproducing the structure of known heteroepitaxial interfaces between pairs of oxides, which are among the most studied polar materials for thin film growth.^78−81^

In addition to being efficient and versatile, Ogre is accessible to nonspecialist users via the *OgreInterface* desktop application, available for Windows, Linux, and Mac. All scripts and reference bulk structures needed to reproduce the results presented here are available as Supporting Information, and serve as examples for beginner users. By being directly accessible to synthetic chemists, we expect Ogre to become a versatile and powerful tool for the discovery and investigation of new epitaxial heterostructures between polar materials.

## 2 Results and Discussion

### 2.1 Ogre Workflow Demonstrated for CsPbBr_3_/Pb_4_S_3_Br_2_

**Figure 1:**
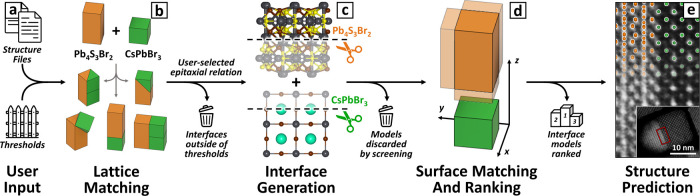
Ogre interface prediction workflow. (a) Ogre takes as input the bulk structures of two materials, along with user-defined thresholds for strain, supercell area, and the Miller indices of lattice planes to consider. (b) The *latticematching* step identifies all the commensurate supercells that yield domain-matched interfaces within the specified thresholds. (c) For selected epitaxial orientations, the *interface generation* step creates all possible surface terminations for both materials, and combines them to construct atomistic models of the interface. (d) For each model, the *surface matching and ranking* step identifies the optimal epitaxial registry (i.e., lateral offset) and distance between the two domains, using a fast electrostatic potential to evaluate energies. (e) The output is a set of atomistic models ranked by stability. Top-ranked models can be compared with available experimental data, as demonstrated here for a Pb_4_S_3_Br_2_/CsPbBr_3_ heterostructure (only heavy atoms shown for clarity). HR-STEM images adapted with permission.^73^ Copyright 2023, American Chemical Society.

Given the crystal structure of two materials, *A* and *B*, Ogre first identifies all favorable commensurate epitaxial relations between them, and then attempts to predict the structure of the resulting interfaces. The output is a set of atomistic models with optimized epitaxial registry (i.e., lateral offset) and interface distance, ranked by energy from the most to the least stable. The prediction workflow proceeds through three steps, illustrated in Figure 1: *lattice matching*, *interface generation*, and *surface matching and ranking*. Sections 2.1.1–2.1.3 demonstrate each step of the algorithm for the (100)//(010)–CsPbBr_3_/Pb_4_S_3_Br_2_ epitaxial interface, which some of us have previously characterized in the form of colloidal heterostructures.^73^ In Section 2.1.4, the performance of the Ogre classical potential is validated against DFT for the same interface.

We note that in this work CsPbBr_3_ is treated as cubic despite adopting a slightly distorted orthorhombic structure.^82−85^ This approximation greatly simplifies the discussion of results, and does not adversely affect the outcome of predictions (see Figures S1–S7 and Tables S1–S4).

#### 2.1.1 Lattice Matching

The *lattice matching* step uses the Zur-McGill algorithm^52^ to identify commensurate epitaxial relations between the two materials, denoted here as (*hkl*)_*A*_//(*h′k*′*l*′**)_*B*_ based on the lattice planes being matched. In short, the algorithm attempts to combine multiple 2D-cells describing the lateral periodicity of (*hkl*)_*A*_ and (*h*′*k*′*l*′**)_*B*_ planes to construct a common 2D-supercell that can represent both materials at the interface. An epitaxial match is found if such supercell(s) exist within the user-defined constraints for strain and area. We note that one (*hkl*)_*A*_//(*h*′*k*′*l*′**)_*B*_ combination may produce several nonequivalent 2D-supercells that differ by a relative rotation of the two materials around an axis perpendicular to their contact plane.^66^ In such cases, the smallest 2D-supercell is selected (see Figure S8 and related discussion). To avoid any ambiguities, Table S5 specifies for all interfaces discussed in this work a pair of lattice vectors that are parallel to each other in the plane of the interface, denoted here as [*hkl*]_A_
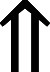
[*h*′*k*′*l*′**]_B_.

At this stage, the user can impose constraints on strain and supercell area to exclude implausible matches. In general, high strain is unfavorable, as it can lead to the formation of defects at the interface or even hinder its growth. However, a certain degree of mismatch is tolerable, depending on the type of interface being studied. For example, the strain limit for large-area, high-quality epitaxial semiconductor films is considered to be around 2%.^86^ Conversely, nanoscale interfaces tend to be more tolerant because strain can be effectively absorbed by lattice deformations if the contact surface is limited to few nm^2^. For instance, the CsPbBr_3_/CsPb_2_Br_5_ heterostructures reported by Zheng et al. accommodate a 2.7% mismatch by bending into rings where the material with the largest lattice step faces outward (see Section 2.2).^36^ Other examples of high-strain colloidal interfaces are CdS/CdSe (4.2%)^87^ and the rather extreme InAs/ZnS (12.0%).^88^ For the colloidal heterostructures studied here, we set the strain threshold to 10%.

For selecting the area threshold there are conflicting considerations. On the one hand, larger supercells enable finding commensurate domains with lower strain, approaching the limit where an infinite area produces supercells without any strain. On the other hand, smaller supercells increase the chance of good atom-to-atom correspondence at the interface because the lattice sites of the two materials are more likely to coincide. By default, Ogre sets the surface area threshold, *S*_T_, to
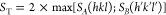
1

In short, for each (*hkl*)_*A*_//(*h*′*k*′*l*′**)_*B*_ pair, *S*_T_ is set to twice the area of the largest 2D-cell among the two planes. This enables finding supercells that can encompass up to two single-material cells positioned side-by-side, thus allowing for a more extended interface repeating unit. The user can select a hard numerical threshold if needed (see eq S7 and related discussion).

The user may also impose constraints on the Miller indices to be searched, based on their pre-existing knowledge of the system of interest. For example, the planes exposed by one or both materials might be known, as is the case for nanocrystal seeds with well-defined facets, or for the epitaxial growth of a thin film on a single-crystal substrate. In the absence of such information, or if multiple hypothetical orientations are being considered, it is still reasonable to limit the search to low Miller indices because high-index crystal terminations are usually less stable, and therefore rarely exposed.^44−47,49,89^

**Figure 2:**
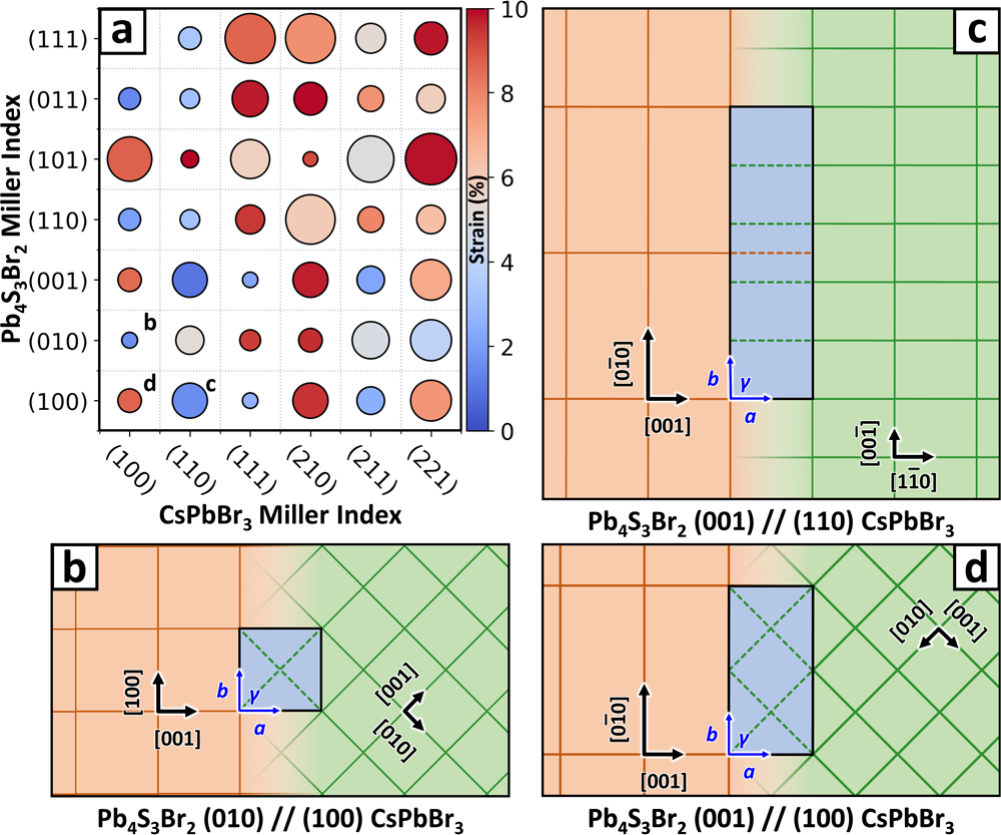
*Lattice matching*results for CsPbBr_3_/Pb_4_S_3_Br_2_. (a) Domain-matched interfaces identified within the specified constraints. Each circle indicates a match, with the color corresponding to the strain and the size representing the interface area. Interfaces marked with letters have been reported experimentally, and their 2D-supercell is shown in the corresponding panels. (b–d) Supercells of the CsPbBr_3_/Pb_4_S_3_Br_2_ interfaces reported in (b) ref (73) and (c,d) ref (90). The CsPbBr_3_ 2D-lattice is colored in green, the Pb_4_S_3_Br_2_ 2D-lattice is colored in orange, and the domain-matched commensurate 2D-supercell is shown in blue.

Figure 2 summarizes the lattice matching results for the CsPbBr_3_/Pb_4_S_3_Br_2_ system. For ease of visualization *h,k,l* ≤ 1 for Pb_4_S_3_Br_2_ and *h,k,l* ≤ 2 for CsPbBr_3_ are shown here (see Figure S9 for a more extended search). Each circle in Figure 2a corresponds to a domain-matched epitaxial relation identified within the constraints: the color indicates the strain, while the size represents the supercell area. Interfaces reported experimentally are marked with a letter, indicating the corresponding 2D-supercell (Figure 2b–d).^73,90^ The (100)//(010)–CsPbBr_3_/Pb_4_S_3_Br_2_ interface (Figure 2b) stands out for having both a low strain of 1.6% and a small area of 68 Å^2^. Indeed, this interface was the first to be reported and fully characterized by some of us for the CsPbBr_3_/Pb_4_S_3_Br_2_ system.^73^

Ogre also identifies two additional epitaxial relations that were recently reported by Das et al:^90^ the (110)//(001) interface, with 0.9% mismatch and an area of 241 Å^2^ (Figure 2c), and the (100)//(001) interface, with 8.6% mismatch and an area of 136 Å^2^ (Figure 2d). Because these interfaces were observed in nanoscale heterostructures, the contact surface between the two materials was likely small enough to tolerate what would otherwise be a high strain value. It is worth noting that the *a* and *c* lattice parameters of Pb_4_S_3_Br_2_ are very close, leading to (*hkl*) ≈ (*lkh*) for this material. This causes similarities between the matches found by Ogre for the (100) and (001) planes of Pb_4_S_3_Br_2_, and would also complicate the experimental distinction between these orientations. In the absence of atomic-resolution images of the interface, we cannot exclude that some of the heterostructures observed by Das et al.^90^ might have adopted alternative epitaxial relations (see Figures S10–S14 and Tables S6–S10 for further discussion).

Based on the results shown in Figure 2, there are several additional plausible matches that have not been reported experimentally. For example, the (111)//(100) interface (2.5% strain and 118 Å^2^ area) and the (110)//(110) interface (3.1% strain and 145 Å^2^ area) appear comparable to the (100)//(010) interface reported by some of us, and more favorable than those reported by Das et al.^90^ It is likely that these interfaces have not been observed owing to the known tendency of CsPbBr_3_ nanocrystals to express the (100) facets.^44,91^

#### 2.1.2 Interface Generation

The *lattice matching* procedure relies solely on lattice parameters, with no consideration of interatomic interactions and bonding at the interface. To assess if any of the identified epitaxial relations can produce a chemically stable interface, an exhaustive set of atomistic models must be constructed and ranked by relative energy. To this end, Ogre cleaves the two materials at planes parallel to the interface to generate surface slabs with different terminations,^66,92^ which are then combined to construct interface models. At this stage, Ogre can be set to distribute the strain on the two materials equally, as is the case in most colloidal heterostructures, or instead let the lattice of one material conform to the other, which might be better suited to describe the growth of a thin film on a bulk substrate.

**Figure 3:**
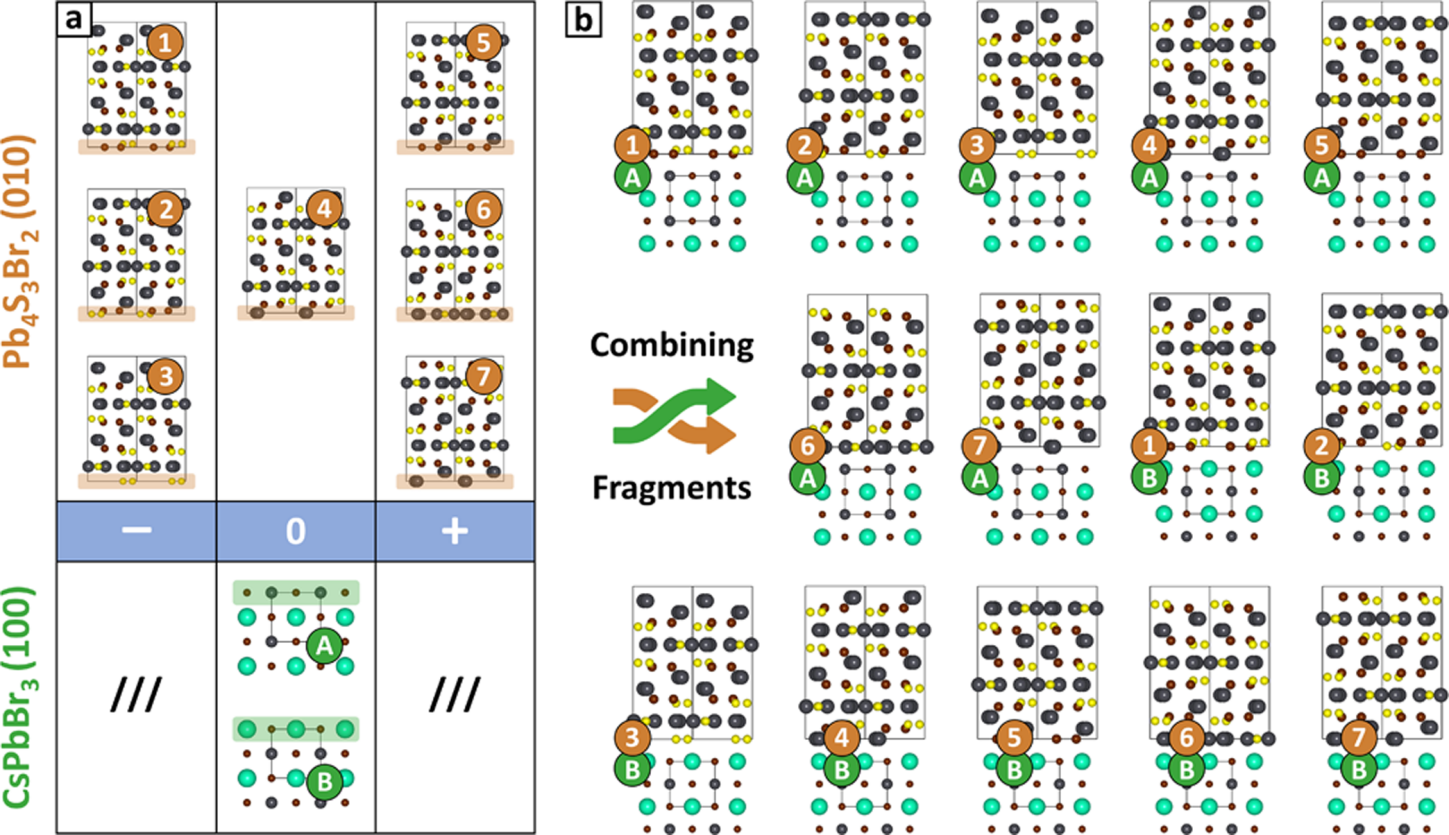
Interface generation. (a) Possible terminations for the (010) Pb_4_S_3_Br_2_ and (100) CsPbBr_3_ surfaces. +, 0, and – symbols refer to the surface charge of each slab. Colored shadings indicate the side of the slab that will contact the other material to form the interface, which is where the charge is calculated. (b) Models of all the (100)//(010)–CsPbBr_3_/Pb_4_S_3_Br_2_ nonequivalent interfaces obtained by combining the slabs in panel (a), shown prior to the *surface matching* step. Cs atoms are colored in cyan, Pb in gray, S in yellow, and Br in brown.

Figure 3 illustrates the construction of interface models for the (100)//(010)–CsPbBr_3_/Pb_4_S_3_Br_2_ interface.^73^ Depending on the complexity of the materials involved, this process can result in a large number of candidate structures. Therefore, multiple strategies are implemented to minimize the number of models that proceed to the *surface matching and ranking* step of the algorithm, which is the most computationally intensive. First, when generating the slab models, Ogre clusters together nearly coplanar atoms to construct only chemically sound surface terminations. The resulting slabs are then screened to eliminate those that are equivalent by symmetry, thus avoiding redundancies (see Figures S15–S16).

To further reduce the number of interface models, we have developed a fast screening algorithm based on the charge balance at the interface. To this end, each slab is assigned a surface charge *Q*, defined as
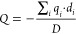
2where *i* runs over all ions in the slab, *q*_*i*_ is the ion charge, *d*_*i*_ is its distance from the interface, and *D* is the total thickness of the slab (see eqs S8 and S9 and Figure S17 for derivation). Conceptually, *Q* represents the uncompensated charge found at the surface of each material, which will interact with the other material to construct the interface. Because charges of the same sign repel each other, interfaces formed by [+/+] or [−/−] slab pairs can be immediately excluded based on the sign of *Q*.

In principle, the magnitude of *Q* could also be used to identify [+/–], [+/0], and [−/0] models that do not achieve full charge balance at the interface, which could serve as a further screening criterion. However, plenty of examples for unbalanced and yet stable interfaces have been reported, such as Sm_2_CuO_4_/LaFeO_3_,^93^ LaAlO_3_/SrTiO_3_,^94^ and many Si/oxide^95^ or metal/oxide^96^ interfaces. This is possible because charge imbalance can be locally compensated by the formation of defects (ionic compensation) or a change of oxidation states (electronic compensation), which are often considered as the origin of 2D-conductivity in epitaxial interfaces between insulators.^94,97,98^

The effectiveness of such compensation mechanisms depends on the materials involved, as not all elements can assume multiple oxidation states, and different structures might tolerate defects to a lesser extent than others. Furthermore, considering charge compensation mechanisms requires simulations that are too elaborate to be performed at a preliminary screening stage. For example, achieving a realistic concentration of defects may require large supercells, and multiple configurations must be considered to account for their random distribution. Conversely, variable oxidation states can be implicitly handled by DFT without affecting the size of the simulation, but pose challenges for classical force fields that explicitly assign a charge to atoms. Nevertheless, Section 2.1.3 shows that the classical potential used for *surface matching and ranking* can handle unbalanced interfaces even in the absence of charge compensation mechanisms. Therefore, we opted to retain the [+/–], [+/0], and [−/0] cases for further scrutiny. The user can decide to exclude such interface models based on their knowledge of the system of interest.

Overall, the effectiveness of the preliminary screening strategies employed by Ogre depends on the materials being studied. For example, Figure 3 shows all the possible slab combinations considered further for the (100)//(010)–CsPbBr_3_/Pb_4_S_3_Br_2_ interface. In this case, the number of Pb_4_S_3_Br_2_ slabs is decreased from 14 to the 7 shown in Figure 3a by excluding symmetry-equivalent terminations, whereas the only two possible terminations for cubic CsPbBr_3_ (i.e., CsBr and PbBr_2_ planes formed by coplanar ions) cannot be reduced further. The advantage of clustering becomes more evident when considering the orthorhombic structure of CsPbBr_3_, where ions are slightly shifted out of planarity. In that case, some of the possible cleavage planes would separate those ions, producing defective slabs with missing atoms at their surface. By clustering nearly coplanar ions together, Ogre identifies only the two chemically sound terminations also found for cubic CsPbBr_3_ (see Figure S16). Moreover, as both CsPbBr_3_ terminations yield slabs of the *Q* = 0 type, none of the 14 resulting interface models in Figure 3b are excluded based on charge balance. The CsPbBr_3_/Bi_2_PbS_4_ interface discussed in Section 2.3 provides an example of effective screening based on charge balance, where the number of models is reduced from 20 to 12 by excluding the [+/+] and [−/−] cases (see Figures S17 and S18).

#### 2.1.3 Surface Matching and Ranking

**Figure 4:**
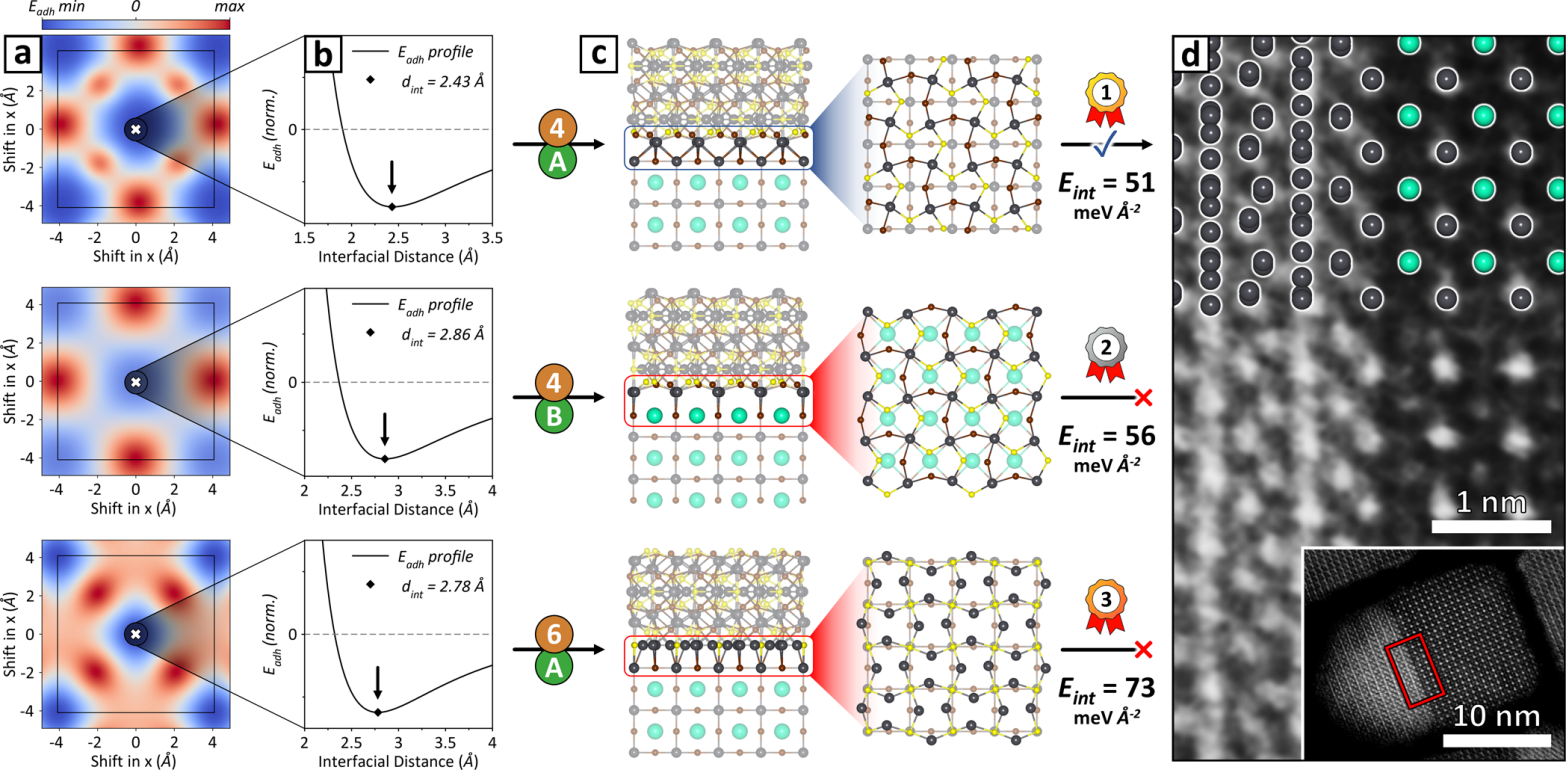
Surface matching and ranking. Results are displayed for the 4A, 4B, and 6A models of the (100)//(010)–CsPbBr_3_/Pb_4_S_3_Br_2_ interface, labeled as shown in Figure 3. (a) Maps of *E*_adh_ vs *xy*-offset with the optimal epitaxial registry marked by a cross. (b) *E*_adh_ vs interfacial distance curves, with the optimal *z*-distance indicated by an arrow. (c) Interface models after the *surface matching* step. Side views (left) highlight the formation of bonds between slabs. Top-down views (right) of the interface planes show the atom-to-atom correspondence of anions and cations. Interface energies of the three models are also shown. (d) HR-STEM image of a (100)//(010)–CsPbBr_3_/Pb_4_S_3_Br_2_ heterostructure, with the most stable model predicted by Ogre superimposed (see also Figure S4). Only heavy atoms are shown to ease the comparison with electron scattering contrast. Inset: lower magnification view of the same heterostructure. Microscopy data adapted with permission.^73^ Copyright 2023, American Chemical Society. Cs atoms are colored in cyan, Pb in gray, S in yellow, and Br in brown.

When pairing two surface slabs, their relative position must be optimized to identify the best possible bonding configuration across the interface. To this end, Ogre employs particle swarm optimization^99,100^ to efficiently explore the 3D space of parameters formed by the relative epitaxial registry (i.e., lateral *xy*-offset) and *z*-distance between the two slabs. The optimal configuration is the one that minimizes the energy of the interface model, *E*_*AB*_*.* To visualize the outcome of *surface matching*, Ogre produces a 2D energy map by shifting one material on top of the other within the interface supercell at their optimal *z*-distance (Figure 4a), and computes an energy vs interfacial distance curve at their optimal *xy*-offset (Figure 4b). For visualization purposes, these plots display the adhesion energy *E*_adh_, which is obtained by subtracting from *E*_*AB*_ the energies of the two isolated slabs (*E*_*A*_ and *E*_*B*_). The sign of *E*_adh_ indicates whether or not the resulting interface model is more stable than the two constituent surface slabs. In these maps, the global minimum of *E*_adh_ corresponds to the relative slabs position that produces the most stable configuration, while local minima identify metastable configurations. As a part of its output, Ogre produces an atomistic model of the optimal interface configuration (Figure 4c), which can be opened with structure visualization programs like Vesta.^101^

Next, all surface-matched interface models are compared to identify the most stable interface structure. As a ranking metric, Ogre adopts the interface energy *E*_int_, defined as^68^
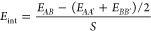
3where *E*_*AA*′**_ and *E*_*BB′*_ are the cleavage energies of the two materials *A* and *B* (i.e., the energy obtained when two slabs are reassembled into a bulk-like slab with twice the thickness at a given cleavage plane), and *S* is the supercell area. Essentially, *E*_int_ compares the energy of the interface against that of the parent bulk materials, indicating how favorable it is to interrupt the growth of one domain and switch to the other (See Figures S19–S22 for further discussion).

As mentioned above, evaluating the energy terms (*E*_*AB*_, *E*_*AA*′**_, etc.) is the most computationally intensive task of the algorithm. To make this step computationally efficient, we have developed a classical potential that enables estimating the energy of interfaces at a fraction of the computational cost of DFT. In short, all energy terms are calculated using pairwise interatomic potentials, consisting of an electrostatic Coulomb term computed via the damped-shifted force potential,^70,71,102^ and a Born term accounting for the short-range interatomic repulsion (see eqs S10–S16).^66,72^ A cutoff of 18 Å is adopted because longer-range dispersion interactions are typically negligible in ionic materials. To enable our classical potential to describe a wide variety of polar materials, each pairwise term is optimized for the corresponding pair of ions by fitting one free parameter in the Born term. This is performed by demanding that the energy minimum coincides with the equilibrium bond length found in the input bulk structures (see eq S13). For bonds that only exist at the interface (e.g., Cs^+^–S^2–^ in CsPbBr_3_/Pb_4_S_3_Br_2_), the optimal equilibrium distance is estimated based on the sum of ionic radii extracted from the two parent materials (see Figure S23). Because the fitting procedure relies on the input bulk structures as a reference, we find that the predicted interfacial distances are slightly more accurate when bonds that form between slabs also exist in the bulk materials. However, stable models typically converge to reasonable distances in either case.

We note that there are occasional instances of “*nonbonding*” interfaces, for which the energy vs interfacial distance curve is purely repulsive, and no minimum is found within the range considered (see Figure S22). This likely happens because our classical potential does not contain a long-range dispersion term. Because this only occurs when the electrostatic interactions between the two materials are not particularly favorable and the repulsive Born term dominates, we regard such interface configurations as not viable and discard them.

Due to its purely electrostatic nature, our potential is restricted to compounds in which atoms carry a formal charge. This encompasses the majority of materials found in heterostructures synthesized colloidally with the notable exception of metal domains, which are relevant for plasmonic and catalytic applications.^7,9^ However, metals often grow independently of epitaxial constraints due to their high tolerance for defects, to the point that nonsubstrate-specific growth strategies are available for technologically relevant elements (e.g., Au, Ag).^103,104^ Likewise, our potential is not applicable to covalent and van der Waals materials, where non-electrostatic interactions are predominant. We remark that Ogre can predict the structure of interfaces involving nonpolar materials by using other methods for *surface matching and ranking*, including geometric score functions, machine-learned potentials, and DFT, albeit the latter has a significantly higher computational cost.^66−69^

Figure 4 shows the *surface matching and ranking* results for the three (100)//(010)–CsPbBr_3_/Pb_4_S_3_Br_2_ interface models ranked as most stable by our classical potential: Panels 4a,b illustrate the search for the optimal epitaxial registry and interface distance, while Panel 4c shows the resulting structures (see Figures S3 and S4 and Table S2 for a full account of the results). In each case, the procedure identifies the optimal bonding configuration, with the two slabs positioned within plausible distances of each other, and according to an epitaxial registry that maximizes the attractive interactions. Notably, the two most stable interfaces, labeled 4A and 4B as in Figure 3, are of the [0/0] type and are very close in energy (52 vs 57 meV Å^–2^). In contrast, the 6A model corresponds to a charge-unbalanced [+2/0] interface, and is significantly less stable (75 meV Å^–2^). This suggests that a charge accumulation can destabilize the interface despite the good geometric matching between slabs and the presence of favorable Pb^2+^–S^2–^ electrostatic interactions, that are absent in the charge-balanced models 4A and 4B. We note, however, that the classical potential is unable to redistribute charges at the interface, as discussed in Section 2.1.4.

As shown in Figure 4d, the top-ranked model 4A matches well with experimental observations of the interface.^73^ The model produced by Ogre was scaled to match the atomic-resolution image of the heterostructure without any adjustment to the relative position of the two domains, which attests to the accuracy of our prediction (see Figure S4 for a comparison with other models). To provide a practical estimate of the code’s performance, the whole workflow illustrated in Section 2.1 was executed on a mid-tier laptop in about ∼100 s.

#### 2.1.4 Validation Against DFT

To further validate the performance of our classical potential, we compared the *surface matching and ranking* results for the (100)//(010)–CsPbBr_3_/Pb_4_S_3_Br_2_ interface to DFT. Because the choice of exchange correlation functional can significantly affect the outcome of DFT simulations, it is important to select one that adequately describes the materials of interest. Here, we have chosen the strongly constrained and appropriately normed (SCAN) functional^105^ combined with the revised Vydrov and van Voorhis nonlocal correlation method (rVV10)^106,107^ because it has been reported to reliably describe the dynamic tilting of octahedra and the phase transitions in halide perovskites.^108,109^ In order to compare the results of the Ogre classical potential to DFT on an equal footing, the same workflow of *surface matching and ranking* was executed using DFT, with no structural relaxation beyond optimizing the relative position of the two rigid slabs.

**Figure 5:**
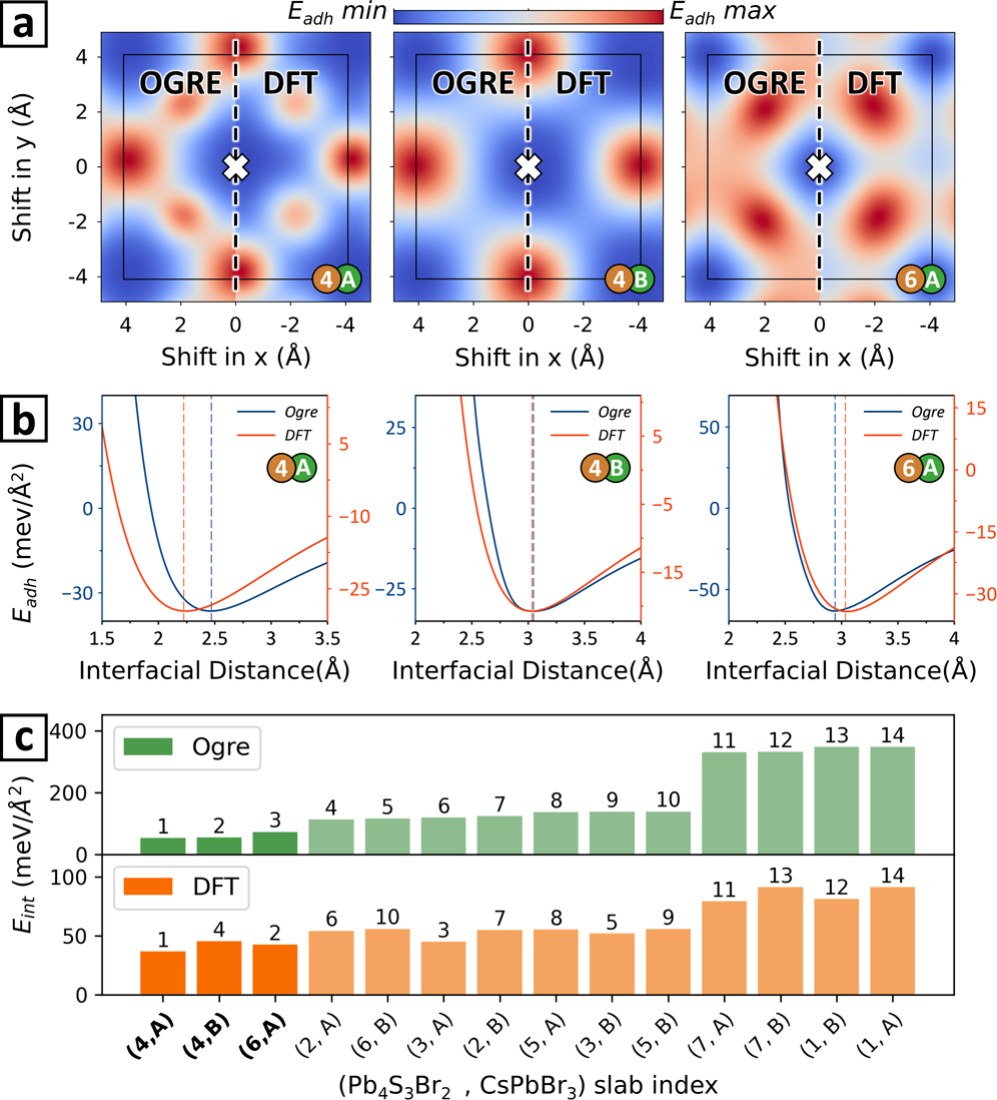
Validation of surface matching and ranking by the Ogre potential against DFT. (a) *E*_adh_ maps generated with the Ogre classical potential (left) and DFT (right) for the 4A, 4B, and 6A models of the (100)//(010)–CsPbBr_3_/Pb_4_S_3_Br_2_ interface (labeled as in Figure 4). Both methods identify the same epitaxial registry, marked by a cross. (b) *E*_adh_ vs interfacial distance curves for the same interfaces. (c) Interface models ranked by computing *E*_int_ with the Ogre electrostatic potential (top) and with DFT (bottom) with the 4A, 4B, and 6A models highlighted.

For the 4A, 4B, and 6A interface models shown in Figure 4, we compared the 2D energy maps (Figure 5a) and the *E*_adh_ vs interfacial distance curves (Figure 5b) produced by the Ogre classical potential and by DFT. In all cases, our classical potential is in good agreement with DFT regarding the positions of the energy maxima and minima, meaning that the optimal in-plane epitaxial registry is correctly identified. The interfacial distance is in closer agreement with DFT for configurations 4B and 6A than for 4A, but it is consistently within 0.3 Å from the DFT results. Overall, our classical potential tends to overestimate the interfacial distances compared to DFT, with increasing inaccuracy as the interface models become less stable. This is likely because the classical potential does not include nonionic contributions to the binding energy, and does not allow for charge density redistribution (see Table S11 for further discussion).

Figure 5c compares the ranking of interface models based on the Ogre classical potential and DFT. Both methods rank the experimental interface 4A as the most stable, and highlight a clear distinction between two groups of interfaces with lower and higher interface energies. Within each group the interface energies are similar, leading to some reshuffling in the two rankings. Notably, the high-energy group is formed by models with strong charge imbalance at the interface (7A and 7B = [+4,0]; 1A and 1B = [−4,0]). Conversely, in the lower-energy group the Ogre classical potential favors the two charge-balanced interfaces 4A and 4B, whereas DFT ranks two charge-imbalanced interfaces as second and third (6A = [+2,0] and 3A = [−2/0], respectively). As mentioned above, both the 6A and 3A models feature favorable Pb^2+^–S^2–^ electrostatic interactions. It is possible that with DFT these interfaces are stabilized by charge density redistribution. We also note that local geometry relaxation at the interface may further change the stability ranking. In this respect, the Ogre potential can be used to quickly generate reasonable starting models for DFT-based relaxations.

### 2.2 Using Ogre to Support Synthetic Efforts

In what follows, we demonstrate potential applications of Ogre in the field of colloidal nanomaterials for the interpretation and prediction of possible outcomes of the synthesis of epitaxial heterostructures. Because synthesizing new materials is beyond the scope of this work, we use the results for lead sulfochlorides reported by some of us as a case study.^75^

Under typical colloidal synthesis conditions, the Pb–S–Cl system can lead to the growth of two competing materials: the pseudocubic Pb_3_S_2_Cl_2_ (a phase with no known equivalent for other halogens) and the orthorhombic Pb_4_S_3_Cl_2_, which is structurally equivalent to the Pb_4_S_3_Br_2_ sulfobromide discussed in Section 2.1.^74,75^ Indeed, the presence of CsPbCl_3_ nanocrystals in the reaction medium leads to the growth of CsPbCl_3_/Pb_4_S_3_Cl_2_ heterostructures similar to those in Figure 4d. Crucially, their formation coincides with a complete suppression of the pseudocubic competitor Pb_3_S_2_Cl_2_. This outcome has been attributed to the growth of Pb_4_S_3_Cl_2_ outpacing Pb_3_S_2_Cl_2_ thanks to the epitaxial templating action of CsPbCl_3_ seeds, which enables heterogeneous nucleation.^75^ This interpretation relies on the assumption that no competing interface can form between the pseudocubic Pb_3_S_2_Cl_2_ and CsPbCl_3_, which we confirm here using Ogre. We note that the concept of promoting the growth of metastable phases by epitaxial templating is widespread in thin film growth,^110−113^ but is nascent in colloidal synthesis.

For Pb_4_S_3_Cl_2_ no bulk crystal structure was available, and the nanocrystals obtained in ref (75) were too small to allow for a proper refinement by X-ray diffraction. Therefore, we extracted the lattice parameters directly from the HR-STEM images of a CsPbCl_3_/Pb_4_S_3_Cl_2_ heterostructure via the Fourier transform analysis of lattice periodicities. To ensure maximum accuracy, we calibrated the image using the lattice constant of CsPbCl_3_ (5.605 Å) as a reference. This resulted in a pseudotetragonal unit cell for Pb_4_S_3_Cl_2_, with estimated lattice parameters *a* = *c* = 7.94 Å and *b* = 14.94 Å. The atom positions inside the unit cell were determined by starting from the analogous structure of Pb_4_S_3_Br_2_ and performing DFT relaxation, using the SCAN+rVV10 functional with the lattice parameters fixed at the estimated values (see Figure S24). Because the CsPbCl_3_ nanocubes acting as seeds expose the (100) facets, we focused on interfaces of the (100)//(*hkl*) type with *h,k,l* ≤ 2 for both Pb_4_S_3_Cl_2_ and Pb_3_S_2_Cl_2_ (see Figure S25).

**Figure 6:**
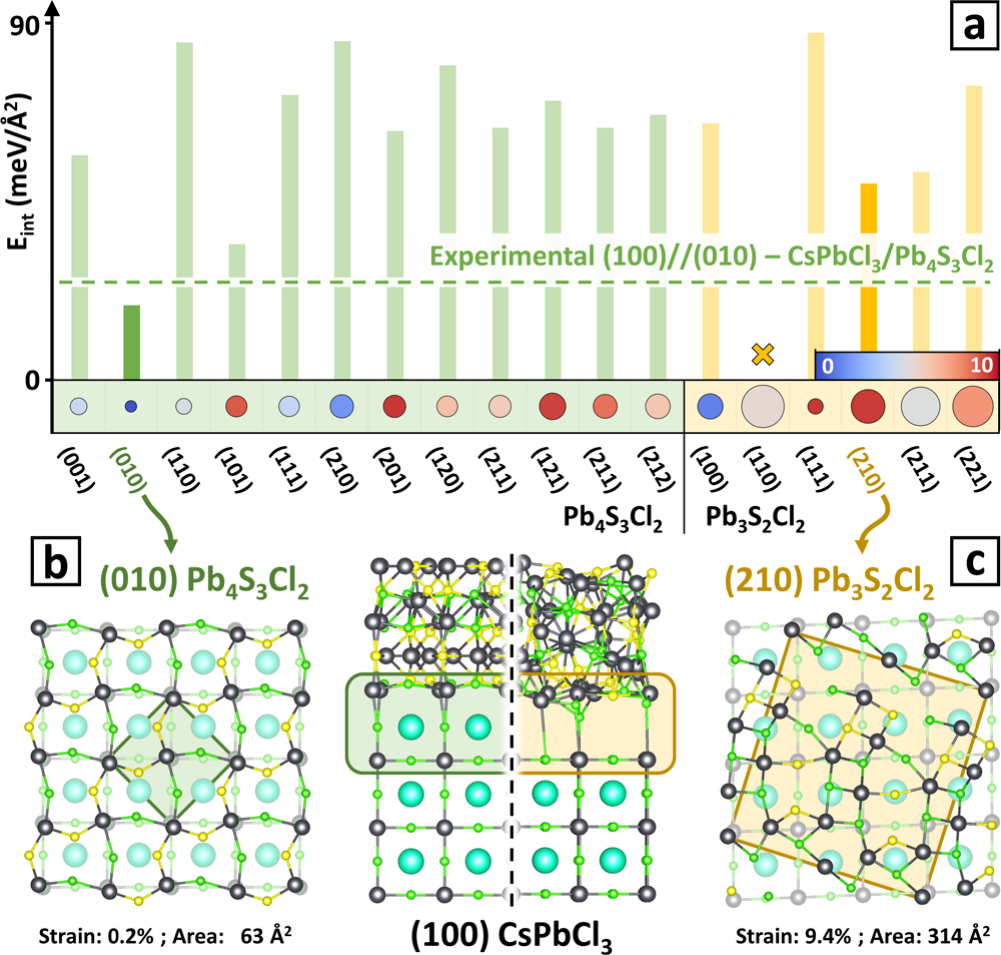
Stability of Pb_4_S_3_Cl_2_ vs Pb_3_S_2_Cl_2_ on the (100) surface of CsPbCl_3_. (a) *Lattice matching* and interface ranking results for the growth of Pb_4_S_3_Cl_2_ (green) and Pb_3_S_2_Cl_2_ (yellow) on the (100) surface of CsPbCl_3_. The circle size corresponds to the interface area and the color corresponds to the strain. The green dashed line marks the *E*_int_ value of the experimental interface, which is the second-best model for the (100)//(010)–CsPbCl_3_/Pb_4_S_3_Cl_2_ interface. Nonequivalent epitaxial relations leading to identical *lattice matching* results, like (*h,k,l*) = (*h̅*, *k̅*, *l̅*) for Pb_3_S_2_Cl_2_, have been aggregated for visualization purposes, and only the lowest *E*_int_ value is shown. The (100)//(110)–CsPbCl_3_/Pb_3_S_2_Cl_2_ interface marked with an × is found to produce only nonbonding models. (b) Model of the most stable interface formed between the (100) surface of CsPbCl_3_ and Pb_4_S_3_Cl_2_ (left: interface plane, center: side view). (c) Model of the most stable interface formed between the (100) surface of CsPbCl_3_ and Pb_3_S_2_Cl_2_ (center: side view, right: interface plane). The corresponding 2D-supercells are also shown. Cs atoms are colored in cyan, Pb in gray, S in yellow, and Cl in green.

Figure 6 shows a comparison of Ogre’s results for interfaces of CsPbCl_3_(100) with Pb_4_S_3_Cl_2_ and Pb_3_S_2_Cl_2_ (see Table S12 for additional information). In the *lattice matching* step, all orientations considered for both Pb_4_S_3_Cl_2_ and Pb_3_S_2_Cl_2_ produce supercells within the specified thresholds for strain and area. Of these, the experimentally observed (100)//(010)–CsPbCl_3_/Pb_4_S_3_Cl_2_ epitaxial relation already stands out as the most promising because of its lowest strain of 0.2% and smallest area of 63 Å^2^. The results of *surface matching and ranking* confirm that the (100)//(010)–CsPbCl_3_/Pb_4_S_3_Cl_2_ orientation is indeed the most stable. The interface structure shown in Figure 6b is well-connected, which correlates with its high stability. We note that the lowest *E*_int_ of 19 meV Å^–2^ is obtained for the CsCl termination of CsPbCl_3_, rather than the PbCl_2_ termination observed in experiments. However, the PbCl_2_-terminated model ranks as the second best for the (100)//(010)–CsPbCl_3_/Pb_4_S_3_Cl_2_ interface, with an *E*_int_ of 27 meV Å^–2^ well below that of all the other interface models (dashed line in Figure 6a). This discrepancy in the surface termination is likely due to the tendency of perovskite nanocrystals to express a lead-rich surface,^114−116^ which would provide a kinetic advantage to the growth of PbCl_2_-terminated interfaces. However, other kinetic effects stemming from interactions with the reaction medium, that are not considered in our simulations, might play a role as well.

The most stable interface found for the competing Pb_3_S_2_Cl_2_ phase is the (100)//(210), with *E*_int_ = 50 meV Å^–2^. A visual inspection of the interface connectivity, shown in Figure 6c, reveals the presence of several dangling bonds and undercoordinated ions, which explains the lower stability. As the energy of the most stable interface between CsPbCl_3_ and Pb_3_S_2_Cl_2_ is significantly higher than that of the (100)//(010)–CsPbCl_3_/Pb_4_S_3_Cl_2_ experimental interface, we conclude that Pb_3_S_2_Cl_2_ is unlikely to grow on the surface of the CsPbCl_3_ seeds because it cannot outcompete the more favorable epitaxial match with Pb_4_S_3_Cl_2_. This case demonstrates how Ogre can be used to predict the likely outcome of a synthesis when there are several competing phases, one of which is favored by epitaxial templating.

### 2.3 Using Ogre to Interpret Experimental Interfaces

Another challenge posed by nano-heterostructures is the identification of interfaces obtained experimentally. For example, the identification of the two materials involved can be hindered by the overlap of broad X-ray diffraction signals, and sample-averaged compositional analyses might provide limited information due to the presence of multiple compounds. Such issues can be mitigated with spatially resolved techniques like HR-STEM coupled with energy dispersive X-ray spectroscopy (EDXS). However, these methods have large uncertainties under realistic operational conditions (∼5–10%, depending on the elements), and can be misled by cross-element spectral overlap or the residual presence of unreacted precursors, which can increase the concentration measured for some of the elements.^74^

Even when the two domains of a heterostructure can be identified, the question remains of whether or not the interface is epitaxial, and if so, what structure it adopts. Atomic-resolution images of the interface may provide a definitive answer, but these are challenging to acquire and require advanced instrumentation. For this reason, it is common practice to label interfaces as epitaxial based on matching spots in the Fourier transform of lattice-resolved TEM images.^34,77,117^ However, this does not provide direct evidence of commensurate matching at the interface and can be easily misinterpreted. In such situations, Ogre can help identify interface models that are in agreement with the available experimental data. To exemplify this, we revisit some published colloidal heterostructures,^34,36,39,76,77^ aiming to suggest a plausible interface structure based on information provided in the original publications. We focus on CsPbBr_3_ because of the wide availability of studies and relevance for the colloidal chemistry community.

**Figure 7:**
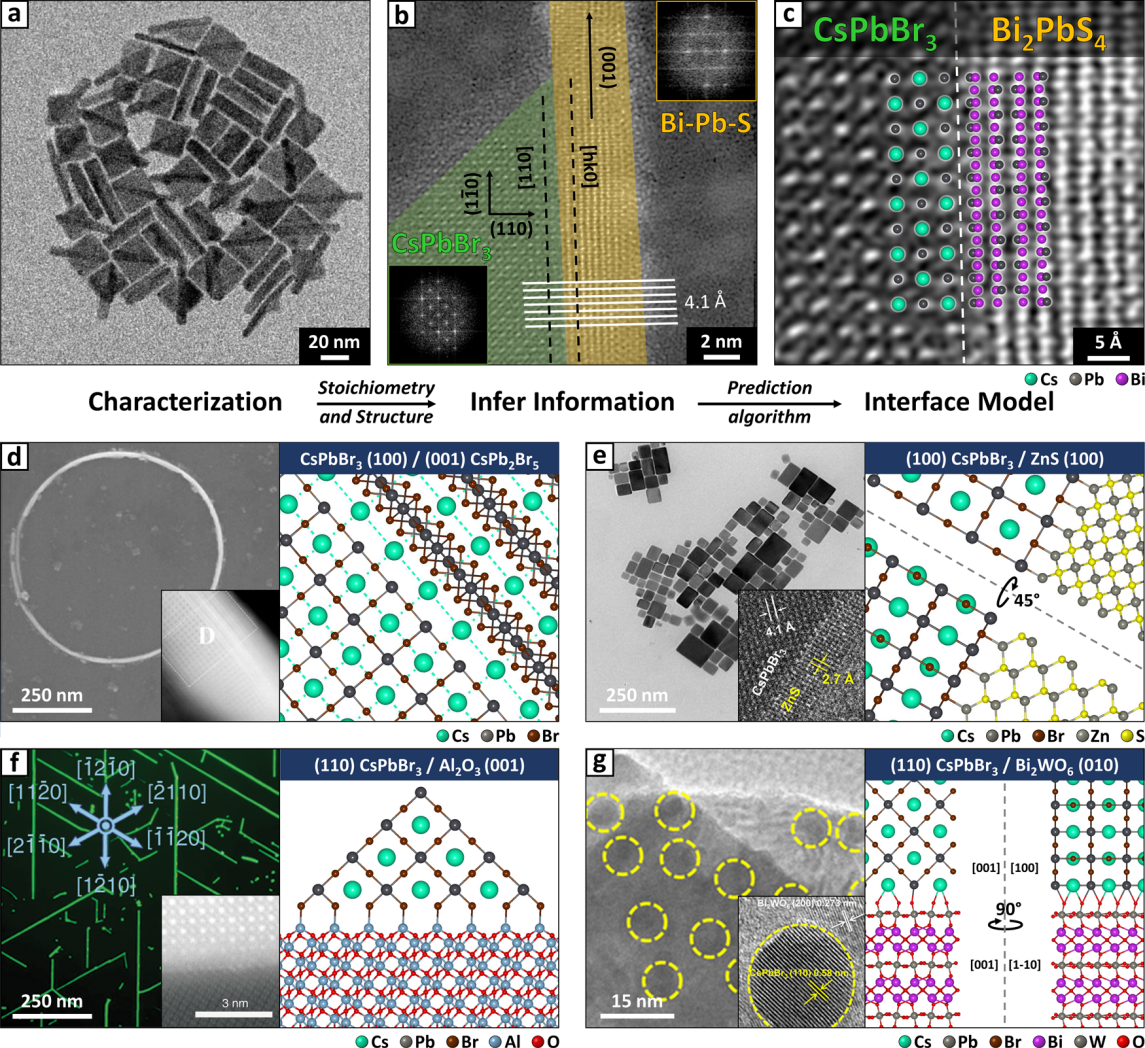
Heterostructures involving CsPbBr_3_. (a) Low-resolution TEM image of CsPbBr_3_/Bi_*x*_Pb_*y*_S_*z*_ heterostructures. (b) Lattice-resolved TEM image of a CsPbBr_3_/Bi_*x*_Pb_*y*_S_*z*_ heterostructure, showing the flat epitaxial interface. The relative orientation of CsPbBr_3_ and Bi_*x*_Pb_*y*_S_*z*_ domains is narrowed down via the Fourier transform analysis of their lattices (insets). Model of the (110)//(100)–CsPbBr_3_/Bi_2_PbS_4_ galenobismuthite interface proposed by Ogre superimposed on a magnified HR-STEM image of the interface (adapted with permission,^39^ Copyright 2023, the Authors). (d–g) Images of other heterostructures involving CsPbBr_3_ (left and inset), compared with models of the interface produced by Ogre (right). Adapted with permission.^34,36,76,77^ From left to right: (100)//(001)–CsPbBr_3_/CsPb_2_Br_5_ (d, Copyright 2020, Springer Nature);^36^ (100)//(100)–CsPbBr_3_/ZnS (e, Copyright 2020, American Chemical Society),^34^ (110)//(001)–CsPbBr_3_/Al_2_O_3_ (f, Copyright 2020, the Authors),^76^ (110)//(001)–CsPbBr_3_/Bi_2_WO_6_ (g, Copyright 2020, American Chemical Society).^77^

The first example is the CsPbBr_3_/Bi_*x*_Pb_*y*_S_*z*_ heterostructures recently reported by some of us (Figure 7a).^39^ These comprise a CsPbBr_3_ domain supporting the growth of a lead–bismuth sulfide rod, whose composition could not be determined conclusively. Indeed, the Bi–Pb–S system includes at least five phases with similar stoichiometries and structures: Bi_2_Pb_6_S_9_ heyrovskyite, the Bi_2_Pb_3_S_6_ polymorphs lillianite and xilingolite, Bi_2_Pb_2_S_5_ cosalite, and Bi_2_PbS_4_ galenobismuthite. Moreover, Bi_2_S_3_–PbS solid solutions with nonstoichiometric compositions can also form.^118^ The challenge was compounded by the presence of lead in both domains of the heterostructure along withits spectral overlap with sulfur in EDXS, which made the reported compositional analysis unreliable, yielding a noncharge-balanced stoichiometry of 19.6% Pb, 29.2% Bi, and 51.2% S.^39^

Lattice-resolved TEM images of the heterostructures pointed to the formation of a sharp epitaxial interface. Their Fourier transform was exploited to identify the orientation of the CsPbBr_3_ domain and determine that it exposes the (110) facet at the interface, as shown in Figure 7b. In this orientation the (22̅0) planes of CsPbBr_3_ are perpendicular to the interface, and their ∼4.1 Å periodicity matches with that of the (001) planes of all the Bi_*x*_Pb_*y*_S_*z*_ phases considered, which share similar columnar structures with *c* as the preferred growth axis. It was also established that the *c* axis of Bi_*x*_Pb_*y*_S_*z*_ is parallel to the interface, meaning that the sulfide rod matches the perovskite with a (*hk*0) plane. Based on this evidence, we constrained the search to a (110)//(*hk*0)–CsPbBr_3_/Bi_*x*_Pb_*y*_S_*z*_ interface, with the additional requirement that the [11̅0] lattice vector of CsPbBr_3_ and the [001] vector of Bi_*x*_Pb_*y*_S_*z*_ must be parallel (i.e., [11̅0]
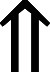
[001]). This allowed us to lower the number of epitaxial relations between CsPbBr_3_ and the five Bi_*x*_Pb_*y*_S_*z*_ phases considered from more than 100 (area based on eq 1, strain ≤10%, *h,k,l* ≤ 2) to just 10 supercells that meet all the requirements (see Figure S26 and related discussion).

After performing *surface matching and ranking*, the (110)//(100)–CsPbBr_3_/Bi_2_PbS_4_ interface with galenobismuthite emerges as the most stable, with *E*_int_ = 46 meV Å^–2^ (see Figures S27 and S28 and Table S13 for a full account of the results). This interface appears well-connected (see Figure S27), and the model accurately captures the positions of heavy atoms in the Bi_*x*_Pb_*y*_S_*z*_ rod observed by atomic-resolution TEM, supporting the identification of galenobismuthite (Figure 7c). Two additional candidate interfaces between CsPbBr_3_ and the (100) and (110) planes of Bi_2_Pb_2_S_5_ cosalite were found to be only slightly less stable. However, we consider them unlikely owing to the significantly higher strain and interface area, and because the atom positions do not match the experiment as closely as the galenobismuthite (see Figure S27). Notably, Patra et al. have later independently reported similar heterostructures, which they also identified as CsPbBr_3_/Bi_2_PbS_4_ galenobismuthite, thus reinforcing our findings.^119^ We remark that this conclusion would have been challenging to extract from TEM images alone because all Bi_*x*_Pb_*y*_S_*z*_ phases feature similar columns of heavy elements, whose apparent spacing under TEM depends on the orientation of the crystal.

A similar approach of exploiting morphological data to inform simulations was adopted to reassess the interfaces shown in Figure 7d–g, reported in refs (34,36,76,77). In all of these cases, the epitaxial relations proposed in the original studies lead to small commensurate domains with reasonable strain and supercell area in Ogre’s lattice matching step (see Figures S29–S31). Therefore, we proceed with the proposed interface orientation and focus on providing a plausible model of the interface. For instance, the lattice-resolved image of a nanoring in Figure 7d points to a (100)//(001)–CsPbBr_3_/CsPb_2_Br_5_ interface,^36^ for which the most stable model produced by Ogre (see Figure S29 and Table S14) is consistent with the empirical principle of Cs^+^-sublattice continuity observed for cesium lead halide heterointerfaces (marked by dashed cyan lines).^120^ For the CsPbBr_3_/ZnS particles in Figure 7e,^34^ our findings (see Figure S30 and Table S15) confirm that ZnS can passivate the facets of perovskite nanocubes by forming a (100)//(100) epitaxial shell, despite their different crystal structures.^13^ Finally, Figure 7f provides an example of epitaxy on a single-crystalline substrate, where the CsPbBr_3_ microwires grow along the lattice vectors of Al_2_O_3_.^76^ The resulting (110)//(001)–CsPbBr_3_/Al_2_O_3_ relation (see Figure S31 and Table S16) features remarkably high strain and supercell area (see Table S5), which is likely possible thanks to the softness of halide perovskites.^121,122^

The case of Bi_2_WO_6_/CsPbBr_3_ heterostructures in Figure 7g is different,^77^ because the TEM images do not indicate a clear epitaxial relation between the tungstate and the perovskite. Indeed, ref (77) does not advance claims on the nature of the interface. However, the morphology of Bi_2_WO_6_ offers some clues, as this material tends to grow in wide nanosheets that exposethe (010) surface due to its layered crystal structure.^123^ Moreover, the lattice fringes indexed by the authors of ref (77) (Figure 7g, inset) suggest a (010)//(110)–Bi_2_WO_6_/CsPbBr_3_ interface. Indeed, this orientation produces a promising match with a 5.0% strain and an area of 90 Å^2^ in Ogre’s *lattice matching* step (see Figure S32). Following *surface matching and ranking*, the proposed (010)//(110)–Bi_2_WO_6_/CsPbBr_3_ orientation produces a set of well-connected interface models. Figure 7g shows the third most stable (*E*_int_ = 293 meV Å^–2^) because it presents an oxide-rich termination for Bi_2_WO_6_, which we consider more likely based on chemical and kinetic considerations. Although the two top candidates are energetically more favorable (*E*_int_ = 248 and 257 meV Å^–2^, see Table S17), their formation would require a Bi termination for the tungstate, which we deem experimentally unlikely because the perovskite was grown on preformed Bi_2_WO_6_ nanosheets that are likely oxygen-terminated.^77^ Unfortunately, in the absence of atom-resolved TEM images it is not possible to validate this hypothesis.

### 2.4 Validation for Oxide Interfaces

**Figure 8:**
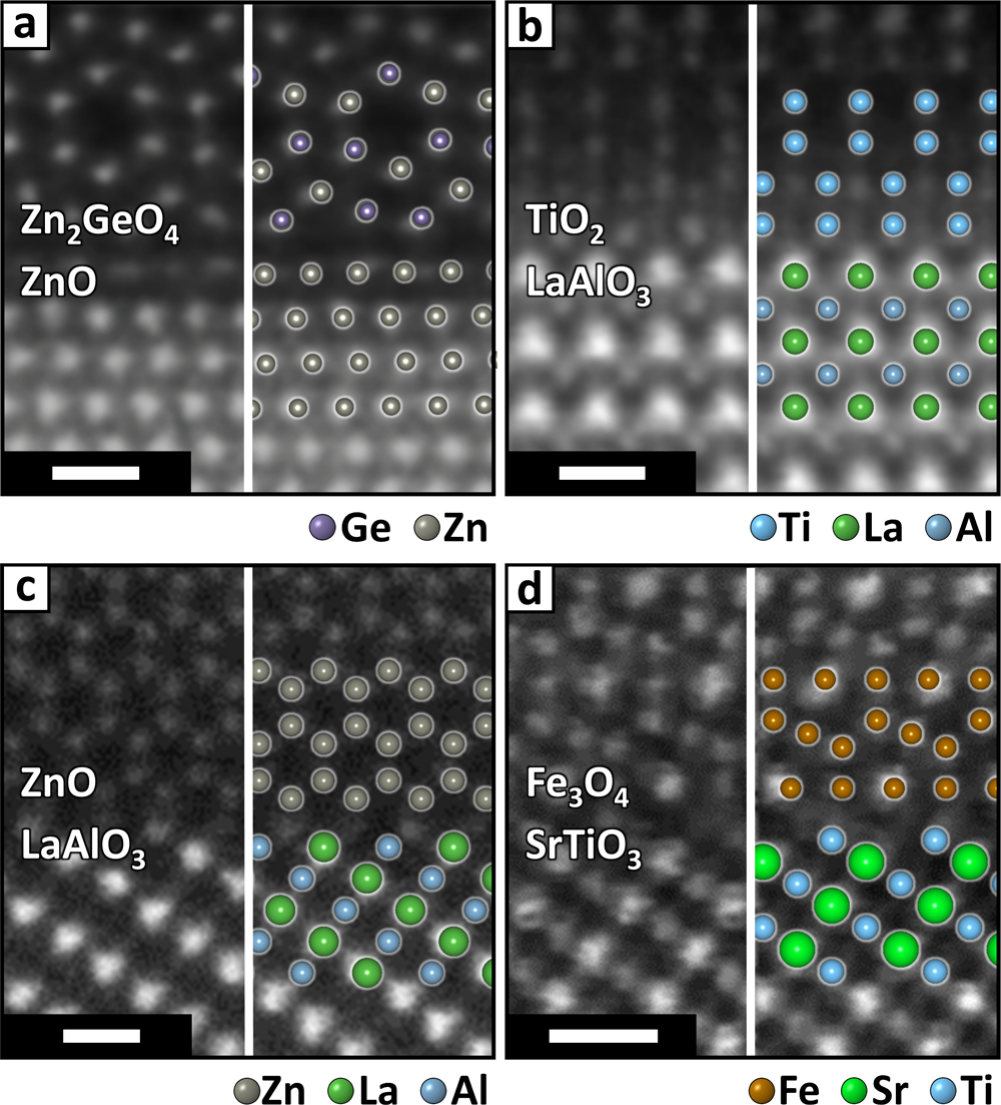
Validation for oxide interfaces. Atomic resolution images of epitaxial interfaces from the literature with the structure predicted by Ogre superimposed. TEM images adapted with permission.^78−81^ Left to right, top to bottom: (001)||(1̅10)–ZnO/Zn_2_GeO_4_ [a, Copyright 2019, IOP Publishing];^78^ (100)//(001)–LaAlO_3_/TiO_2_ [b, Copyright 2022, the Authors];^79^ (112)//(100)–LaAlO_3_/ZnO [c, Copyright 2013, Elsevier B.V.];^80^ (1̅11)//(111)–Fe_3_O_4_/SrTiO_3_ [d, Copyright 2016, the Authors].^81^ All models were scaled to match the TEM images without adjusting epitaxial registry and interfacial distance. Oxygen atoms are omitted to ease the comparison with electron scattering contrast. The contrast and sharpness of TEM images have been adjusted to enhance the visibility of atoms. All scale bars are 5 Å.

To showcase Ogre’s applicability beyond metal halides, we assess its performance for a set of known oxide–oxide interfaces, which are the most widely studied class of polar materials for heteroepitaxy.^78−81^ We focus on thin films because colloidal heterostructures are not as common for oxides, which limits the availability of high quality atomic-resolution images. For all the examples presented in Figure 8 we adopt the epitaxial orientation reported in the original publication, for which Ogre indeed finds low strain and a small supercell area (see 2D-supercells in Figures S33–S36). Subsequently, the interface models produced by the *surface matching and ranking* procedure (see Tables S18–S21) were compared to the experimental TEM images. All models were scaled isotropically without adjusting the epitaxial registry and interfacial distance. The small differences in the atomic positions may be attributed to strain, image aberrations, and the approximations used in the classical potential for predicting interface distances.

In three out of four cases (Figure 8a–c, see also Figures S33–S35 and Tables S18–S20), the model ranked as the most stable by Ogre yields the best match to the experimental TEM images. The sole exception is the SrTiO_3_/Fe_3_O_4_ interface (Figure 8d), for which the model ranked second, featuring an oxygen-rich surface for the Fe_3_O_4_ domain, aligns better with the experiment than the top-ranking model, where Fe_3_O_4_ is iron-terminated (see Figure S36 and Table S21). As noted above for the (100)//(010)–CsPbCl_3_/Pb_4_S_3_Cl_2_ interface, Ogre does not consider growth kinetics, reaction conditions (e.g., temperature, pressure), and interactions with the environment (e.g., the reaction atmosphere or solvent). Therefore, Ogre may predict interface configurations that are thermodynamically stable, but not kinetically favorable under the specific experimental conditions. This means that Ogre may fail to predict the outcome of epitaxial growth when the structure adopted by an interface is strongly influenced by factors that are beyond the reach of these simulations, such as the native surface passivation of the substrate or the reactivity of precursors. Conversely, Ogre may offer valuable insights for optimizing the conditions of an experiment to promote the growth of a particular interface. For example, learning that the SrTiO_3_/Fe_3_O_4_ interface can adopt two competing structures, differentiated by the presence of oxygen at the surface of Fe_3_O_4_, might prompt one to condition the substrate with oxygen or, conversely, apply a reducing pretreatment to select between the two structures.

### 2.5 User Best Practices

Finally, for the benefit of prospective users, we provide a series of guidelines for assessing whether Ogre is the correct tool for their needs and help them make the best use of the code:

1. *Scope of simulations.* Ogre is designed to identify epitaxial relations between two given materials and propose plausible interface models. The simulations can inform the synthesis of new systems, help rationalize the results of experiments, and serve as a starting point for more advanced simulations based on DFT. Be mindful that Ogre does not account for variables like temperature, pressure, interactions with the reaction medium and kinetics.
2. *Selection of input CIFs.* Ogre does not check the input structures provided by the user. Hence, unreliable CIFs may lead to unreliable lattice matching results and classical potential parameters. The user is advised to check the temperature and pressure at which the structure was refined, as they influence the cell parameters and can lead to polymorphs. CIFs with partial occupancies are not supported.
3. *Lattice matching.* Select the (*hkl*) indices based on the surfaces exposed by the seed/substrate, and allow higher strain for nanomaterials. Interfaces with small supercells are more likely to succeed, even when this leads to(reasonably) higher strain. The *lattice matching* step works also for nonpolar materials, as interactions are not considered.
4. *Surface matching and ranking, assessment of results.* The classical potential implemented here is only applicable to polar materials, in which formal charges can be assigned to atoms. The *E*_*int*_ values it produces are valid only for relative comparisons, and DFT calculations are recommended for further refinement. We also advise the user to inspect the final models and ensure that they are chemically sound, as Ogre only verifies numerical convergence. We recommend watching out for undercoordinated ions, and for ions of the same sign that are found too close to each other at the interface.

## 3 Conclusions

Given two materials, Ogre identifies domain-matched epitaxial relations and produces structural models of the corresponding interfaces. The prediction workflow consists of three steps: *lattice matching, interface generation,* and *surface matching and ranking*. In the *lattice matching* step, combinations of lattice planes from the two materials are scanned to find commensurate epitaxial relations with low strain and supercell area. For the orientations that provide promising epitaxial matches, the *interface generation* step produces candidate models by combining all the possible surface terminations for the two materials. Finally, the *surface matching and ranking* step determines the optimal epitaxial registry and interfacial distance for each interface model, and ranks their stability by evaluating the interface energy.

A significant advancement is the implementation of two developments specifically intended for the fast prediction of interfaces between ionic or polar materials. The first is a preliminary screening at the *interface generation* stage based on charge balancing, which reduces the number of models by eliminating candidates that would be unstable due to repulsive electrostatic interactions at the interface. The second is a classical electrostatic potential parametrized based on the bulk structures of the two materials, which allows to optimize and rank the interface models at a fraction of the computational cost of DFT. We note that our classical potential is designed for ionic and polar materials, and is not intended for interfaces involving covalent, metallic, and van der Waals materials (alternative methods are available in Ogre for these cases). Thanks to these advancements, the full prediction workflow for polar interfaces can now be executed in just a few minutes on a simple laptop. To further lower the accessibility barrier for nonspecialist users, the Ogre code can also be executed using the desktop application *OgreInterface*, available for Windows, Linux and Mac (Figures S37–S39, see Data Availability for the installation wizards).

To demonstrate its utility, we applied Ogre to a wide variety of polar interfaces, with a focus on colloidal nano-heterostructures formed by lead halide perovskites and oxide thin films grown by physical deposition methods. In the case of known interfaces, Ogre’s predictions aligned well with experimental evidence, confirming its reliability. In cases where the nature of the interfaces was not yet fully established, Ogre provided useful insights to elucidate experimental observations. For example, we used Ogre to explain the formation of a specific epitaxial interface in the presence of many potential competitors, and we were able to propose plausible interface models for colloidal heterostructures that were reported but not fully characterized hitherto. Finally, the interface structures produced by Ogre can be used as starting models to pursue further geometry relaxation, stability evaluation, and prediction of electronic, magnetic, and topological properties by DFT.^124−128^

Based on the results presented here, we propose Ogre as a quick and user-friendly tool for designing experiments and assigning structures to the resulting interfaces. Example applications include assessing if two materials are likely to form a heterostructure, selecting the most promising substrate for growing a target material, and providing atomistic models to support the interpretation of high-resolution TEM images when new interfaces are obtained. Finally, we envision Ogre as a component of high-throughput workflows for the exploration of prospective epitaxial interfaces, where it may be used for the screening of material pairs from databases of inorganic structures.

We thus conclude that Ogre is a powerful, versatile, and accessible tool for the structure prediction of epitaxial interfaces between polar materials, with great potential for the discovery of new interfaces and the interpretation of experimental results. In the hands of experimentalists, Ogre could significantly boost advancements in all fields that rely on epitaxial interfaces. This includes the rapidly expanding field of colloidal nanoheterostructures, as well as epitaxial interfaces grown by thin film deposition methods for applications in electronic devices and photocatalysis.

## 4 Methods

### 4.1 DFT Calculations

DFT calculations were performed using the Vienna ab Initio Simulation Package (VASP) version 6.4.2^129−133^ with the projector-augmented wave (PAW) method.^129,134^ The strongly constrained and appropriately normed (SCAN) meta-generalized gradient approximated (meta-GGA) was employed for the description of the exchange-correlation interactions between electrons, and the revised Vydrov-van Voorhis (rVV10) nonlocal correlation functional was used to describe van der Waals interactions. The relevant VASP INCAR tags for SCAN+rVV10 are METAGAA = SCAN, LUSE_VDW = True, BPARAM = 15.7, CPARAM = 0.0093, and LASPH = True.^107^ A plane-wave cutoff of 350 eV was used. A Monkhorst *k*-point grid with a density of 3.5 points per Å^–1^ was used to sample the Brillouin zone. All calculations were converged to a total energy change of less than 1 × 10^–5^ eV (EDIFF = 1 × 10^–5^). In all calculations involving slab structures (i.e., surfaces and interfaces), dipole corrections were applied along the *c*-axis (IDIPOL = 3, and LDIPOLE = True),^135^ and a vacuum region of 60 Å was added to avoid interactions between periodic images along the surface normal. All CsPbBr_3_ slabs were 31.8 Å thick, and all Pb_4_S_3_Br_2_ slabs were 41.7 Å thick for both interface and stand-alone surface models. No surface passivation was applied to any slab or interface model in this study.

## Data Availability

For reproducibility, the version of Ogre used for this work (1.2.16) is available on GitHub at the link https://github.com/DerekDardzinski/OgreInterface/tree/ionic_heterostructures_paper. The most updated version of the *OgreInterface* library can be installed using the Python Package Index (PyPI). Other versions of Ogre are available for download from www.nomarom.com. The raw output of all Ogre simulations in this work is available on Zenodo at the link https://zenodo.org/records/13472738. This repository also contains: Python scripts required to reproduce our simulations, installation wizards for the *OgreInterface* desktop application (available for Windows, Linux, and Mac).

## Supporting Information Available

Comparison of cubic vs orthorhombic CsPbBr_3_; tabulated summary of the interfaces discussed in the main text; expanded description of the implementation of lattice matching in Ogre; analysis of other interfaces reported for CsPbBr_3_/Pb_4_S_3_Br_2_; expanded description of interface generation in Ogre, including atom clustering and slab surface charges; expanded description of surface matching and ranking, including details of the Ogre classical potential; comparison of the classical potential ranking to DFT for the (100)//(010) – CsPbBr_3_/Pb_4_S_3_Br_2_ interface; expanded analysis of lead sulfochloride/CsPbBr_3_ interfaces; expanded analysis of Bi_x_Pb_y_S_z_/CsPbBr_3_ interfaces; expanded analysis of additional interfaces with CsPbBr_3_; expanded analysis of the oxide interfaces shown in the main text; introduction to the *OgreInterface* app; evaluation of the computational performance of Ogre on a consumer-grade laptop (PDF)

## Notes

A preprint of this paper is available: Toso, Stefano; Dardzinski, Derek; Manna, Liberato; Marom, Noa. “Fast Prediction of Ionic Epitaxial Interfaces with Ogre Demonstrated for Colloidal Heterostructures of Lead Halide Perovskites”, 2024, DOI: 10.26434/chemrxiv-2024-hwthh. ChemRxiv. URL: https://chemrxiv.org/engage/chemrxiv/article-details/66d02dbea4e53c4876fb4e61 (accessed January 11, 2025).
