# Supplementary material for: Structure Prediction of Ionic Epitaxial Interfaces with Ogre Demonstrated for Colloidal Heterostructures of Lead Halide Perovskites
Source: ACS Nano. 2025 Feb 2;19(5):5326–41. doi: 10.1021/acsnano.4c12713 (PMC11823643; doi:10.1021/acsnano.4c12713)
Supplement: Supplementary file 1 — nn4c12713_si_001.pdf [file nn4c12713_si_001.pdf]

Supporting Information for

Structure Prediction of Ionic Epitaxial Interfaces with Ogre Demonstrated for Colloidal Heterostructures of Lead Halide Perovskites

Stefano Toso[1,†], Derek Dardzinski[2,†], Liberato Manna[1], Noa Marom,[2,3,4]**

[1] Nanochemistry Department, Istituto Italiano di Tecnologia, Genova, 16163, Italy

[2] Department of Materials Science and Engineering, Carnegie Mellon University, Pittsburgh, PA, 15213, United States

[3] Department of Physics, Carnegie Mellon University, Pittsburgh, PA, 15213, United States

[4] Department of Chemistry, Carnegie Mellon University, Pittsburgh, PA, 15213, United States

† These authors contributed equally

* E-mail: liberato.manna@iit.it, nmarom@andrew.cmu.edu

S1. Cubic vs orthorhombic $CsPbBr_3$ 2

S2. Interfaces discussed in the Main Text 9

S3. Lattice matching 10

S4. Other $CsPbBr_3$/$Pb_4S_3Br_2$ reported interfaces 14

S5. Interface generation 20

S6. Surface Matching and Ranking 26

S7. Electrostatic potential vs DFT 32

S8. Lead sulfochloride/$CsPbBr_3$ interfaces 33

S9. $Bi_xPb_yS_z$/$CsPbBr_3$ interfaces 36

S10. Other interfaces with $CsPbBr_3$ 40

S11. Validation on oxide interfaces 44

S12. Introduction to the OgreInterface app 47

S13. Ogre performances on a consumer-grade laptop 50

S14. Supplementary References 50

S1. Cubic vs orthorhombic $CsPbBr_3$

S1.1. Cubic vs orthorhombic description for CsPbX3 perovskites.

All $CsPbX_3$ (X = Cl, Br, I) perovskites are orthorhombic at room temperature, both in bulk form[1–3] and as nanocrystals.[4] They crystallize in the space group #62, for which both the standard setting *P*nma and the non-standard setting *P*bnm are adopted in the literature. In this work, the reference structure for $CsPbBr_3$ is the *P*nma ICSD-143617.[1] However, this structure differs from the ideal cubic prototype only for mild distortions. Hence, crystallographic features (*i.e.*, lattice directions and planes) that in the orthorhombic setting are technically not equivalent are nevertheless extremely similar. Therefore, we chose to adopt the pseudocubic structure for all the predictions presented in the Main Text. **Table S1** allows to convert relevant Miller indices from the pseudocubic *P*m-3m to the orthorhombic *P*nma and *P*bnm settings.

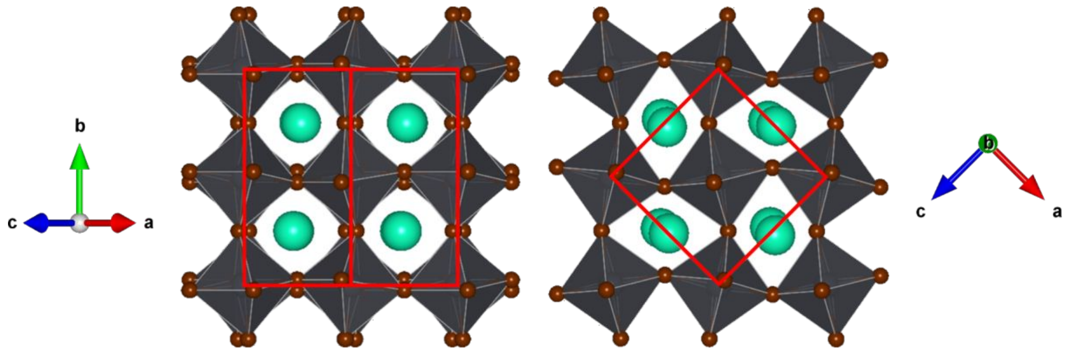

Figure S1. $CsPbBr_3$ structure. The orthorhombic structure of $CsPbBr_3$ (*P*nma) as seen from two non-equivalent lattice directions: [101] and [010]. Their similarities are effectively captured by the pseudocubic notation [100], where they become equivalent.

Table S1. $CsPbBr_3$ orthorhombic to pseudocubic conversion.

Pseudocubic (*P*m-3m)	Orthorhombic - standard (*P*nma)	Orthorhombic – non-standard (*P*bnm)
(100)	(101) ; (020)	(110) ; (002)
(110)	(121) ; (200) ; (002)	(112) ; (200) ; (020)
(111)	(220) ; (022)	(202) ; (022)

Adopting the cubic setting simplifies the discussion, as planes that are different in the orthorhombic setting become equivalent in the cubic setting (**Figure S1**). For consistency, the pseudocubic lattice parameter was calculated from the *P*nma reference through **Equation S1**:

$$a_{PC} = \sqrt[3]{\frac{Z_{Pm\bar{3}m} \cdot V_{Pnma}}{Z_{Pnma}}} = \sqrt[3]{\frac{1 \cdot 796.24\ \text{Å}^3}{4}} = 5.839\ \text{Å} \qquad \textbf{(S1)}$$

S1.2. Control simulations with Pnma CsPbBr3

To assess the impact of choosing the pseudocubic *P*m-3m over the *P*nma structure for $CsPbBr_3$, we repeated the simulations for our $CsPbBr_3/Pb_4S_3Br_2$ test interface in both settings. Since the orthorhombic structure offers two similar planes, we eventually compared three different interface models, denoted as follows:

- (100)//(010) – $CsPbBr_3/Pb_4S_3Br_2$ [*P*m-3m cubic setting]
- (010)//(010) – $CsPbBr_3/Pb_4S_3Br_2$ [*P*nma orthorhombic setting, orientation 1]
- (101)//(010) – $CsPbBr_3/Pb_4S_3Br_2$ [*P*nma orthorhombic setting, orientation 2]

S1.2.1 Cubic vs Orthorhombic lattice matching results

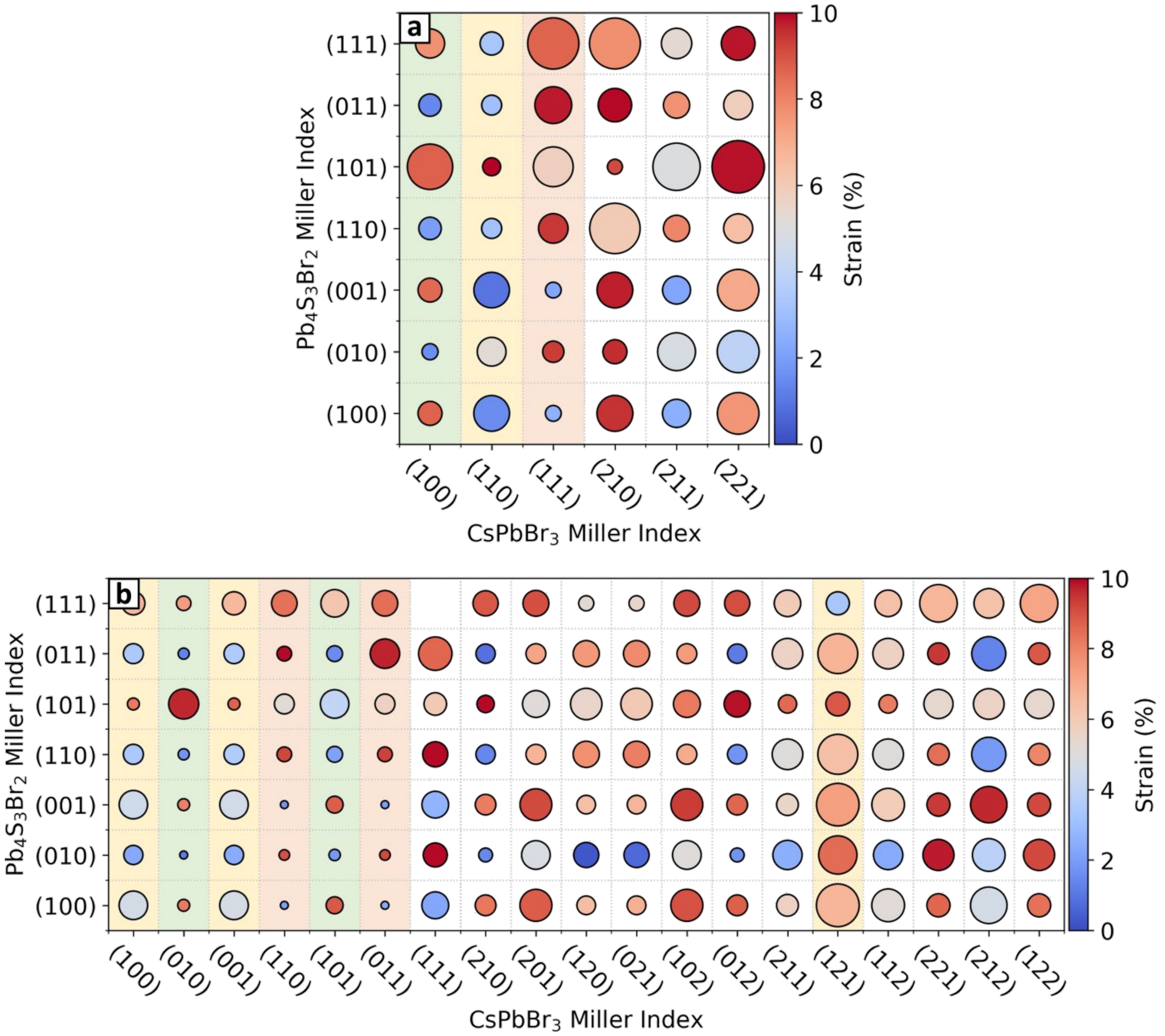

Figure S2. *P*m-3m vs *P*nma lattice matching. Lattice matching results for $CsPbBr_3/Pb_4S_3Br_2$ with the perovskite domain described as cubic (a) and as orthorhombic (b). Colored columns help visualize how one Miller index for the *P*m-3m cubic setting splits into similar, but not identical sets of matches for the *P*nma orthorhombic setting.

S1.2.2 Cubic: (100)∥(010) – $CsPbBr_3/Pb_4S_3Br_2$

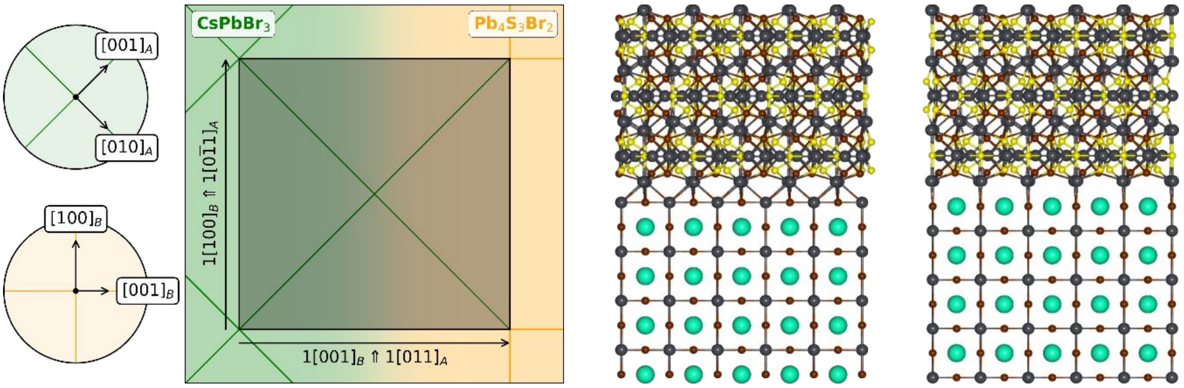

Figure S3. (100)∥(010) – $CsPbBr_3/Pb_4S_3Br_2$ interface (*P*m-3m). 2D-supercell (left), $PbBr_2$-terminated model (center) and CsBr-terminated model (right) of the interface. Corresponding data are highlighted in **Table S2** (green = $PbBr_2$ termination, blue = CsBr termination). The letters in the supercell identify the two materials: *A* = first material of the pair (here $CsPbBr_3$), *B* = second material of the pair (here $Pb_4S_3Br_2$). This notation is used consistently throughout the work. The $[hkl]_A⇈[h'k'l']_B$ notation at the edges of the 2D-supercells identifies lattice vectors that lie in the plane of the interface and are parallel to each other. It is adopted in conjunction with the $(hkl)_A∥(h'k'l')_B$ notation for planes parallel at the interface to unambiguously identify the relative orientation of the two materials. This notation is also adopted in **Table S5**. Reference structure: CCDC-2181721 ($Pb_4S_3Br_2$).

Table S2. *Interface ranking* results. Green = $PbBr_2$-terminated interface model. Blue = CsBr-terminated interface model. The indexes in the first two columns match the labels in **Figure 2**.

$Pb_4S_3Br_2$ slab index	$CsPbBr_3$ slab index	Interfacial dist. [Å]	$Pb_4S_3Br_2$ charge	$CsPbBr_3$ charge	E_{int} [meV Å^{-2}]
4	A	2.47	0	0	52
4	B	3.07	0	0	57
6	A	2.97	+2	0	75
2	A	3.24	-2	0	115
6	B	3.74	+2	0	118
3	A	3.00	-2	0	121
2	B	3.80	-2	0	126
5	A	3.35	+2	0	137
3	B	3.97	-2	0	138
5	B	3.64	+2	0	139
7	A	2.55	+4	0	332
7	B	3.14	+4	0	333
1	B	3.91	-4	0	347
1	A	4.01	-4	0	348

Notes: this is the interface discussed in the Main Text, **Figures 3-5**.

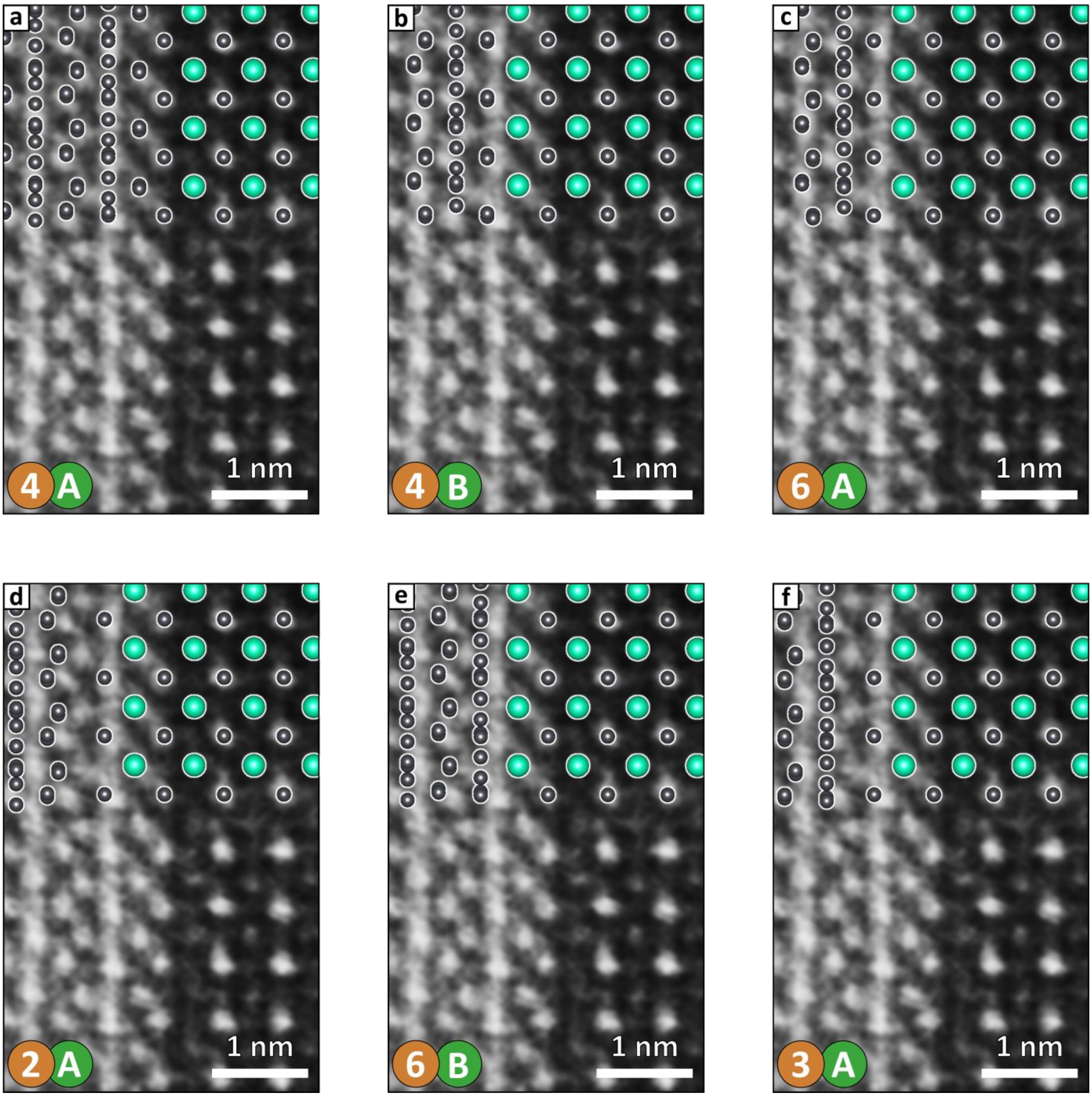

Figure S4. Top six ranked models for the (100)∥(010) – $CsPbBr_3/Pb_4S_3Br_2$ interface. The models were ranked by stability and labeled as shown in **Figure 4** of the Main Text, and are here superimposed to an atomic-resolution TEM image of a heterostructure. Only heavy atoms are shown to ease the comparison with electron scattering contrast (Pb = gray, Cs = cyan). Microscopy data adapted with permission.[5] Copyright 2023, American Chemical Society. Atoms color legend: Cs = cyan; Pb = gray; S = yellow; Br = brown.

S1.2.3 Orthorhombic 1: (010)∥(010) – $CsPbBr_3/Pb_4S_3Br_2$

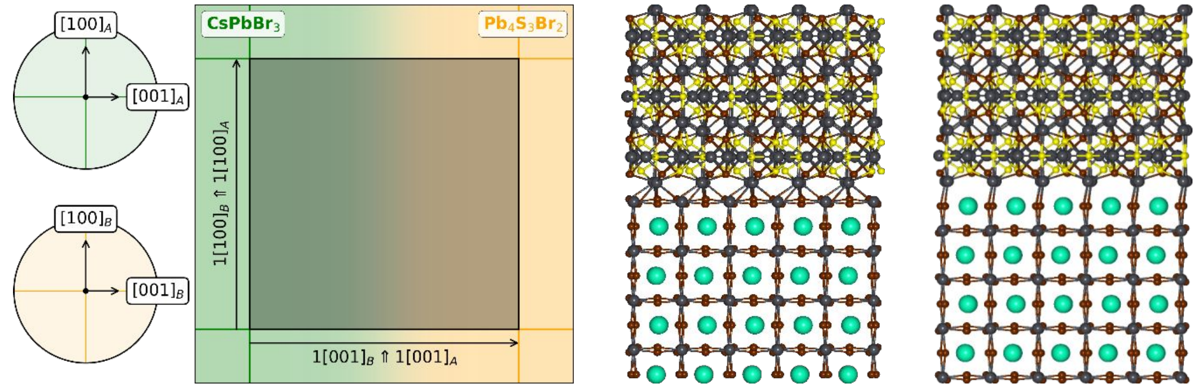

Figure S5. (010)∥(010) – $CsPbBr_3/Pb_4S_3Br_2$ interface (*P*nma). 2D-supercell (left), $PbBr_2$-terminated model (center) and CsBr-terminated model (right) of the interface. Corresponding data are highlighted in **Table S3** (green = $PbBr_2$ termination, blue = CsBr termination).

Table S3. *Interface ranking* results. Green = $PbBr_2$-terminated interface model. Blue = CsBr-terminated interface model.

$Pb_4S_3Br_2$ slab index	$CsPbBr_3$ slab index	Interfacial dist. [Å]	$Pb_4S_3Br_2$ charge	$CsPbBr_3$ charge	E_{int} [meV Å^{-2}]
0	0	2.06	0	0	43
0	1	3.08	0	0	58
3	0	2.70	+2	0	79
2	0	2.60	-2	0	109
3	1	3.42	+2	0	111
4	0	3.23	-2	0	121
4	1	3.37	-2	0	121
5	0	2.71	+2	0	126
2	1	3.54	-2	0	133
5	1	3.18	+2	0	134
6	1	2.78	+4	0	334
6	0	3.19	+4	0	334
1	1	3.51	-4	0	348
1	0	3.30	-4	0	349

Notes: the most stable model here is structurally equivalent to the $PbBr_2$-terminated model for the cubic setting (see **Paragraph S1.2.2**). The two interface models are also comparable by E_{int}.

S1.2.4 Orthorhombic 2: (101)⫽(010) – $CsPbBr_3/Pb_4S_3Br_2$

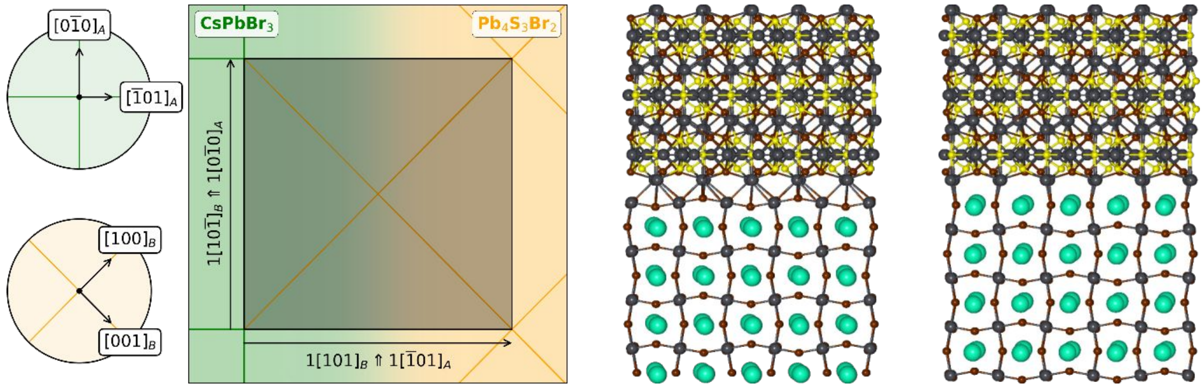

Figure S6. (101)⫽(010) – $CsPbBr_3/Pb_4S_3Br_2$ interface (*P*nma). 2D-supercell (left), $PbBr_2$-terminated model (center) and CsBr-terminated model (right) of the interface. Corresponding data are highlighted in **Table S4** (green = $PbBr_2$ termination, blue = CsBr termination).

Table S4. *Interface ranking* results. Green = $PbBr_2$-terminated interface model. Blue = CsBr-terminated interface model.

$Pb_4S_3Br_2$ slab index	$CsPbBr_3$ slab index	Interfacial dist. [Å]	$Pb_4S_3Br_2$ charge	$CsPbBr_3$ charge	E_{int} [meV Å^{-2}]
0	1	2.83	0	0	59
0	0	2.25	0	0	60
3	0	2.62	+2	0	88
3	1	3.27	+2	0	113
2	0	2.93	-2	0	123
4	1	2.52	-2	0	123
4	0	3.27	-2	0	124
5	0	2.83	+2	0	135
2	1	3.10	-2	0	137
5	1	3.45	+2	0	138
6	0	2.25	+4	0	333
6	1	2.93	+4	0	334
1	1	3.41	-4	0	349
1	0	3.93	-4	0	353

Notes: different from the two cases above, the most stable model here corresponds to a CsBr-termination for $CsPbBr_3$. However, the $PbBr_2$-terminated model is ranked second and with a negligible interface energy difference (0.3 meV Å^{-2}), indicating that in this setting the two interface models are equivalent in terms of stability.

S1.2.4 Cubic and Orthorhombic results compared

As seen in **Figure S7**, both the cubic and orthorhombic settings for the perovskite produced comparable models for interfaces formed by $PbBr_2$-terminated (top row) and CsBr-terminated (bottom row) slabs of $CsPbBr_3$. All models were comparable by E_{int}, with a mild preference for $PbBr_2$-terminated models in two cases out of three, suggesting that the two terminations are comparable in terms of stability. The fact that only the $PbBr_2$-terminated interface was reported experimentally is likely due to the influence of experimental factors,[5] such as the tendency of $CsPbBr_3$ nanocrystals to adopt a Pb-rich surface layer.[6,7] Overall, these results confirm that selecting the pseudocubic *P*m-3m setting instead of the more accurate but complex *P*nma description for $CsPbBr_3$ does not impact substantially the results of simulations.

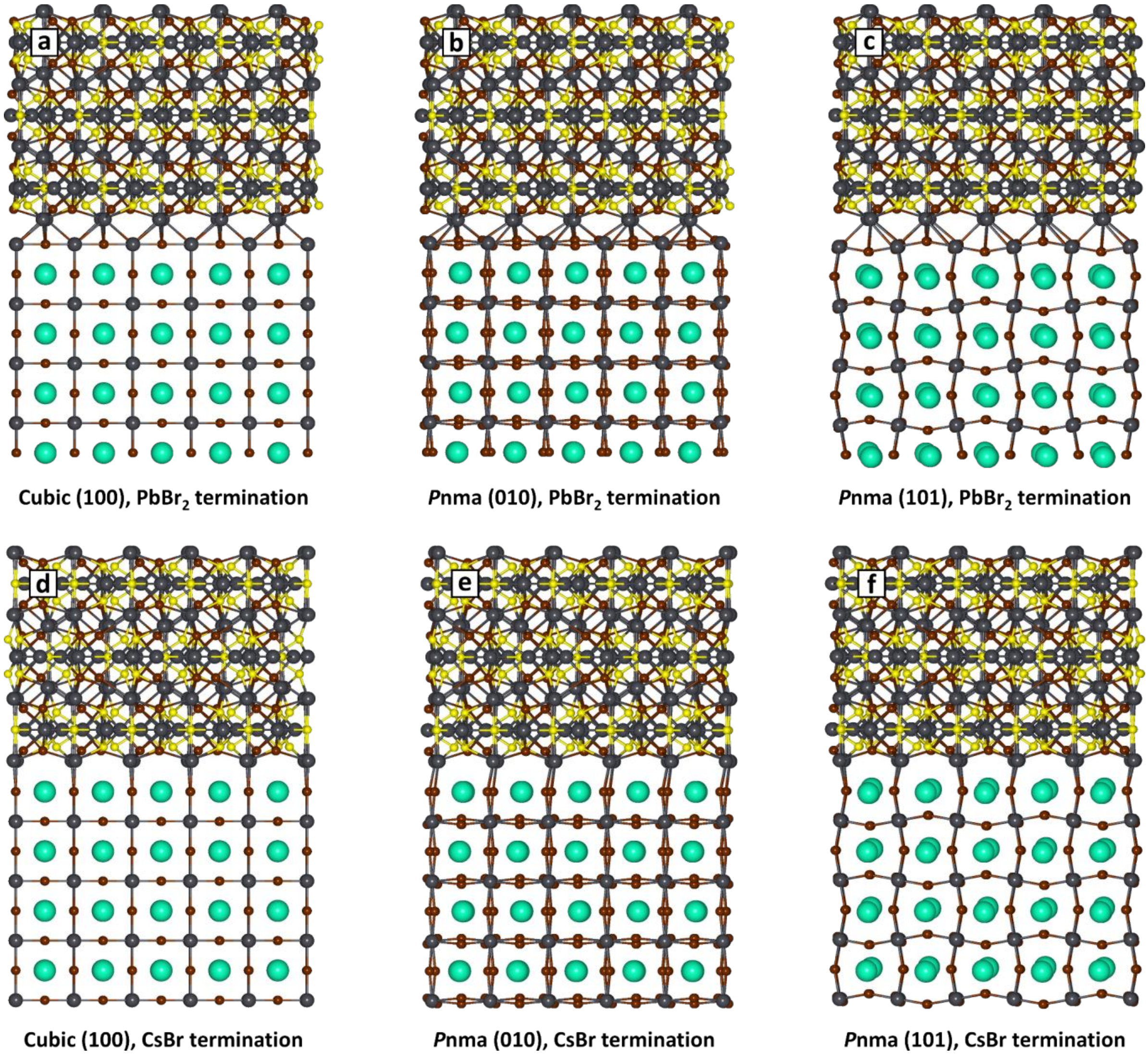

Figure S7. Interfaces formed by $Pb_4S_3Br_2$ with $PbBr_2$- and CsBr-terminated slabs of $CsPbBr_3$ in the cubic and orthorhombic settings examined. a-c) Interfaces formed by $PbBr_2$-terminated $CsPbBr_3$ slabs. d-f) Interfaces formed by CsBr-terminated $CsPbBr_3$ slabs.

S2. Interfaces discussed in the Main Text

Table S5. Experimental interfaces discussed in the Main Text. The $[hkl]_A$⇈$[h'k'l']_B$ notation indicates lattice vectors parallel to each other in the plane of the interface, and is included to avoid ambiguity in the choice of different supercells for the same $(hkl)_A$⫽$(h'k'l')_B$ pair.

Interface	$(hkl)_A$⫽$(h'k'l')_B$<br>$[hkl]_A$⇈$[h'k'l']_B$	Strain (%)	Area<br>(Å²)
$CsPbBr_3$ / $Pb_4S_3Br_2$<br>[Figs. 2-4, Ref. 5]	(100)⫽(010)<br>[011]⇈[001]	1.6	68
$CsPbCl_3$ / $Pb_4S_3Cl_2$<br>[Fig. 6, Ref. 8]	(100)⫽(010)<br>[011]⇈[001]	0.2	63
$CsPbBr_3$ / $Pb_3S_2Cl_2$<br>[Fig. 6, hypothetical]	(200)⫽(201)<br>[013]⇈[010]	9.4	314
$CsPbBr_3$ / Bi_2PbS_4<br>[Fig. 7a-c, Ref. 9]	(110)⫽(100)<br>[$1\bar{1}0$]⇈[001]	0.9	96
$CsPbBr_3$ / $CsPb_2Br_5$<br>[Fig. 7d, Ref. 10]	(100)⫽(001)<br>[011]⇈[$\bar{1}00$]	2.7	68
$CsPbBr_3$ / ZnS<br>[Fig. 7e, Ref. 11]	(100)⫽(100)<br>[010]⇈[001]	8.0	34
$CsPbBr_3$ / Al_2O_3<br>[Fig. 7f, Ref. 12]	(110)⫽(001)<br>[$1\bar{1}0$]⇈[$\bar{1}\bar{2}0$]	6.0	145
$CsPbBr_3$ / Bi_2WO_6<br>[Fig. 7g, Ref. 13]	(110)⫽(010)<br>[001]⇈[$00\bar{1}$]	5.0	96
ZnO / Zn_2GeO_4<br>[Fig. 8a, Ref. 14]	(001)⫽($\bar{1}10$)<br>[100]⇈[$1\bar{1}\bar{1}$]	11.9	82
$LaAlO_3$ / TiO_2<br>[Fig. 8b, Ref. 15]	(100)⫽(001)<br>[010]⇈[$\bar{1}00$]	0.2	14
$LaAlO_3$ / ZnO<br>[Fig. 8d, Ref. 16]	(112)⫽(100)<br>[$1\bar{1}0$]⇈[001]	2.2	35
Fe_3O_4 / $SrTiO_3$<br>[Fig. 8e, Ref. 17]	(-111)⫽(111)<br>[$0\bar{1}1$]⇈[$0\bar{1}1$]	7.5	31

S3. Lattice matching

S3.1. Quantification of strain

A common expression for strain at an epitaxial interface is shown in **Equation S2**:

$$S = \frac{|a_{epilayer} - a_{substrate}|}{a_{substrate}} \cdot 100\% \quad \textbf{(S2)}$$

where a indicates the lattice parameter. This is well-suited for epitaxial films involving cubic bulk structures, where the predominant role of the substrate makes it reasonable to select its lattice parameter as a reference. However, in colloidal heterostructures the distinction between substrate and epilayer is not always clear, and the strain can equally affect both materials at the interface. Hence, it is better to take as a reference the average of lattice parameters instead:

$$S = 2 \cdot \frac{|a_{epilayer} - a_{substrate}|}{a_{epilayer} + a_{substrate}} \cdot 100\% \quad \textbf{(S3)}$$

Nevertheless, **Equations S3-4** only work for the very simple case of two cubic materials being matched along the same lattice direction, that is $(hkl)_A = (h'k'l')_B$. This is a common situation for simple interfaces like sphalerite CdS/CdSe,[18] but cannot be applied to pairs of structurally diverse materials growing along arbitrary orientations. Therefore, we introduced a new strain parameter inspired by **Equation S3**, but based on the concept of 2D-supercells, that can be applied to any materials and crystallographic orientations.

In short, given a $(hkl)_A /\!/ (h'k'l')_B$ interface we apply the Zur-McGill algorithm[19] to construct a common 2D-supercell starting from the single-material 2D-cells of the $(hkl)_A$ and $(h'k'l')_B$ planes. As the 2D-supercell must describe the periodicity of both materials at the interface, it can be constructed from the lattice vectors of either. This results in two almost equivalent descriptions for the same supercell, that have as base vectors X_A and X_B respectively:

$$X_A = \begin{bmatrix} a_x^A & a_y^A \\ b_x^A & b_y^A \end{bmatrix} = \begin{bmatrix} \vec{a}_A \\ \vec{b}_A \end{bmatrix} \quad \textbf{(S4a)}$$

$$X_B = \begin{bmatrix} a_x^B & a_y^B \\ b_x^B & b_y^B \end{bmatrix} = \begin{bmatrix} \vec{a}_B \\ \vec{b}_B \end{bmatrix} \quad \textbf{(S4b)}$$

If the interface had no strain, both sets of base vectors would describe exactly the same supercell, and $X_A = X_B$. If strain is present, instead, and therefore when $X_A \approx X_B$, there must exist a (2×2) strain matrix S that transforms one set into the other:

$$X_A \cdot S = X_A \cdot \begin{bmatrix} s_0 & s_1 \\ s_2 & s_3 \end{bmatrix} = X_B \quad \rightarrow \quad S = X_A^{-1} \cdot X_B \qquad \textbf{(S5)}$$

Knowing S, we can compute the strain ε as the euclidean norm of the difference between the identity matrix I and the strain matrix S:

$$\varepsilon = |I - S|_2 \qquad \textbf{(S6)}$$

One advantage of **Equation S6** is that it is equivalent to **Equation S3** when applied to simple isostructural interfaces like the CdS/CdSe mentioned above, and is therefore comparable with a classical description of strain. We warn the reader that this approach was used in the Main Text to reassess the strain for heterostructures reported in the literature, which might cause small differences between our values and those reported in the publications (if present). For example, Ref. 20 reports 10.7% strain for the InAs/ZnS interface, based on **Equation S2** and with the InAs substrate as reference. We instead indicate 12.0%, based on **Equations S4-6**.

In the *interface generation* and *interface ranking* steps of the algorithm, the user is given a choice on how to distribute the effects of strain between substrate and epilayer. This is described by the *strain_distribution* parameter, which affects the 2D-supercell:

- *strain_distribution = 0* → the film is unchanged, while the epilayer absorbs all strain.
- *strain_distribution = 1* → the epilayer is unchanged, while the film absorbs all strain.
- *strain_distribution = 0.x* → the 2D-supercell is a weighted average of the two above.

This parameter is meant to better represent different situations of epitaxial growth: for example, a thin film grown on a bulk material will likely see the substrate little affected by strain, while the epilayer would be more heavily deformed (*strain_distribution* = 0). Conversely, two chemically similar materials involved in a colloidal interface like that shown in **Figure 4d** of the Main Text will likely share the deformation equally at the interface (*strain_distribution* = 0.5). The user might want to set this parameter depending on their knowledge of the system, for example when interfacing materials with remarkably high or low Young modulus (that is, deformability). In this work, the strain distribution is assumed to be 0.5 for all interfaces.

S3.2. Supercell area threshold

Unlike a high strain, a large supercell area does not necessarily imply an unfavorable interface. Nevertheless, smaller supercells are more likely to result in good atom-to-atom correspondence between the two slabs, and therefore to yield stable interfaces. Indeed, an interface is stable when it maximizes the attractive interactions between the two slabs that are being matched, minimizes the repulsive ones, and leaves no dangling bonds. This usually happens when the two materials being matched feature terminations with ions positioned in similar or complementary patterns, as this will allow to fulfill all the three conditions above. Hence, well-matched materials will likely have similar 2D-periodicities, and the resulting supercell will be small. Conversely, a large 2D-supercell indicates that the two materials feature dissimilar periodicities and possibly terminations, and therefore any bond formed at the interface will repeat identical to itself only after many lattice steps of both materials. Such an interface could still be stable if enough favorable but not equivalent interactions are formed in the space of such repetition, but this circumstance becomes less likely as the supercell area increases.

It is worth clarifying that we are referring here to the "*primitive*" 2D-supercell of an interface, that is the smallest supercell required to fully describe its periodicity. Indeed, an interface can always be described by larger, non-primitive 2D-supercells with no upper limit to their extension (see for example cells a, b, and c in **Figure S8**), but this would not affect the actual atomic structure of the interface, nor its stability. For this reason, the 2D-supercells proposed by our lattice matching algorithm are always primitive.

That said, the choice of a threshold value for the supercell area is somehow arbitrary. By default, the algorithm uses the following cutoff value (**Equation S7** = **Equation 1** in the Main Text):

$$S_T = 2 \cdot \max[S_A(hkl); S_B(h'k'l')] \qquad \textbf{(S7)}$$

where $S_A(hkl)$ and $S_B(h'k'l')$ are the surfaces of the 2D-cells describing the lattice planes being matched at the interface, namely $(hkl)_A$ and $(h'k'l')_B$. The logic is to include 2D-supercells that can be formed by two single-material 2D-cells positioned side-by-side, thus allowing for a more extended repeating unit. An example is seen in **Figure 2c** of the Main Text, where the 2D-supercell of the interface is exactly twice as large as the 2D-cell of the (010) – $Pb_4S_3Br_2$ plane. However, the user is free to tune such threshold depending on their needs.

S3.3. One $(hkl)_A//(h'k'l')_B$ pair, many 2D-supercells

Just defining a pair of lattice planes $(hkl)_A /\!/ (h'k'l')_B$ is insufficient to fully describe the relative orientation of two materials. Indeed, the two structures can still revolve around an axis perpendicular to the interface, potentially resulting in infinite non-equivalent relative orientations. **Figure S8** shows some examples of 2D-supercells all consistent with the $(100) /\!/ (010)$ – $CsPbBr_3/Pb_4S_3Br_2$ label. Of these, panels *a*, *b*, and *c* describe the same interface (*a* = primitive, *b,c* = non-primitive), as seen by the identical strain and relative orientation of the lattice vectors. The interface described in panel *d* is instead different.

The presence of multiple possible interfaces per $(hkl)_A /\!/ (h'k'l')_B$ pair imposes a choice on which interface to optimize. Based on the considerations outlined in **Paragraphs S2.1-S2.2**, we opted for prioritizing the smallest supercell among those with strain below the search threshold. We found that this strategy better reflects the direct correlation *excessive strain = instability*, while assigning a ranking role to the supercell area. In **Table S5** we reported the extended notation $(hkl)_A /\!/ (h'k'l')_B$ + $[hkl]_A \Uparrow [h'k'l']_B$, where the first indices identify the two planes that form the interface, while the second indicates a pair of lattice vectors that are parallel to each other in the plane of the interface, thus removing any rotational ambiguity.

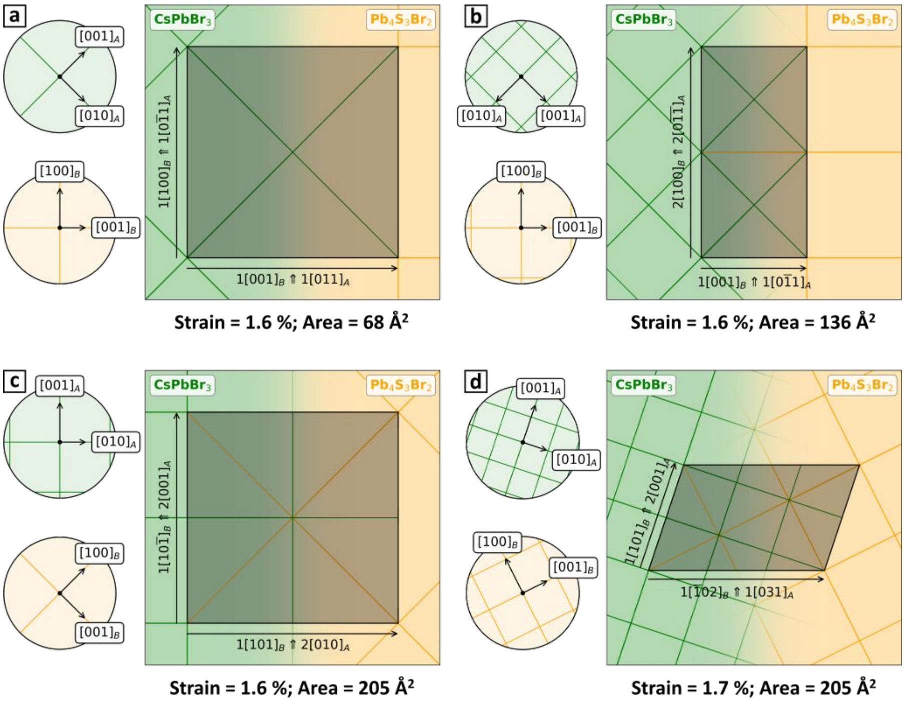

Figure S8. Possible $(100) /\!/ (010)$ – $CsPbBr_3/Pb_4S_3Br_2$ supercells. Supercells in panels (b) and (c) are "*non-primitive*" representations of interface (a), as seen by the identical strain and relative orientation of the two materials (see circular dials). The supercell (d) represents instead a different interface, characterized by a different strain and relative orientation of the two materials. Legend: $CsPbBr_3$ = green lattice and "*A*" label in captions; $Pb_4S_3Br_2$ = orange lattice and "*B*" label in captions. *Lattice matching* settings: strain < 10%; supercell area < 250 $Å^2$.

S4. Other $CsPbBr_3/Pb_4S_3Br_2$ reported interfaces

S4.1. Extended $CsPbBr_3/Pb_4S_3Br_2$ *lattice matching*

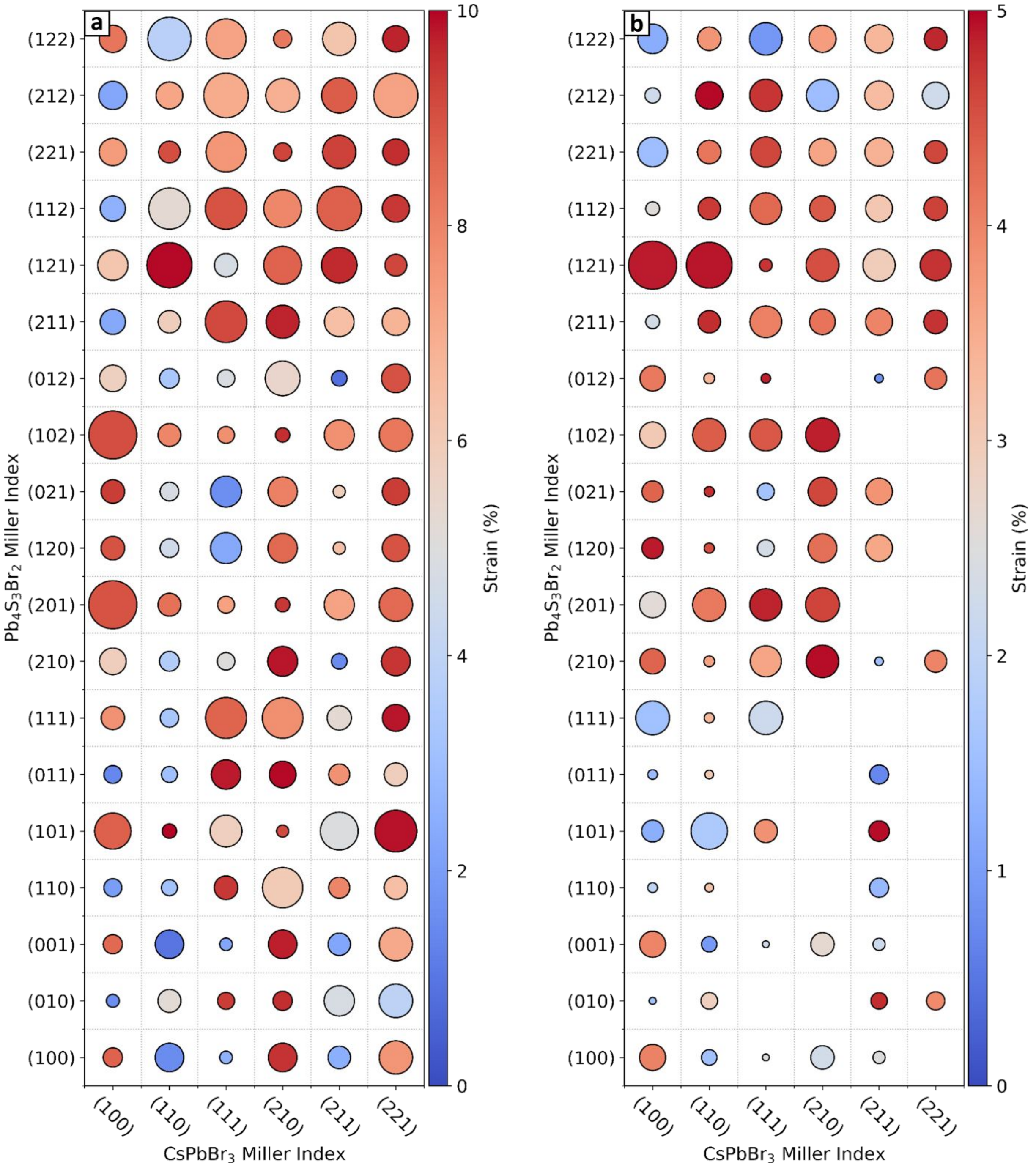

Figure S9. Extended $CsPbBr_3/Pb_4S_3Br_2$ *lattice matching*. a) A more extended version of the *lattice matching* results presented in **Figure 2** of the Main Text, here including Miller indices up to $h,k,l = 2$ for both materials. Constraints: strain < 10 %, area → **Equation S7 = Equation 1** in the Main Text. b) *Lattice matching* results obtained by setting a more limiting strain < 5% threshold. As the number of epitaxial matches increases rapidly with the Miller indices, it is advisable to consider only those lattice planes that can realistically be exposed by the substrate. In colloidal heterostructures this means limiting the planes to the facets of nanocrystal seeds, while in thin films one should focus on the lattice plane exposed by the substrate, if known.

S4.2. (100)⫽(001) – $CsPbBr_3$/$Pb_4S_3Br_2$

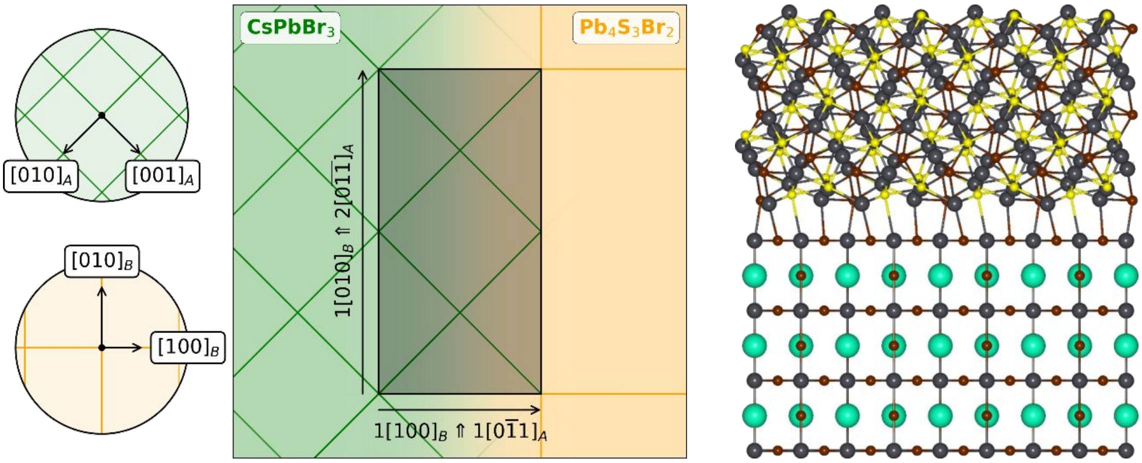

Figure S10. (100)⫽(001) – $CsPbBr_3$/$Pb_4S_3Br_2$ interface. Supercell (left) and most stable interface model (right). The corresponding data are indicated in green in **Table S6**.

Table S6. *Interface ranking* results. Green indicates the most stable interface.

$Pb_4S_3Br_2$ slab index	$CsPbBr_3$ slab index	Interfacial dist. [Å]	$Pb_4S_3Br_2$ charge	$CsPbBr_3$ charge	E_{int} [meV Å^{-2}]
6	0	3.28	0	0	77
6	1	3.57	0	0	77
0	0	2.97	0	0	105
3	0	3.41	+2	0	111
9	1	3.60	-2	0	111
0	1	3.62	0	0	111
3	1	3.67	+2	0	112
9	0	3.55	-2	0	112
10	0	3.17	+2	0	113
10	1	3.56	+2	0	115
8	0	3.28	0	0	116
2	0	3.24	-2	0	116
5	1	3.30	+2	0	117
4	0	3.51	0	0	117
8	1	3.71	0	0	117
4	1	3.91	0	0	117
5	0	3.21	+2	0	117
7	0	3.61	-2	0	118
7	1	4.16	-2	0	119
2	1	4.22	-2	0	119
1	1	3.24	+2	0	131
11	0	3.19	-2	0	133
1	0	3.14	+2	0	133
11	1	3.77	-2	0	135

S4.3. (100)⫽(100) – $CsPbBr_3/Pb_4S_3Br_2$

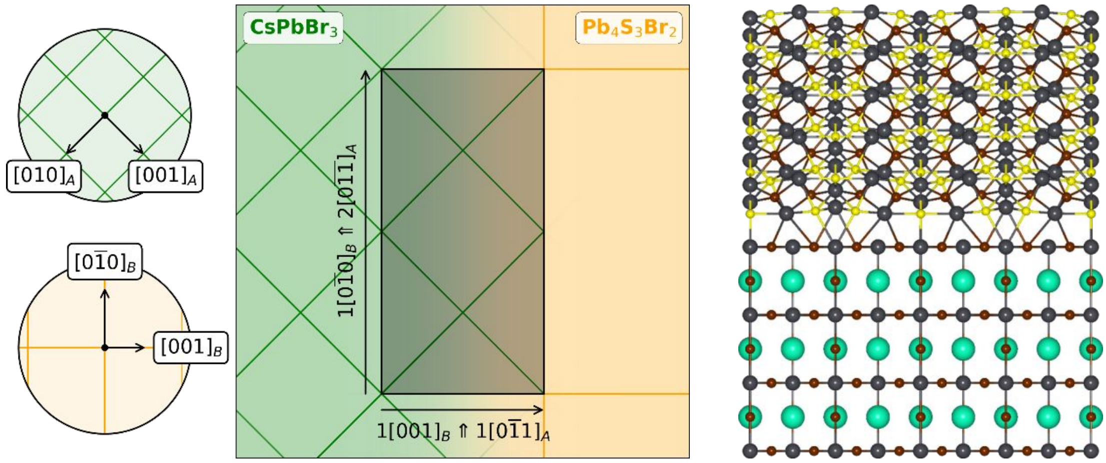

Figure S11. (100)⫽(100) – $CsPbBr_3/Pb_4S_3Br_2$ interface. Supercell (left) and most stable interface model (right). The corresponding data are indicated in green in **Table S7**.

Table S7. *Interface ranking* results. Green indicates the most stable interface.

$Pb_4S_3Br_2$ slab index	$CsPbBr_3$ slab index	Interfacial dist. [Å]	$Pb_4S_3Br_2$ charge	$CsPbBr_3$ charge	E_{int} [meV Å^{-2}]
5	0	3.02	0	0	50
5	1	3.63	0	0	57
2	0	3.26	0	0	65
8	1	3.36	0	0	66
2	1	3.72	0	0	67
8	0	3.62	0	0	68
0	1	3.40	0	0	68
0	0	4.10	0	0	69
1	0	2.59	2	0	71
1	1	3.07	2	0	72
9	1	4.00	-2	0	79
9	0	3.91	-2	0	79
7	1	3.07	2	0	102
7	0	2.88	2	0	104
3	0	3.50	-2	0	106
3	1	3.83	-2	0	106
4	0	3.30	2	0	114
4	1	3.78	2	0	115
6	0	3.21	-2	0	115
6	1	3.46	-2	0	116

Notes: this interface and that in **Paragraph S3.2** have very similar supercells, and would be challenging to tell apart based on the Fourier Transform analysis of HRTEM images.[21] Nevertheless, they lead to different structures due to the diverse orientation of $Pb_4S_3Br_2$. Both are reasonably connected, but the (100)⫽(100) interface shown here appears more favorable by E_{int}, suggesting that this might be the actual orientation of the $Pb_4S_3Br_2$ domain. Unfortunately, no atomic-resolution images of the interface are available to date to confirm this prediction.

S4.4. (110)⫽(001) – $CsPbBr_3$/$Pb_4S_3Br_2$ (low strain)

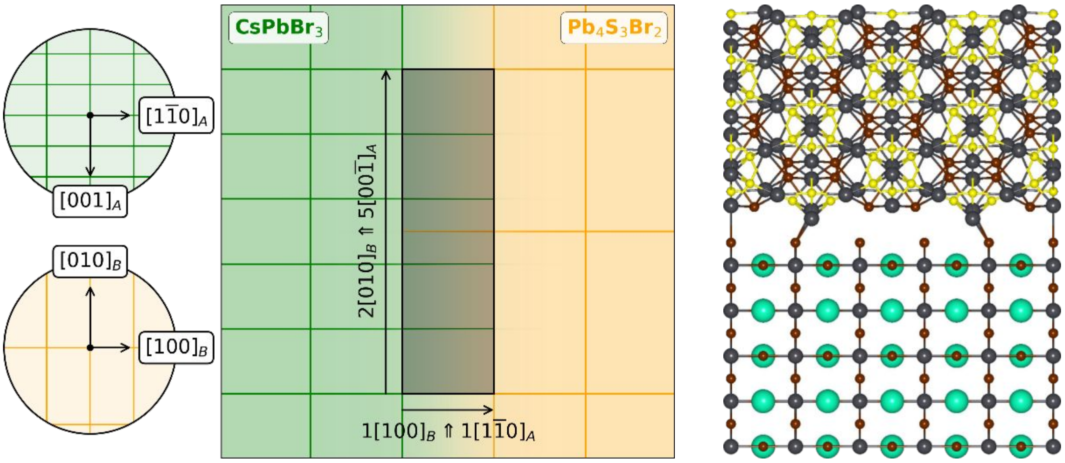

Figure S12. (110)⫽(001) – $CsPbBr_3$/$Pb_4S_3Br_2$ interface. Supercell (left) and most stable interface model (right). The corresponding data are indicated in green in **Table S8**. The strain parameter for this interface model is 0.94%.

Table S8. *Interface ranking* results. Green indicates the most stable interface.

$Pb_4S_3Br_2$ slab index	$CsPbBr_3$ slab index	Interfacial dist. [Å]	$Pb_4S_3Br_2$ charge	$CsPbBr_3$ charge	E_{int} [meV Å^{-2}]
5	1	2.20	+2	-1	102
6	1	3.54	0	-1	107
9	0	2.97	-2	+1	109
3	1	2.74	+2	-1	110
6	0	4.22	0	+1	115
10	1	2.78	+2	-1	116
2	0	3.07	-2	+1	117
7	0	3.17	-2	+1	125
1	1	2.68	+2	-1	131
11	0	2.97	-2	+1	140
8	1	3.87	0	-1	149
4	0	4.22	0	+1	149
0	0	4.22	0	+1	150
0	1	4.22	0	-1	150
8	0	4.22	0	+1	150
4	1	4.22	0	-1	150

Notes: the large number of dangling bonds visible in **Figure S12** make this interface unlikely to form experimentally. However, there appears to be a pattern of alternated areas of good and poor atom-to-atom matching, which suggests that a more strained but better connected model might exist. Indeed, by increasing the strain threshold to < 15% we could identify a smaller 2D-supercell which resulted in a more stable interface model (see **Paragraphs S3.5-6**).

S4.5. (110)⫽(001) – $CsPbBr_3$/$Pb_4S_3Br_2$ (high strain)

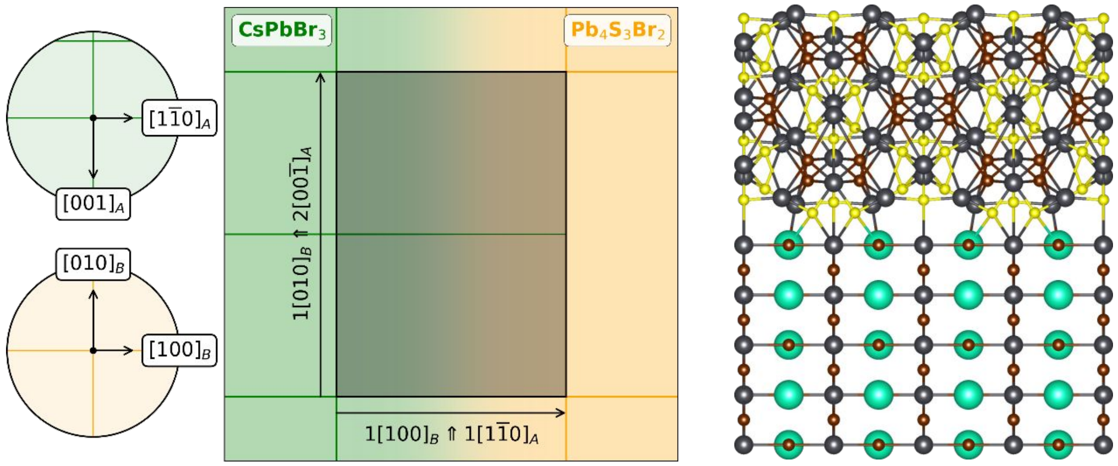

Figure S13. (110)⫽(001) – $CsPbBr_3$/$Pb_4S_3Br_2$ interface. Supercell (left) and most stable interface model (right). The corresponding data are indicated in green in **Table S9**. The strain parameter for this interface model is 14.7%.

Table S9. ***Surface ranking*** **results**. Green indicates the most stable interface.

$Pb_4S_3Br_2$ slab index	$CsPbBr_3$ slab index	Interfacial dist. [Å]	$Pb_4S_3Br_2$ charge	$CsPbBr_3$ charge	E_{int} [meV Å^{-2}]
9	0	2.36	-2	+1	68
10	1	2.32	+2	-1	68
5	1	2.40	+2	-1	84
11	0	2.34	-2	+1	90
3	1	2.56	+2	-1	91
6	1	3.28	0	-1	100
2	0	2.89	-2	+1	103
6	0	3.28	0	+1	110
7	0	2.86	-2	+1	119
0	0	2.92	0	+1	119
1	1	2.59	+2	-1	124
0	1	2.64	0	-1	136
4	0	3.10	0	+1	140
4	1	2.91	0	-1	143
8	1	2.62	0	-1	143
8	0	2.89	0	+1	144

Notes: despite being better connected that the model shown in **Paragraph S3.4**, this interface model requires a much higher strain. As this interface was reported in nano-heterostructures[21] where the contact surface between the two domains was just ~20×20 nm^2, it is possible that such level of strain might be tolerable, especially considering the intrinsic softness of metal halides.[22,23] This considered, we deem the reported interface plausible, albeit perhaps not particularly favorable. However, we cannot exclude a case of misidentification, as the orientation of the two domains was assigned based on non-atomic-resolution TEM images.

S4.6. (110)⫽(100) – $CsPbBr_3/Pb_4S_3Br_2$ (high strain)

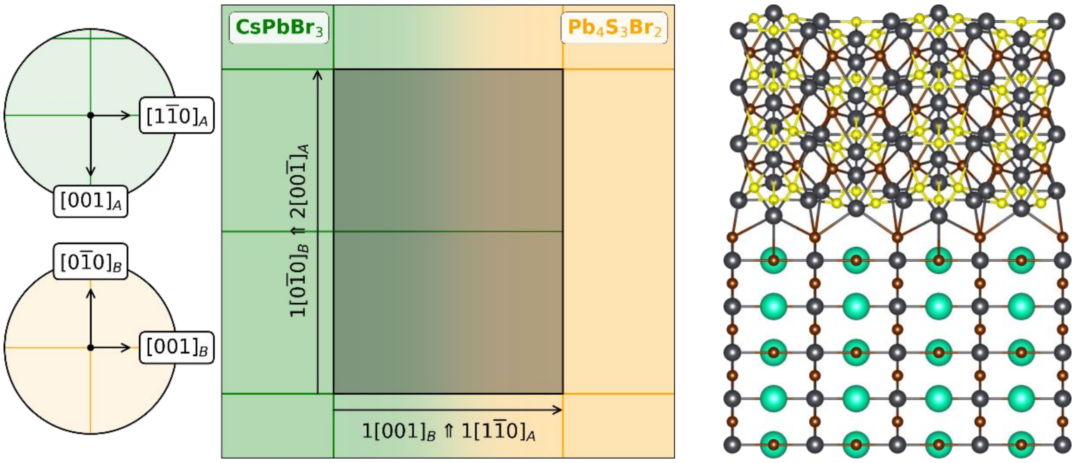

Figure S14. (110)⫽(100) – $CsPbBr_3/Pb_4S_3Br_2$ interface. Supercell (left) and most stable interface model (right). The corresponding data are indicated in green in **Table S10**. The strain parameter for this interface model is 14.7%.

Table S10. ***Surface ranking*** **results**. Green indicates the most stable interface.

$Pb_4S_3Br_2$ slab index	$CsPbBr_3$ slab index	Interfacial dist. [Å]	$Pb_4S_3Br_2$ charge	$CsPbBr_3$ charge	E_{int} [meV Å^{-2}]
1	1	1.73	+2	-1	48
9	0	2.80	-2	+1	64
5	0	2.98	0	+1	66
3	0	2.71	-2	+1	69
7	1	2.29	+2	-1	72
4	1	2.32	+2	-1	72
5	1	2.97	0	-1	85
2	1	2.52	0	-1	86
8	1	2.80	0	-1	89
2	0	2.72	0	+1	91
0	0	2.97	0	+1	91
8	0	2.77	0	+1	91
6	0	2.75	-2	+1	100
0	1	4.22	0	-1	104

Notes: this interface and that in **Paragraph S3.5** have very similar supercells, and would be challenging to tell apart based on the Fourier Transform analysis of HRTEM images.[21] Nevertheless, they lead to different structures due to the diverse orientation of $Pb_4S_3Br_2$. Both are reasonably connected, but the (110)⫽(100) interface shown here appears more favorable by E_{int}, suggesting that this might be the actual orientation of the $Pb_4S_3Br_2$ domain. Unfortunately, no atomic-resolution images of the interface are available to date to confirm this prediction.

S5. Interface generation

S5.1. Generation of non-equivalent slabs

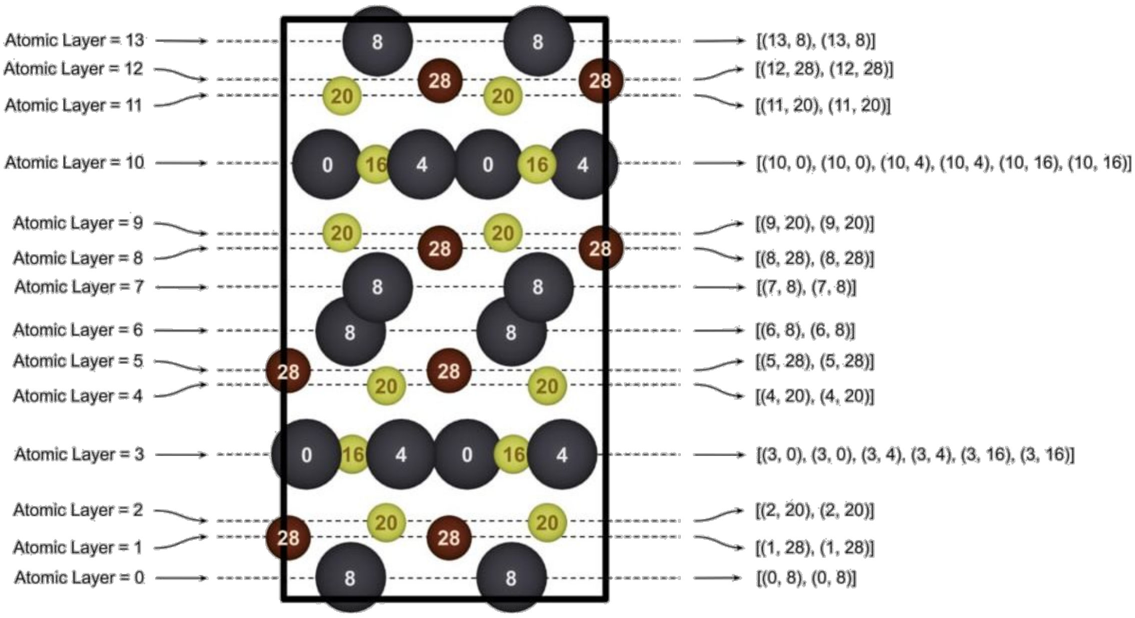

Figure S15. Generating non-equivalent (010) slabs for $Pb_4S_3Br_2$. Slabs are generated by cleaving the bulk structure of a material parallel to the interface contact plane. Unless clustering is applied (see **Paragraph S4.2**), the bulk is sliced at every atomic layer (left). To check if two of the resulting slabs are equivalent by symmetry, the algorithm compares the content of all atomic layers at and above the interface (i.e., moving away from the slab surface). If these are composed of atoms that are equivalent by symmetry and the layers alternate in the same exact sequence (right), then the two slabs are considered equivalent.

Given a material and a [*hkl*] lattice direction, a comprehensive set of slabs is generated by slicing the bulk structure along (*hkl*) planes positioned to cut at each atomic layer. Layers are identified as slabs of atoms with identical or similar vertical coordinates: for example, layer 3 in **Figure S15** contains 4 Pb^{2+} ions (grey) and 2 S^{2-} ions (yellow). When atoms are not lying exactly flat on the same plane, a tolerance threshold is applied: the motivations and clustering method are discussed in **Paragraph 4.2**, with the *P*nma structure of $CsPbBr_3$ as an example.

Once all slabs have been generated, the algorithm checks if any of them are equivalent by symmetry, meaning that they would produce identical interfaces if matched with the same slab of another material. To do so, the algorithm labels each atom in the slab based on the position they occupy in the bulk structure of the parent material using the Python package *spglib:*[24] atoms equivalent by symmetry (i.e., that are mapped one onto another by the space group symmetry operations), are given the same label. This allows to express the composition of each atomic layer in terms of equivalent atoms.

However, due to the directionality of the epitaxial growth, comparing only the composition of the surface layer is not enough to check if two slabs are equivalent. For example, layers 6 and 7 in **Figure S15** are not, despite being both terminated by equivalent Pb^{2+} ions, because choosing one or the other slab would result in a different number of Pb^{2+} ions at the interface. Instead, the algorithm checks all the atomic layers at and above the slab surface: if they are identical by composition and sequence (e.g., layers 3 and 10), then the two slabs are equivalent. For instance, the $Pb_4S_3Br_2$ structure shown in **Figure S15** features a 2_1 screw axis along the [010] direction, which makes its 14 possible terminations equivalent two-by-two. Hence, we can consider just 7 non-equivalent slabs, obtained by cleaving the structure at layers 0-6.

S5.2. Atom clustering algorithm

Unless high-symmetry structures are considered, atoms seldom lie perfectly flat on the same plane. Therefore, if we were to slice a bulk material in correspondence of every ion, the number of possible terminations would grow rapidly with its complexity. For example, slicing the *P*nma orthorhombic structure of $CsPbBr_3$ would result in either *8* (**Figure S16b**) or *10* different slabs (**Figure S16c**), depending on the direction of the cut. However, a comparison with the *P*m-3m cubic structure (**Figure S16a**) quickly reveals that most of these slabs would be meaningless for the purpose of generating epitaxial interfaces, as they would feature highly defective surface terminations with systematically missing atoms (see for example **Figure S16g-i**, where the missing Br^- ions are highlighted in red).

Therefore, we introduced a clustering algorithm that regroups ions with similar distance into the same atomic layer. By default, atoms are part of the same layer if they are found within 0.15 × [max vertical distance between consecutive atoms]. For example, atoms in **Figure S16b** have a maximum vertical distance of 2.65 Å along the [010] direction (i.e., Br-to-Cs vertical distance, magenta arrow), and hence the grouping tolerance is 0.15 × 2.65 Å = 0.40 Å. As a result, the clustering algorithm could ensure that the $PbBr_2$ and CsBr atomic slabs were preserved as solid entities upon slicing, reducing the number of slabs to just 4 (two $PbBr_2$-terminated and two CsBr-terminated). Subsequently, the symmetry equivalency check described in **Paragraph 4.1** further reduced this number to two, effectively identifying the same slabs found for the cubic *P*m-3m $CsPbBr_3$ structure (**Figure S7d-f**). See **Section S1** for the results of *lattice matching* and *interface optimization* when considering the orthorhombic *P*nma structure for $CsPbBr_3$.

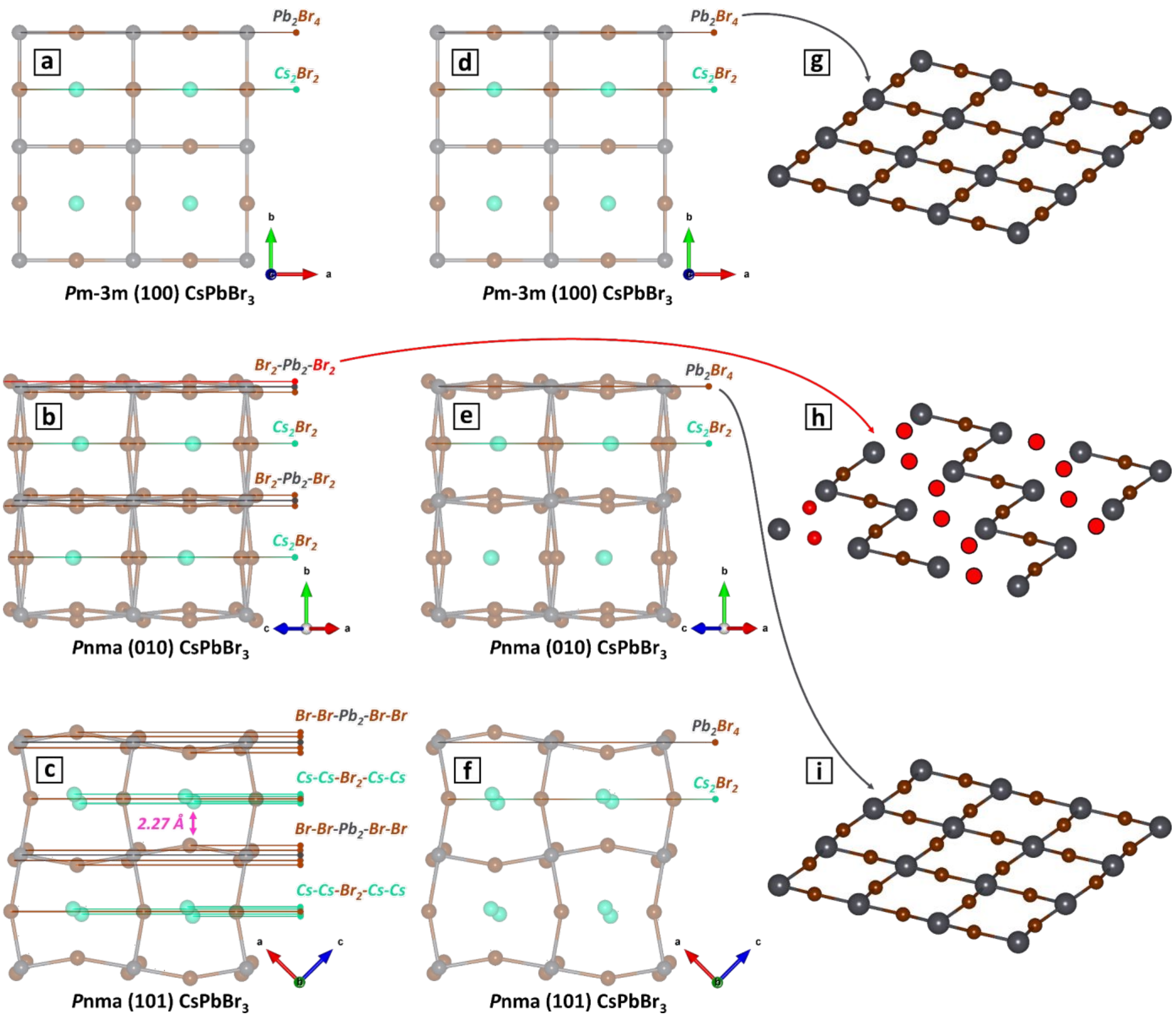

Figure S16. Effects of atom clustering on *P*nma $CsPbBr_3$. a-c) Surface terminations identified in the absence of atom clustering and equivalency check for a) *P*m-3m $CsPbBr_3$ along the [100] direction, b) *P*nma $CsPbBr_3$ in the [010] orientation, and c) *P*nma $CsPbBr_3$ in the [101] orientation. d-e) Surface terminations identified for the same structures when both atom clustering and equivalency check are implemented. g-i) Surface terminations of slabs resulting from the cuts highlighted. Noticeably, the surface shown in panel (h) has systematically missing Br^- ions, and would therefore result in a highly defective model if used to assemble an epitaxial interface. For this reason, it is excluded by our atom clustering algorithm.

S5.3. On the slabs surface charge *Q*

To assign a surface charge to each of the two slabs forming an interface, we must consider that:

1. Each slab should emulate the behavior of a semi-infinite solid, as real-world interfaces can extend for very long distances away from the interface plane (>> 1 unit cell).

2. Therefore, all slabs must be overall neutral to avoid unintended electrostatic interactions due to a non-zero total charge. This is ensured by constructing slabs whose thickness is a multiple of the (*hkl*) plane periodicity, which forces them to contain an integer multiple of the material's formula unit (e.g., 16 × $Pb_4S_3Br_2$). This also allows to compare the relative energy of different slabs, as the total composition is constant.

3. The surface charge has meaning and effect only locally, close to the interface, and not on average on the entire slab structure.

Given these premises, the surface charge attributed to a slab should consider both the charges of ions and their position. Ions closer to the slab surface should exert a stronger influence, while those farther away should gradually diminish their impact, as their contribution is compensated by the infinite bulk located behind them. This can be captured by constructing an *"effective surface dipole" P*, where the charges of ions are weighted over their distance from the interface:

$$P = \sum_i q_i \cdot (D - d_i) \quad \textbf{(S8)}$$

where i iterates over all ions in the slab, q_i is the ith ion's charge as automatically assigned by PyMatGen,[25] d_i is the ion's distance from the slab's surface, and D is the slab thickness. By construction, D is a multiple of $d_{(hkl)}$, that is the periodicity of the material perpendicular to the (*hkl*) interface. Note that the base quantity of P is that of an *electric dipole = charge · length*. Therefore, we can retrieve an "*effective surface charge" Q* by dividing P by its length D:

$$Q = \frac{P}{D} = \frac{\sum_i q_i \cdot (D - d_i)}{D} = \sum_i q_i - \frac{\sum_i q_i \cdot d_i}{D} = -\frac{\sum_i q_i \cdot d_i}{D} \quad \textbf{(S9)}$$

which yields **Equation S9** = **Equation 2** in the Main Text. Note that $\sum_i q_i = 0$ because each slab is neutral by construction. This is also why the slab's thickness must be an integer multiple of $d_{(hkl)}$, as this ensures that the slab contains a multiple of the material's formula unit.

Conceptually, two slabs with surface charges *Q* and *Q'* will, in the far-field approximation (i.e., where slabs are so distant that only electrostatic interactions matter), repel or attract each other as if they were two point charges *Q* and *Q'* located at the slab's surfaces. This is why comparing the signs of *Q* and *Q'* is a convenient screening tool to exclude electrostatically unfavorable interfaces. Likewise, it intuitively explains why [0/0], [0/+], and [0/-] interfaces cannot be excluded: their far-field electrostatic interaction is null, but when put in close proximity the formation of bonds or the effect of other attractive interactions (e.g., van der Waals) might stabilize them. Finally, it also accounts for [+/-] interfaces that are not charge-balanced: the two slabs will indeed attract each other, and if the system has a way to accommodate that local charge accumulation (e.g., by introducing defects or locally altering the oxidation state of species), a stable interface might form.

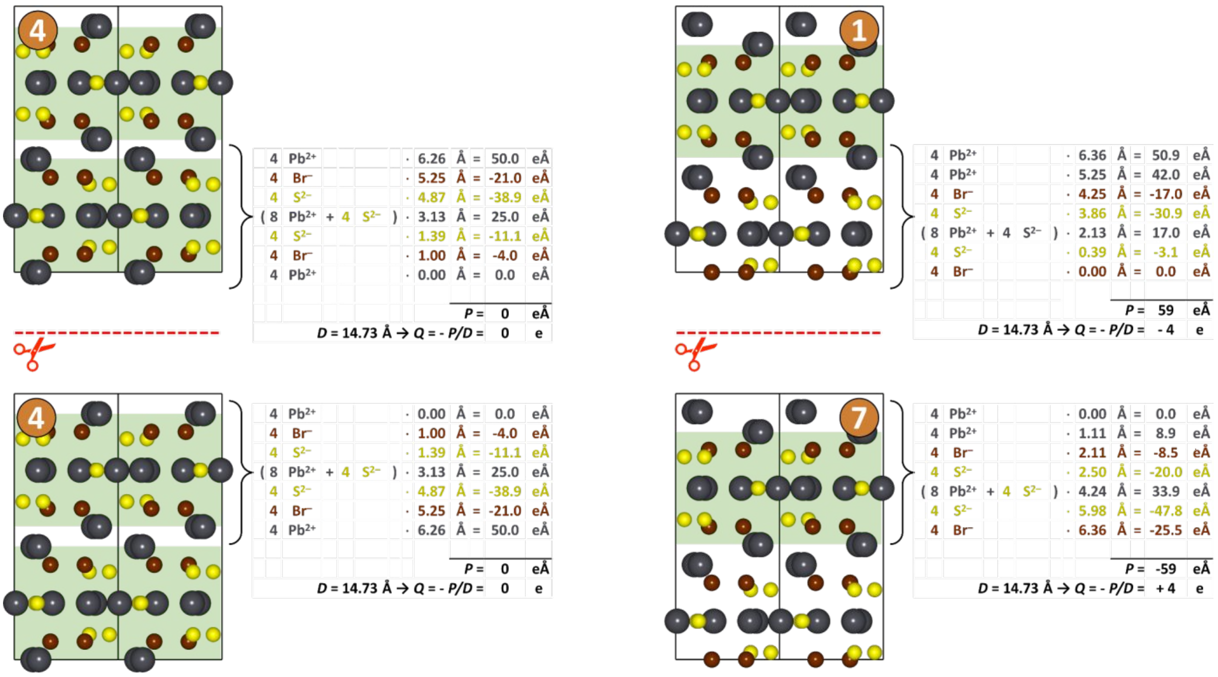

Figure S17. Calculation of surface charges demonstrated on (010) slabs of $Pb_4S_3Br_2$. Two (010)⫽(010) – $Pb_4S_3Br_2$/$Pb_4S_3Br_2$ homo-interfaces are here obtained by cleaving the bulk at different planes (dashed red lines). The left interface is of the [0/0] type, while the right one is of the [+/-] type. Note that in the latter case the charges of the two slabs have opposite sign and equal magnitude, as expected when slicing a neutral bulk structure (see tables on the side, which represent the application of **Equation S9**). The green shading identifies slabs that are overall neutral. In the case of #7, this helps visually identify the source of the residual + charge in the extra layer of Pb^{2+} cations on the top. All labels are consistent with **Figure 3** of the Main Text.

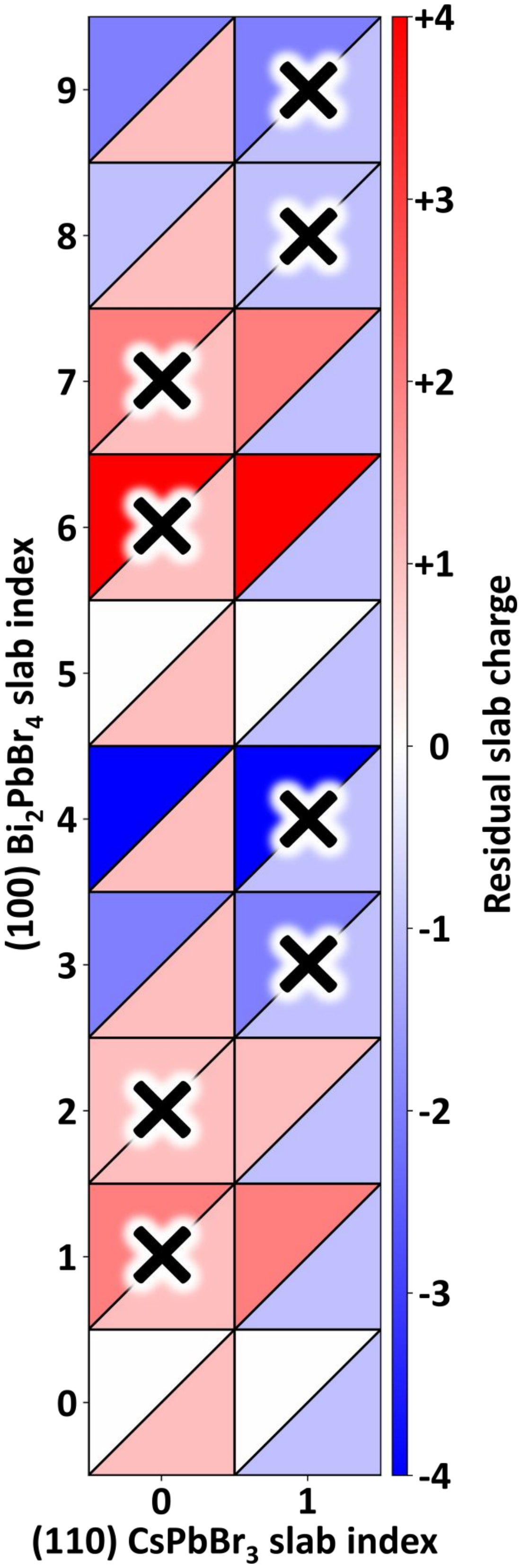

Figure S18. Example of charge balance screening. Results of charge balance screening for the (110)⫽(100) – $CsPbBr_3/Bi_2PbS_4$ interface discussed in **Section 2.3** of the Main Text and in **Section S7** of the SI. Each tile represents a pair of slabs, identified by a numerical index. The pairs marked with an × are excluded from the *surface matching and ranking* step due to their repulsive [+/+] or [–/–] nature, thus reducing the number of interface models from 20 to 12.

S6. Surface Matching and Ranking

S6.1 Visual representation of E_{int} and E_{adh}

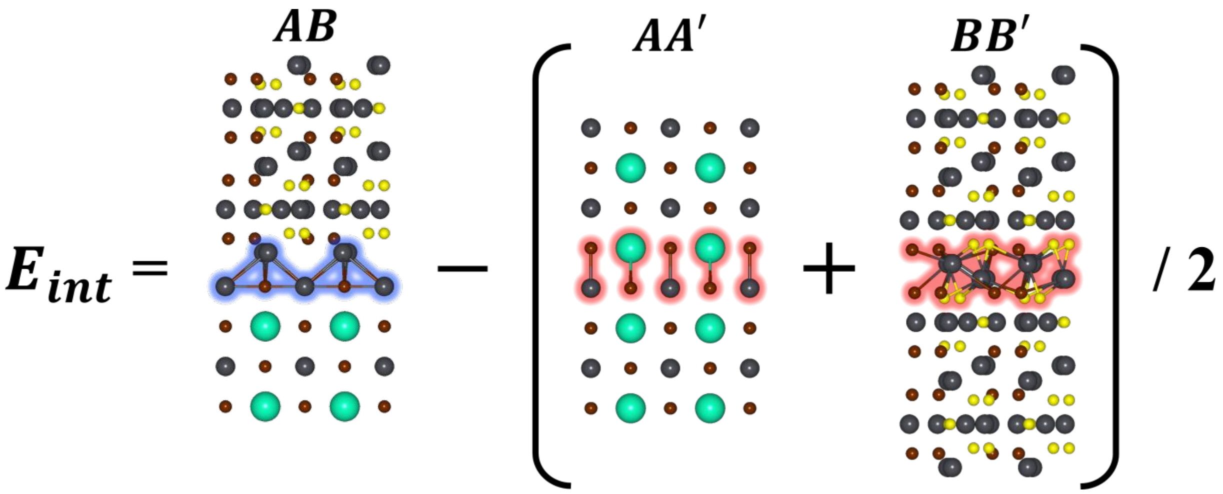

Figure S19. Interface energy visualized for the (010)⫽(010) – $CsPbBr_3/Pb_4S_3Br_2$ interface. E_{int} compares the energy of the interface with that of the two bulk materials. In essence, it indicates how (un)favorable it is to cleave the bonds of the bulk (highlighted in red) and in exchange form those at the interface (highlighted in blue). The bulk terms are divided by two to ensure that the total energy of bonding interactions is compared over the same interface area.

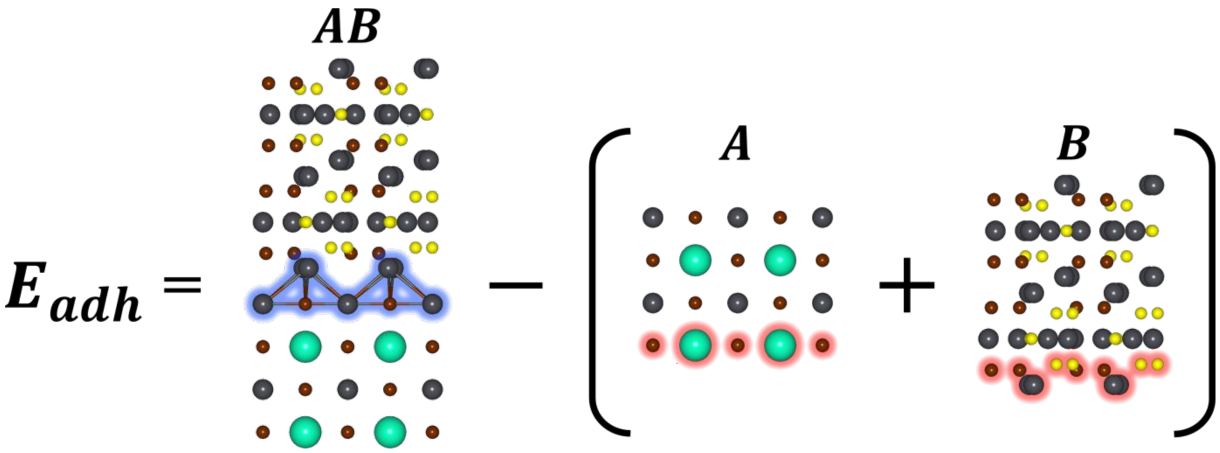

Figure S20. Adhesion energy visualized for the (010)⫽(010) – $CsPbBr_3/Pb_4S_3Br_2$ interface. E_{adh} compares the energy of the interface with that of the two isolated slabs. In essence, it indicates how favorable it is for the two unterminated slab surfaces (highlighted in red) to bond together and form the interface (highlighted in blue). E_{adh} is positive when the interactions between slabs are repulsive, and vice versa.

S6.2 Adhesion energy maps

S6.2.1. Constructing E_{adh} maps

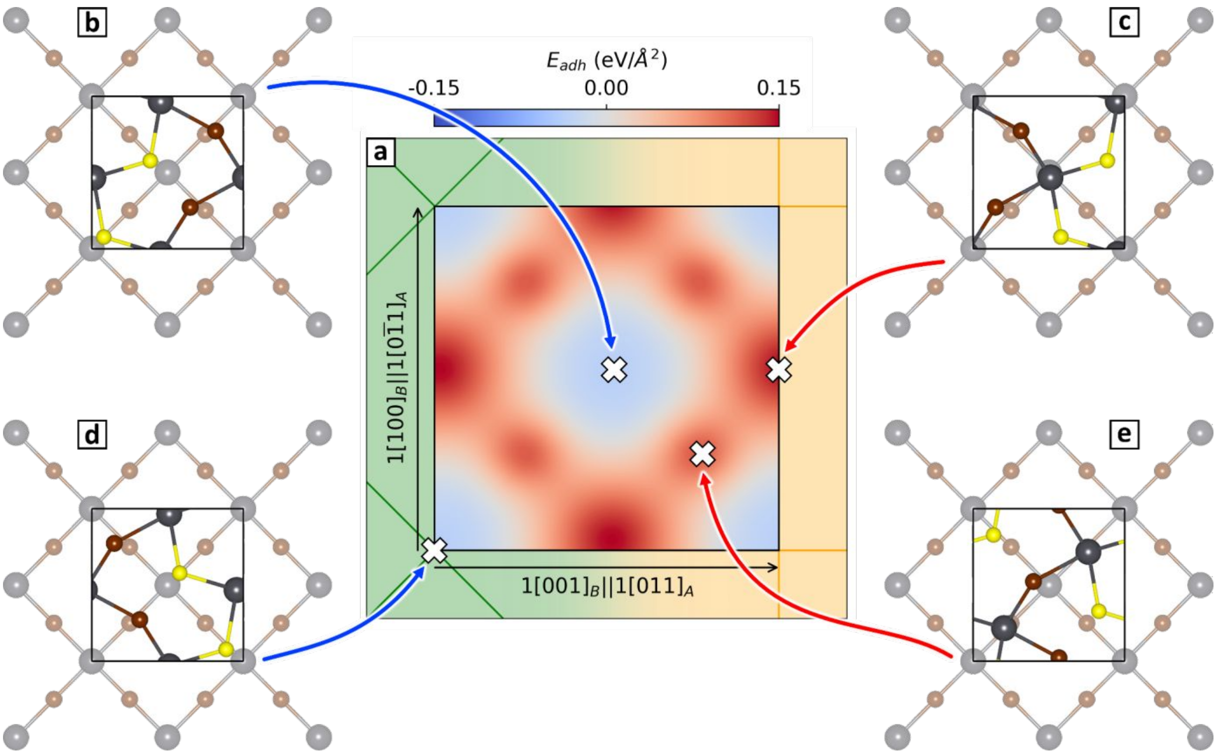

Figure S21. Adhesion energy map for (010)⫽(010) – $Pb_4S_3Br_2$/$Pb_4S_3Br_2$. a) The E_{adh} map is shown overlaid to the 2D-supercell of the corresponding interface (see **Figure 2b** of the Main Text). The × symbols mark high-symmetry positions in the supercell, for which a top-down view of the interface is shown in panels (b-e).

To ensure an efficient optimization of the interface, Ogre employs a particle swarm algorithm[26,27] to find the energy minimum in the 3D-space formed by the *xy*-lateral shift and *z*-distance of the two slabs. Hence, both the epitaxial registry and the interfacial distance are optimized at the same time, without the need to explicitly construct E_{adh} maps.

However, E_{adh} maps can be useful to visually assess the quality of interface models (see **Section S5.2.2**). These are constructed by pinning one slab (material *A*), and shifting the other laterally (material *B*) to explore all the possible epitaxial registries (i.e., lateral shifts), while keeping the interfacial distance fixed at the value identified by the particle swarm optimization. **Figure S21** shows an example of E_{adh} map and the corresponding epitaxial registries. We note that constructing energy maps based on E_{adh} and E_{int} would be equivalent, as these energies differ only by a rigid shift.

S5.2.2. Energy *vs* interfacial distance curves and non-bonding interfaces

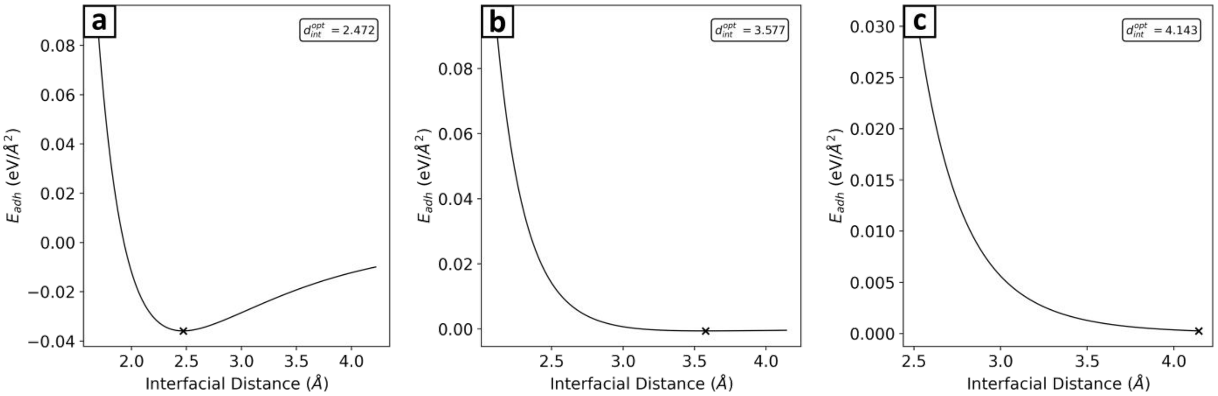

Figure S22. Examples of E_{adh} *vs* interfacial distance curves. Three typical examples of energy *vs* interfacial distance curve profiles. From left to right: a) well-defined energy minimum (b) shallow energy minimum; c) no energy minimum, indicating a "*non-bonding*" interface.

The E_{adh} *vs* interfacial distance curves can be a useful tool to interpret the results of the simulation. By default, Ogre computes such curves up to 1.2 × the maximum sum of ionic radii across all ions in materials *A* and *B*. This approach ensures that the longest possible bonding distance between the slabs always falls within the calculation range.

Normally, stable and highly favorable interface models exhibit a curve like the one shown in **Figure S22a** (from the (100)⫽(010) – $CsPbBr_3/Pb_4S_3Br_2$ interface, see **Figure 4** in the Main Text), which displays a well-defined energy minimum. In other cases, the energy minimum might appear shallow like in **Figure S22b** (from the (100)⫽(210) – $CsPbCl_3/Pb_3S_2Cl_2$ interface, see **Figure 6** in the Main Text). This might be an indication of a problematic interface, which should prompt a further, visual scrutiny of the atomistic model, and in general suggests that the calculated interfacial distance might become less accurate. Finaly, for some interface models the electrostatic term of our classical potential might be simply insufficient to counter the Born repulsive term within the given interfacial distance range, as shown in **Figure S22c** (from the (100)⫽(110) – $CsPbCl_3/Pb_3S_2Cl_2$ interface, see **Figure 6** in the Main Text). Those interfaces are considered "*non-bonding*", and should be discarded. In the Ogre output files, models of this kind are flagged as "*converged = False*".

S6.3 Parametrized classical potential

To achieve a fast prediction of interfaces between polar materials, we implemented in Ogre a pair-wise interatomic potential consisting of the sum of an electrostatic term, which can be repulsive or attractive, and a Born term, that is always repulsive. Traditionally, the electrostatic potential of fully periodic systems is calculated through the Ewald summation, of which many optimized open-source Python implementations are available for 3D-periodic structures (e.g., PyMatGen,[25] SchNetPack,[28,29] and DScribe[30,31]). However, many of the computational strategies employed to speed up the 3D Ewald summation are not valid for 2D-periodic systems like epitaxial interfaces, and there are currently no Python-based Ewald sum implementations optimized specifically for 2D-periodic models.[32] Therefore, we implemented instead a non-Ewald algorithm known as *"damped shifted force"* (DSF), which was introduced by Fennell and Gezelter.[33] This potential has the advantage of producing results comparable to the Ewald summation without imposing any requirements on the periodicity of the system, and most importantly its complexity scales linearly as $O(N)$, while the most efficient Ewald summation implementations scale as $O(N \cdot \log(N))$.

The DSF potential is defined in **Equation S10**, where q_i and q_j are the charges of ions i and j, and d_{ij} is the distance between them. Here, R_c is the cutoff radius of the potential, while α is a dampening parameter used to accelerate the convergence of the sum.

$$V_{Coulomb}^{DSF} = q_i q_j \left(\frac{erfc(\alpha d_{ij})}{d_{ij}} - \frac{erfc(\alpha R_c)}{R_c} + \left(\frac{erfc(\alpha R_c)}{R_c^2} + \frac{2\alpha}{\sqrt{\pi}} \frac{e^{-\alpha^2 R_c^2}}{R_c} \right) \left(d_{ij} - R_c \right) \right) \quad \forall d_{ij} \leq R_c \qquad \textbf{(S10)}$$

Since the Coulomb potential is monotonic in nature, the optimal bonding distance between ions of opposite charge would be zero in the absence of repulsive contributions. Hence, to ensure a non-zero optimal bonding distance we included a purely repulsive Born term into the potential, here described by **Equation S11**:

$$V_{Born} = \frac{B_{ij}(d_{0,ij})}{d_{ij}^n} \qquad \textbf{(S11)}$$

Here, $B_{ij}(d_{0,ij})$ is a structure-dependent constant chosen to reproduce the optimal bonding distance $d_{0,ij}$ between a given pair of ions (see **Equations S13-16** below), while n controls how sharp the energy minimum will be. In this work n is set to 12, which we found to produce good results in homo-interfaces (i.e., one material cleaved and reassembled) simulated for testing.

Equation S12 now defines the total electrostatic + Born interatomic pairwise potential, where $B_{ij}(d_{0,ij})$ is the only system-dependent parameter to be determined:

$$V_{Total} = V_{Coulomb}^{DSF} + V_{Born} \tag{S12}$$

To optimize $B_{ij}(d_{0,ij})$ for a given interface, we can find the $B_{ij}(d_{0,ij})$ value for which the derivative of the total potential with respect to the ion-ion distance d_{ij} is zero at the optimal bonding distance (i.e., $d_{0,ij}$), assuming that the two ions have opposite signs:

$$0 = \frac{d}{d(d_{ij})}(V_{Total})\Big|_{d_{ij}=d_{0,ij}} = \frac{d}{d(d_{ij})}\left(V_{Coulomb}^{DSF} + V_{Born}\right)\Big|_{d_{ij}=d_{0,ij}} \tag{S13}$$

$$0 = \frac{d}{d(d_{ij})}\left[-|q_i||q_j|\left(\frac{erfc\left(\alpha d_{ij}\right)}{d_{ij}} - \frac{erfc(\alpha R_c)}{R_c} + \left(\frac{erfc(\alpha R_c)}{R_c^2} + \frac{2\alpha}{\sqrt{\pi}}\frac{e^{-\alpha^2 R_c^2}}{R_c}\right)\left(d_{ij} - R_c\right)\right) + \frac{B_{ij}\left(d_{0,ij}\right)}{d_{ij}^n}\right]_{d_{ij}=d_{0,ij}} \tag{S14}$$

$$0 = \left[-|q_i||q_j|\left(-\frac{erfc\left(\alpha d_{ij}\right)}{d_{ij}^2} - \frac{2\alpha}{\sqrt{\pi}}\frac{e^{-\alpha^2 d_{ij}^2}}{d_{ij}} + \frac{erfc(\alpha R_c)}{R_c^2} + \frac{2\alpha}{\sqrt{\pi}}\frac{e^{-\alpha^2 R_c^2}}{R_c}\right) - n\frac{B_{ij}\left(d_{0,ij}\right)}{d_{ij}^{n+1}}\right]_{d_{ij}=d_{0,ij}} \tag{S15}$$

$$B_{ij}\left(d_{0,ij}\right) = -|q_i||q_j|\frac{d_{ij}^{n+1}}{n}\left[-\frac{erfc\left(\alpha d_{0,ij}\right)}{d_{ij}^2} - \frac{2\alpha}{\sqrt{\pi}}\frac{e^{-\alpha^2 d_{0,ij}^2}}{R_c} + \frac{erfc(\alpha R_c)}{R_c^2} + \frac{2\alpha}{\sqrt{\pi}}\frac{e^{-\alpha^2 R_c^2}}{R_c}\right] \tag{S16}$$

To determine the optimal bonding distance between each pair of ions we assume that the two input bulk structures are stable, and have equilibrium lattice constants and ion coordinates. Then, we use the CrystalNN nearest-neighbor algorithm implemented in PyMatGen[25,34] to find the coordination number of each site and the minimum bonding distance (i.e., $d_{0,ij}$) between each pair of symmetrically unique sites of both bulk structures. Once $d_{0,ij}$ and the coordination numbers of the i and j ions have been determined, $d_{0,ij}$ can be decomposed into the individual ionic radii of the anion (r^-) and cation (r^+) using the ratio between the ionic radii tabulated by Shannon and Baloch et. al.[35,36]

Once r^+ or r^- are assigned to each ion, the optimal bonding distance for ion pairs across the interface can be predicted simply by summing the two individual ionic radii (i.e., $d_{0,ij} = r^+ + r^-$). Hence, $B_{ij}(d_{0,ij})$ can be determined through **Equation S15**, and the pairwise potential between i and j is finally described as per **Equations S10-11**. Crucially, this allows to reliably predict the potential and optimal bond length also for pairs of ions that are not found in the two individual bulk structures (e.g., Cs-S for the $CsPbBr_3/Pb_4S_3Br_2$ interface models shown in **Figure 4c** of the Main Text). The workflow for decomposing the equilibrium distance $d_{0,ij}$ into individual radii is visually described in **Figure S23**.

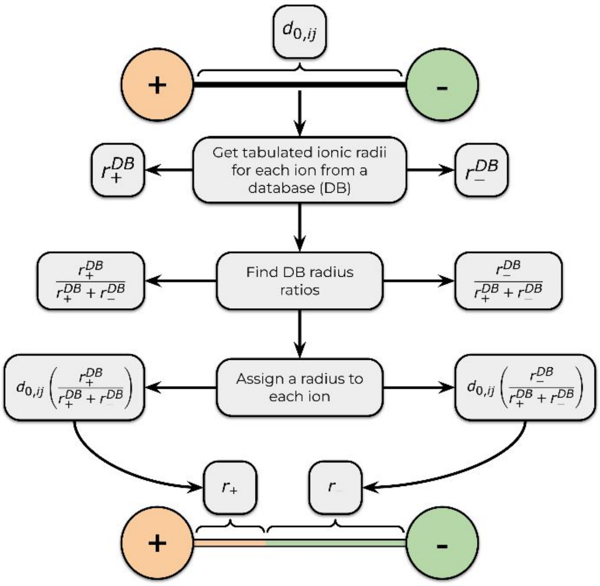

Figure S23. Decomposing $d_{0,ij}$ into $r^+ + r^-$ for a given pair of ions. The equilibrium distance between two ions *i* and *j* (i.e., $d_{0,ij}$) is extracted from the input bulk structures, together with the coordination environments of the same ions. Then, the ionic radii for all the elements and coordination environments are extracted from databases, and used to calculate the fractional contribution of each ion to the bond length $d_{0,ij}$. Based on such fractional contributions, $d_{0,ij}$ is finally decomposed as $d_{0,ij} = r^+ + r^-$, which are then used to estimate the optimal bond lengths for ion pairs that are absent in the two input bulk structures (i.e., at the interface).

S7. Electrostatic potential vs DFT

To compare the performances of our electrostatic potential with DFT, we repeated the *surface matching and ranking* procedure for the (100)⫽(010) – $CsPbBr_3/Pb_4S_3Br_2$ interface with the SCAN+rVV10 functional, which is known to produce reliable results for metal halide perovskites.[37,38] Given the large number of atoms in the model, a full 3D-optimization of the interface geometry would have exceeding computational costs. Therefore, we trusted the epitaxial registry identified by the electrostatic potential, as the *xy*-position of the energy minimum is generally easy to identify thanks to the geometric arrangement of atoms at the interface. Conversely, the interfacial distance was re-optimized independently by DFT, and we also computed the E_{adh} maps for the three top-ranking interfaces to visually compare how Ogre and DFT perform far from the energy minimum (see **Figure 5b-c** of the main text). **Table S11** provides detailed results, including the E_{int} values plotted in **Figure 5a** of the Main Text.

Table S11. (100)⫽(010) – $CsPbBr_3/Pb_4S_3Br_2$, electrostatic potential *vs* DFT. Interface models are listed as ranked by the Ogre electrostatic potential. Green indicates the most stable interface. Data referring to the Ogre potential are identical to those in **Table S2**.

$Pb_4S_3Br_2$ frag.	$CsPbBr_3$ frag.	Charge [epi/sub]	Ogre Int. Dist.[Å]	DFT Int. Dist.[Å]	Ogre E_{int} [meV Å^{-2}]	DFT E_{int} [meV Å^{-2}]	DFT Ranking
4	A	[0/0]	2.47	2.25	52	37	1
4	B	[0/0]	3.07	3.03	57	46	4
6	A	[+2/0]	2.97	3.06	75	42	2
2	A	[-2/0]	3.24	2.89	115	54	6
6	B	[+2/0]	3.74	3.34	118	56	10
3	A	[-2/0]	3.00	2.74	121	45	3
2	B	[-2/0]	3.80	3.19	126	55	7
5	A	[+2/0]	3.35	3.01	137	55	8
3	B	[-2/0]	3.97	3.34	138	52	5
5	B	[+2/0]	3.64	2.97	139	56	9
7	A	[+4/0]	2.55	2.35	332	79	11
7	B	[+4/0]	3.14	3.22	333	91	13
1	B	[-4/0]	3.91	2.95	347	82	12
1	A	[-4/0]	4.01	3.30	348	91	14

On average, we note that our electrostatic potential tends to overestimate the interfacial distances compared to DFT, with increasing inaccuracy as the interface models become less favorable. This is likely due to the lack of non-ionic contributions to the binding energy and to interface phenomena like charge density redistribution, which can be handled by DFT but not by a classical potential where the charge of ions is defined a-priori.

S8. Lead sulfochloride/$CsPbBr_3$ interfaces

S8.1 On the $Pb_4S_3Cl_2$ crystal structure

We know from HAADF-STEM images of our previous works[5,8] that the two sulfohalides $Pb_4S_3Br_2$ and $Pb_4S_3Cl_2$ share similar crystal structures, and can both match epitaxially the corresponding Cs-Pb halide perovskite. However, the bulk structure of $Pb_4S_3Cl_2$ was never reported in the literature, and the nanocrystals obtained in our previous work were too small to allow for a proper refinement of the structure by X-ray diffraction.[8]

To construct a CIF file for the simulation of interfaces involving $Pb_4S_3Cl_2$, we therefore adapted the published structure of $Pb_4S_3Br_2$ by extracting the lattice parameters directly from the HAADF-STEM images of a $CsPbCl_3/Pb_4S_3Cl_2$ heterostructure, via Fourier analysis of the lattice periodicity. To ensure maximum accuracy, we calibrated the image using the lattice constant of $CsPbCl_3$ as a reference (5.605 Å). This resulted in a pseudo-tetragonal unit cell for $Pb_4S_3Cl_2$, with estimated lattice parameters $a = c = 7.941$ Å and $b = 14.943$ Å. Finally, the coordinates of atoms inside the unit cell were optimized by DFT, performed using the SCAN+rVV10 functional and while keeping the lattice parameters fixed to the estimated values. **Figure S24** summarizes the process.

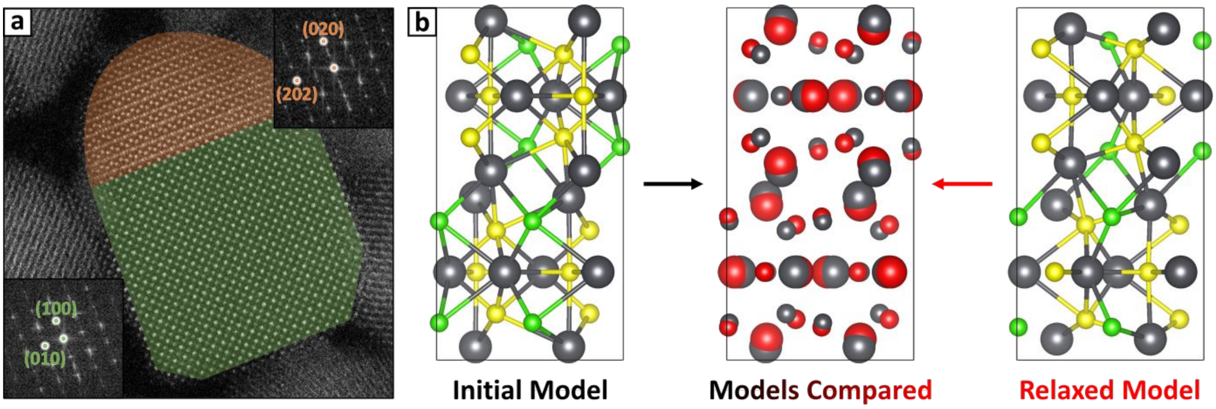

Figure S24. Obtaining a structural model for $Pb_4S_3Cl_2$. a)The unit cell parameters were extracted via the Fourier transform of and HAADF-STEM image of a $Pb_4S_3Cl_2/CsPbCl_3$ heterostructure. The $CsPbCl_3$ domain is highlighted in red, that of in $Pb_4S_3Cl_2$ orange. b) The unit cell was then populated according to the structure reported for $Pb_4S_3Br_2$ (left), followed by a DFT relaxation of the atomic coordinates (center). The process resulted in a mild shift of ions from the starting position (middle). HAADF-STEM image reproduced with permission, Copyright 2022, the Authors.[8]

S8.1 Lead sulfochloride/$CsPbBr_3$ lattice matching

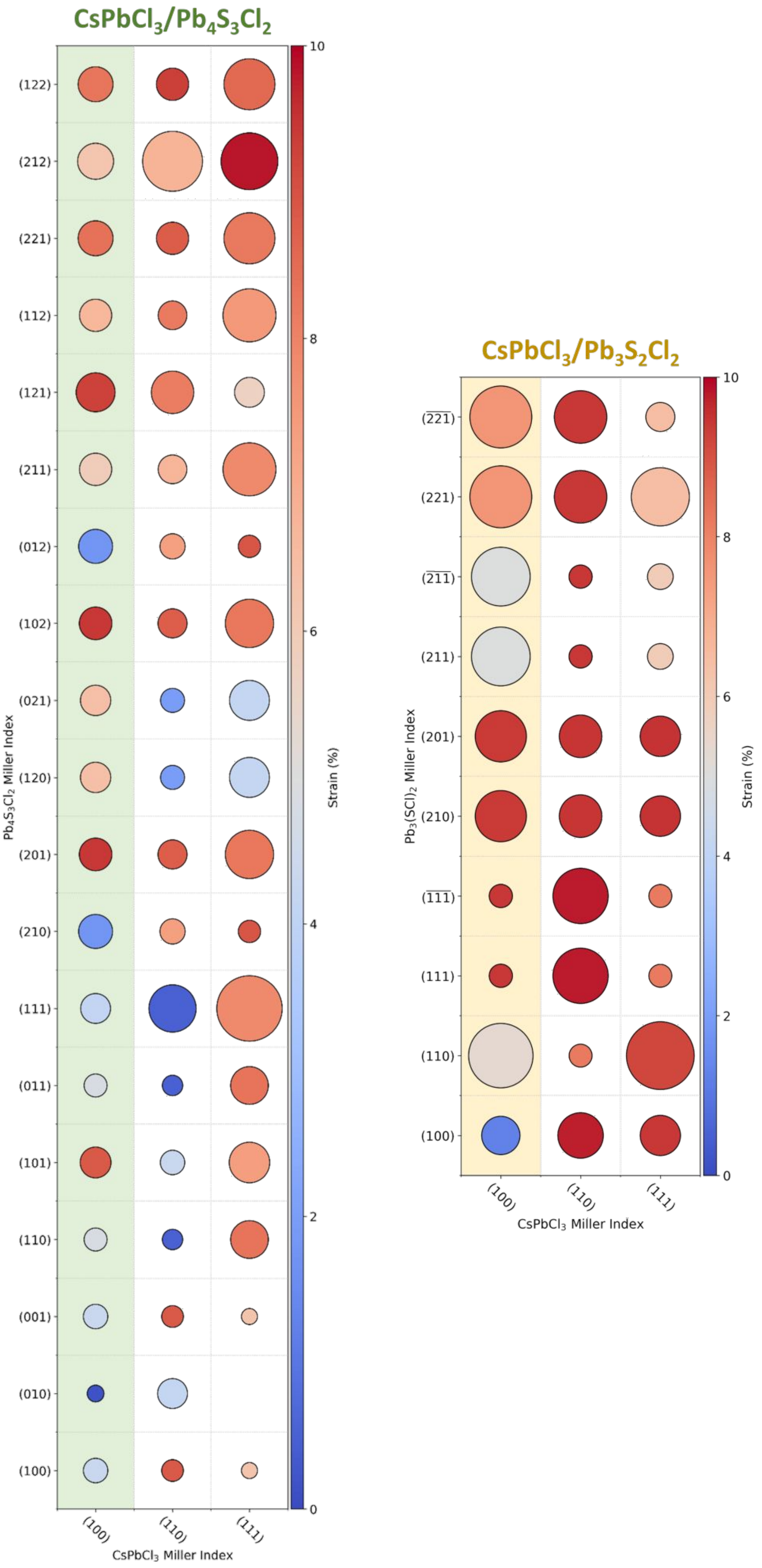

Figure S25. Lattice matching results for $CsPbCl_3/Pb_4S_3Cl_2$ and $CsPbCl_3/Pb_3S_2Cl_2$. The two colored columns contain the interfaces that have been optimized and ranked (see **Table S12**).

Table S12. Summary of all interfaces between (100) $CsPbCl_3$ and lead sulfohalides. Green indicates interfaces with $Pb_4S_3Cl_2$, yellow those with $Pb_3S_2Cl_2$. Darker colors identify the most stable model for both structures. The E_{int} value in parentheses refer to the experimental interface, which is the second most stable model for the (100)(010) – $CsPbCl_3$⫽$Pb_4S_3Cl_2$ interface. Reference structures: CSD-2181723 ($Pb_3S_2Cl_2$, transformed to pseudocubic setting) ; ICSD-201250 ($CsPbCl_3$). For $Pb_4S_3Cl_2$ see **Section S7.1**.

$Pb_xS_yCl_z$ sulfohalide	$Pb_xS_yCl_z$ (hkl)	$Pb_xS_yCl_z$ slab charge	$CsPbCl_3$ slab charge	Strain [%]	Area [Å^2]	Interfacial dist. [Å]	E_{int} [meV Å^{-2}]
$Pb_4S_3Cl_2$	010	0	0	0.2	63	3.08	19 (27)
$Pb_4S_3Cl_2$	011	0	0	4.8	126	2.88	35
$Pb_4S_3Cl_2$	100	0	0	4.3	126	3.09	57
$Pb_4S_3Cl_2$	101	0	0	8.9	188	3.38	60
$Pb_4S_3Cl_2$	201	0	0	9.4	251	2.88	63
$Pb_4S_3Cl_2$	211	-2	0	5.9	251	3.06	64
$Pb_4S_3Cl_2$	212	+1	0	6.2	314	2.97	67
$Pb_4S_3Cl_2$	112	+4	0	6.7	251	2.75	68
$Pb_4S_3Cl_2$	121	-4	0	9.3	377	2.94	71
$Pb_4S_3Cl_2$	111	+2	0	4.1	188	3.07	72
$Pb_4S_3Cl_2$	221	-2	0	8.4	283	3.06	74
$Pb_4S_3Cl_2$	001	0	0	4.3	126	3.51	78
$Pb_4S_3Cl_2$	021	0	0	6.4	188	3.04	80
$Pb_4S_3Cl_2$	122	0	0	8.3	283	3.26	85
$Pb_4S_3Cl_2$	110	+2	0	4.8	126	2.69	86
$Pb_4S_3Cl_2$	012	+2	0	1.8	251	2.96	86
$Pb_4S_3Cl_2$	120	0	0	6.4	188	3.69	92
$Pb_4S_3Cl_2$	102	+2	0	9.4	251	3.57	97
$Pb_4S_3Cl_2$	210	+4	0	1.8	251	3.24	99
$Pb_3S_2Cl_2$	201	-1	0	9.4	314	3.31	50
$Pb_3S_2Cl_2$	-2-1-1	+2	0	5.0	377	3.96	53
$Pb_3S_2Cl_2$	210	-1	0	9.4	314	3.58	61
$Pb_3S_2Cl_2$	100	+1	0	1.3	157	3.29	65
$Pb_3S_2Cl_2$	211	+2	0	5.0	377	3.48	67
$Pb_3S_2Cl_2$	-2-2-1	0	0	7.6	440	3.86	75
$Pb_3S_2Cl_2$	111	0	0	9.5	126	3.28	88
$Pb_3S_2Cl_2$	221	-1	0	7.6	440	4.04	89
$Pb_3S_2Cl_2$	-1-1-1	0	0	9.5	126	3.47	93

S9. $Bi_xPb_yS_z/CsPbBr_3$ interfaces

Considering *h,k,l* ≤ *2*, there are 105 possible non-equivalent epitaxial relations between the (100) surface of $CsPbBr_3$ and the $Bi_xPb_yS_z$ phases considered in this work (19 for heyrovskyite, 19 for lillianite, 29 for xilingolite, 19 for cosalite, and 19 for galenobismuthite). Among them, we identified 10 supercells (**Figure S26**) that fulfilled all criteria discussed in the Main Text. **Table S13** summarizes the outcome of *surface matching and ranking*, while **Figure S26** shows the best model for the three top-ranking interfaces.

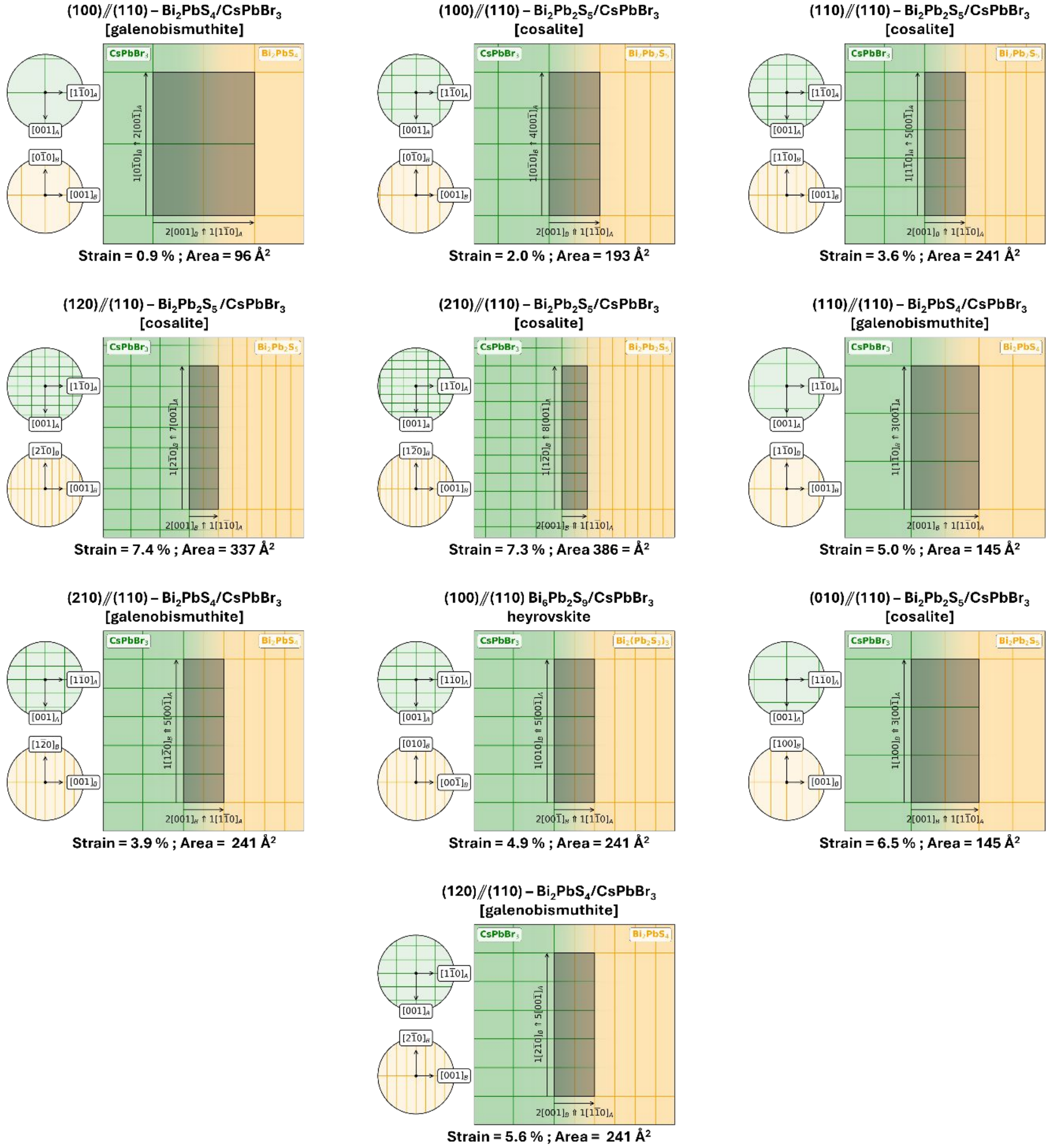

Figure S26. 2D-supercells of the $Bi_xPb_yS_z/CsPbBr_3$ epitaxial relations considered. All supercells share the same relative orientation between the lattices of $CsPbBr_3$ and $Bi_xPb_yS_z$, as shown by the circular dials on the left of each panel. The order of panels follows the stability of the related interfaces (see **Table S13)**.

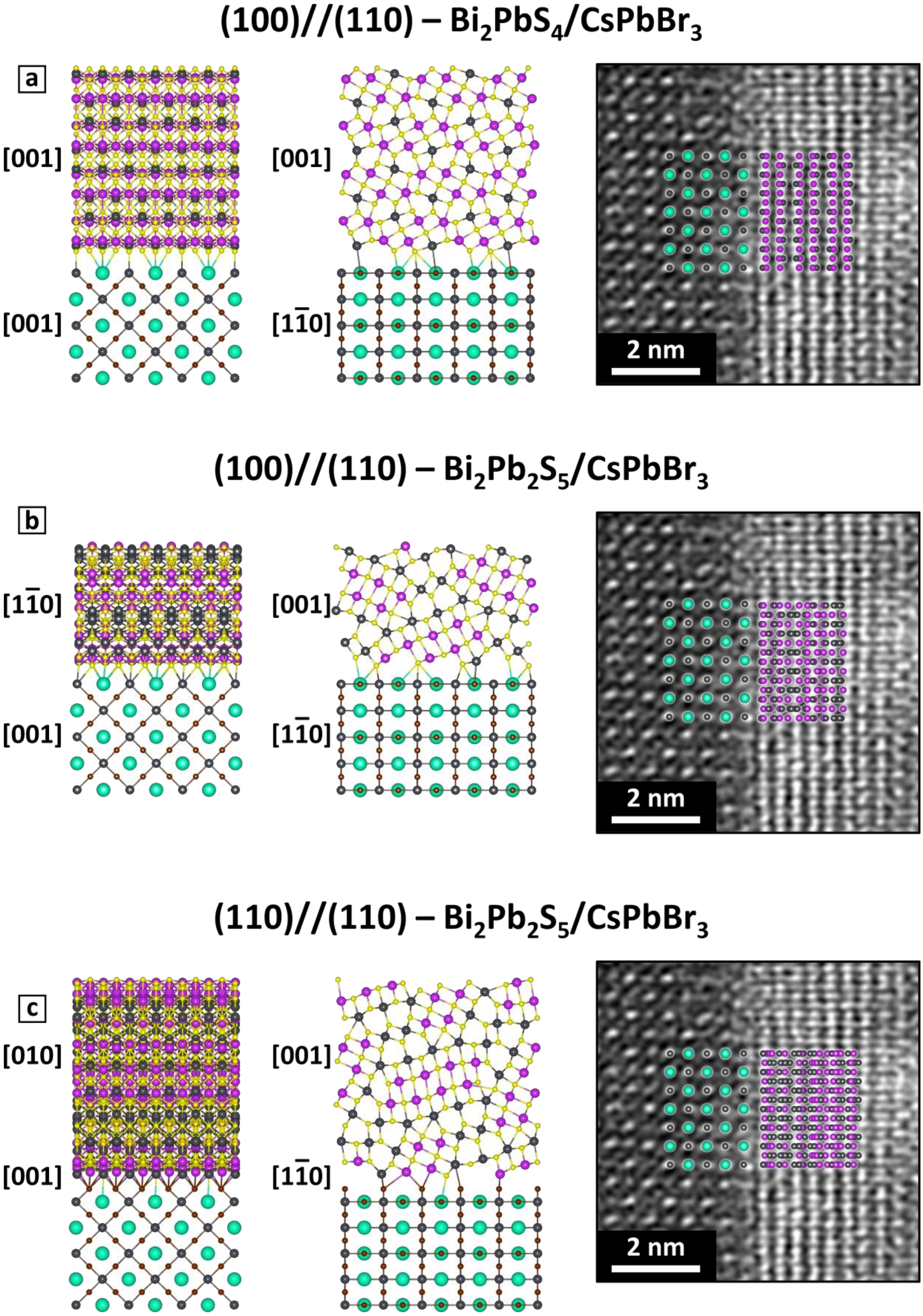

Figure S27. Best $Bi_xPb_yS_z/CsPbBr_3$ interfaces by $\boldsymbol{E_{int}}$. From top to bottom: a) (100)⫽(110) – $Bi_2PbS_4/CsPbBr_3$ galenobismuthite. b) (100)⫽(110) – $Bi_2Pb_2S_5/CsPbBr_3$ cosalite. c) (110)⫽(110) – $Bi_2Pb_2S_5/CsPbBr_3$ cosalite. From left to right: Left) view of the interface along the [001] $CsPbBr_3$ zone axis, as seen in the atomic-resolution TEM images. Center) Side-view of the interface (90° rotation), highlighting the partial formation of bonds between slabs. Right) Interface model overlaid to the atomic-resolution TEM image of a heterostructure. Only the heavy atoms (Pb and Bi) are shown to provide a better comparison with electron scattering contrast. Microscopy images reproduced with permission.[9] Copyright 2023, the Authors.

Table S13. $Bi_xPb_yS_z$/$CsPbBr_3$ interfaces. *Structure optimization* parameters for the interfaces identified between $CsPbBr_3$ and one of the $Bi_xPb_yS_z$ phases, based on the geometric and structural considerations illustrated in **Figure 7b** of the Main Text. The three best models are depicted and overlaid to atomic-resolution images of the heterostructure in **Figure S27**. Reference structures: ICSD-43657 (cosalite); ICSD-604473 (galenobismuthite); ICSD-60160 (heyrovskyite); ICSD-2737 (lillianite); ICSD-92981 (xilingolite).

$Bi_xPb_yS_z$ material	$Bi_xPb_yS_z$ (hkl)	$CsPbBr_3$ (hkl)	BixPbySz slab charge	$CsPbCl_3$ slab charge	Strain [%]	Area [Å^2]	Int. dist. [Å]	E_{int} [meV Å^{-2}]
Galenobismuthite Bi_2PbS_4	100	110	0	+1	0.9	96	3.34	46
Cosalite $Bi_2Pb_2S_5$	100	110	-1	+1	2.0	193	2.87	49
Cosalite $Bi_2Pb_2S_5$	110	110	+3	-1	3.6	241	2.01	51
Cosalite $Bi_2Pb_2S_5$	120	110	+3	-1	7.4	337	2.17	87
Cosalite $Bi_2Pb_2S_5$	210	110	-1	+1	7.3	386	3.11	117
Galenobismuthite Bi_2PbS_4	110	110	+2	-1	5.0	145	2.44	121
Galenobismuthite Bi_2PbS_4	210	110	-2	+1	3.9	241	2.69	131
Heyrovskyite A $Bi_6Pb_2S_9$	100	110	+6	-1	4.9	241	2.01	146
Cosalite $Bi_2Pb_2S_5$	010	110	+3	-1	6.5	145	1.92	151
Galenobismuthite Bi_2PbS_4	120	110	+3	-1	5.6	241	1.75	214
Heyrovskyite B $Bi_6Pb_2S_9$	100	110	-2	+1	4.9	241	3.05	247

Notes: due to standards in the assignment of crystallographic axes, not all the bulk structures for $Bi_xPb_yS_z$ materials have their preferred growth axis labelled as *c*. However, to simplify the discussion we converted all structures into non-standard settings where the growth axis is *c*. We also note that the bulk structure of heyrovskyite features atomic sites with partial occupancy, which is not currently implemented in our software. To circumvent this issue, we constructed two stoichiometric bulk models with no occupational disorder (here denoted heyrovskyite A and heyrovskyite B), and optimized both. Reference CIFs converted in the non-standard space groups and without occupational disorder are provided in the Supplementary Material.

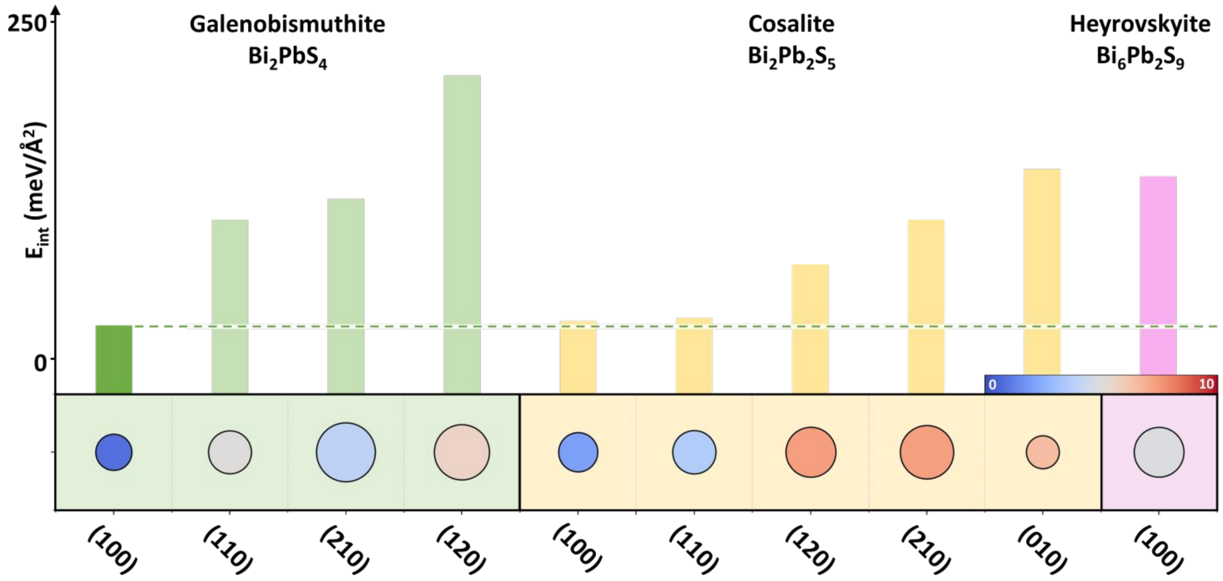

Figure S28. $Bi_xPb_yS_z/CsPbBr_3$ interfaces. *Lattice matching* and interface ranking results for the growth of galenobismuthite (green), cosalite (yellow) and heyrovskyite (purple) on the (110) surface of $CsPbBr_3$, summarized in **Table S13.** The circle size corresponds to the interface area and the color corresponds to the strain. The green dashed line marks the E_{int} value of the experimental interface (100)⫽(110) – $Bi_2PbS_4/CsPbBr_3$.

S10. Other interfaces with $CsPbBr_3$

S10.1. $CsPbBr_3/CsPb_2Br_5$

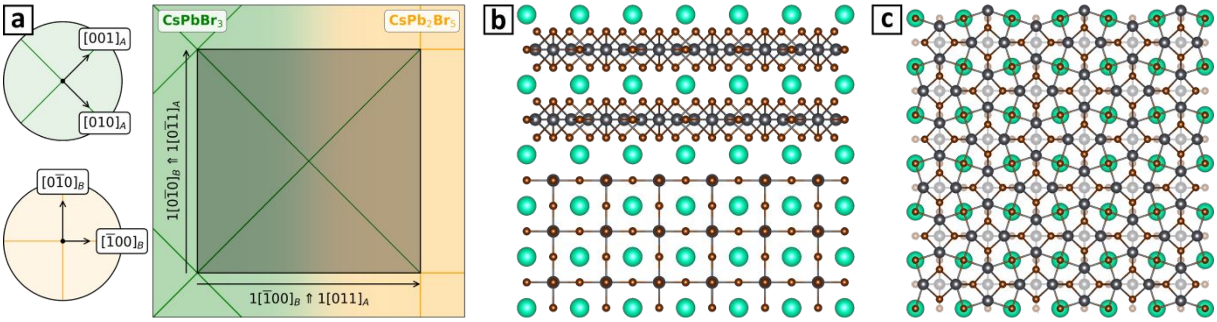

Figure S29. (100)∥(001) – $CsPbBr_3/Pb_4S_3Br_2$ interface. a) 2D-supercell of the interface (strain = 2.7%, area = 68 Å^2). b) Side view of the most stable model as ranked by Ogre (green in **Table S14**). c) Top-down view of the interface layer. The shaded atoms belong to $CsPbBr_3$. Reference structure: ICSD- 254290 ($CsPb_2Br_5$).

Table S14. *Surface ranking* results. Green indicates the most stable interface.

$CsPb_2Br_5$ slab index	$CsPbBr_3$ slab index	Interfacial dist. [Å]	$CsPb_2Br_5$ slab charge	$CsPbBr_3$ slab charge	E_{int} [meV Å^{-2}]
1	0	2.87	+1	0	10
1	1	3.88	+1	0	15
2	1	4.31	-1	0	19
2	0	4.31	-1	0	20
3	0	3.28	+3	0	133
3	1	4.07	+3	0	155
0	1	4.02	-3	0	160
0	0	4.31	-3	0	161

S10.2. $CsPbBr_3$/ZnS

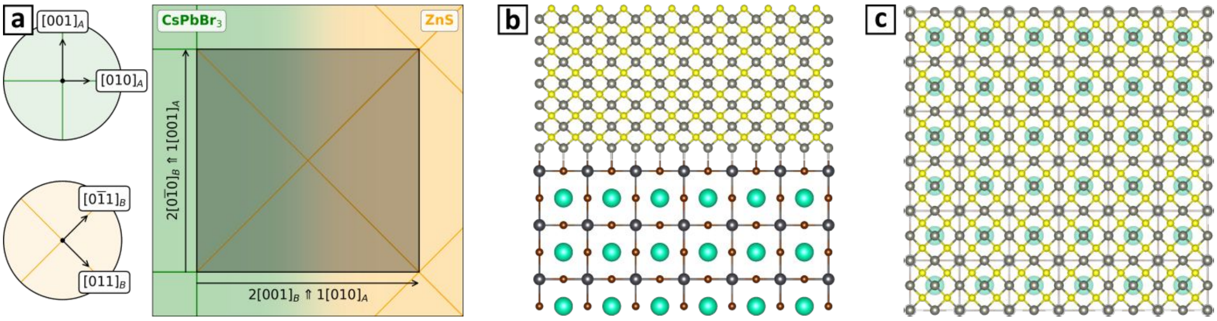

Figure S30. (100)∥(100) – $CsPbBr_3$/ZnS interface. a) 2D-supercell of the interface (strain = 8.0%, area = 34 Å^2). b) Side view of the most stable model as ranked by Ogre (green in **Table S15**). c) Top-down view of the interface layer. The shaded atoms belong to $CsPbBr_3$. Reference structure: ICSD- 254290 (ZnS sphalerite).

Table S15. *Surface ranking* results. Green indicates the most stable interface.

ZnS slab index	$CsPbBr_3$ slab index	Interfacial dist. [Å]	ZnS slab charge	$CsPbBr_3$ slab charge	E_{int} [meV Å^{-2}]
0	0	2.68	1	0	412
1	0	3.89	-1	0	439
0	1	4.22	1	0	441
1	1	4.22	-1	0	444

S10.3. $CsPbBr_3/Al_2O_3$

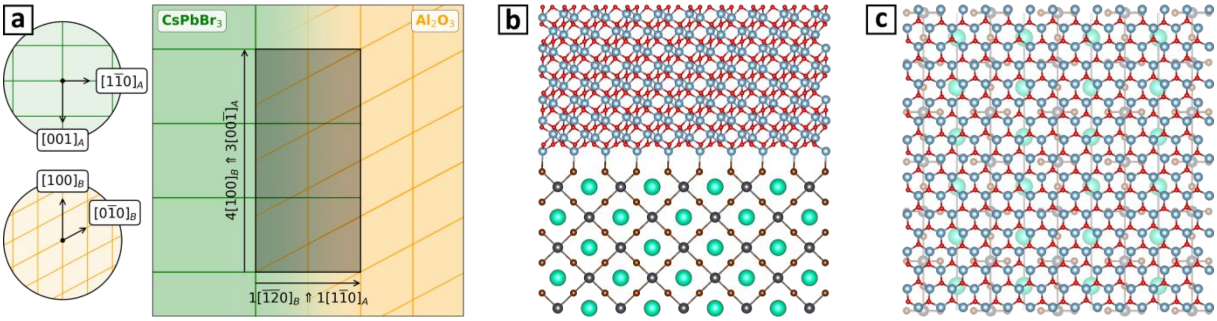

Figure S31. (110)∥(001) – $CsPbBr_3/Al_2O_3$ interface. a) 2D-supercell of the interface (strain = 6.0%, area = 145 Å^2). b) Side view of the most stable model as ranked by Ogre (green in **Table S16**). c) Top-down view of the interface layer. The shaded atoms belong to $CsPbBr_3$. Reference structure: ICSD-111371 (Al_2O_3 sapphire).

Table S16. *Surface ranking* results. Green indicates the most stable interface.

Al_2O_3 slab index	$CsPbBr_3$ slab index	Interfacial dist. [Å]	Al_2O_3 slab charge	$CsPbBr_3$ slab charge	E_{int} [meV Å^{-2}]
0	1	2.69	0	-1	263
12	1	2.69	0	-1	263
0	0	4.22	0	+1	367
12	0	4.22	0	+1	367
2	1	1.81	+3	-1	1807
1	0	3.02	-3	+1	2018

S8.4. $CsPbBr_3/Bi_2WO_6$

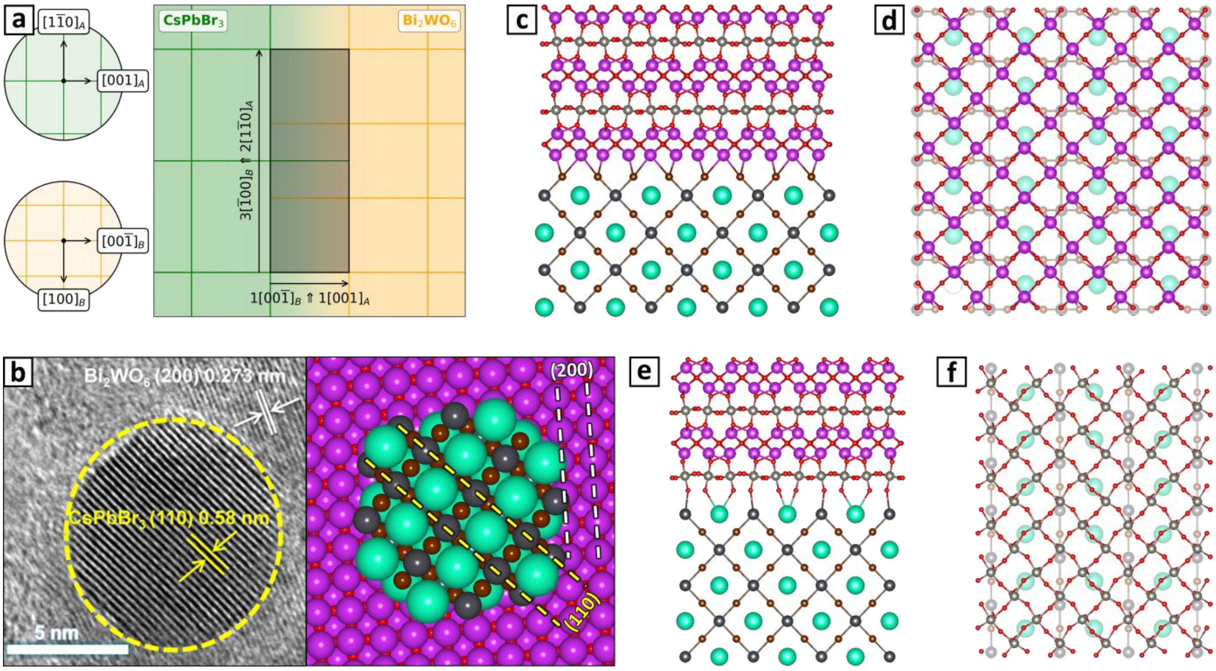

Figure S32. (110)//(010) – $CsPbBr_3/Bi_2WO_6$ interface. a) 2D-supercell of the interface (strain =5.0%, area = 96 Å^2). b) TEM image of a $CsPbBr_3/Bi_2WO_6$ heterostructure side-by-side with the proposed model. $CsPbBr_3$ is indexed in the *Pnma* setting, following the original publication. TEM reproduced with permission.[13] Copyright 2020, American Chemical Society. Side view of the 1st-ranking model (green in **Table S17**). Its structure is similar to the 2nd ranking model, where Bi_2WO_6 is also bismuth-terminated (not shown). d) Top-down view of the same interface. e) Side view of the 3rd-ranking model by Ogre (blue in **Table S17**). d) Top-down view of the interface layer. Shaded atoms belong to $CsPbBr_3$. Reference structure: ICSD- 67647 (Bi_2WO_6).

Table S17. ***Surface ranking*** **results**. Green = **Figure 32c-d** . Blue = **Figure 32b,e-f**.

Bi_2WO_6 slab index	$CsPbBr_3$ slab index	Interfacial dist. [Å]	Bi_2WO_6 slab charge	$CsPbBr_3$ slab charge	E_{int} [meV Å^{-2}]
6	1	2.51	+2	-1	248
14	1	2.47	+2	-1	257
9	0	2.86	-2	+1	293
1	0	2.85	-2	+1	298
10	1	2.71	+2	-1	466
2	1	2.72	+2	-1	471
13	0	2.89	-2	+1	519
5	0	2.92	-2	+1	531
15	0	2.77	-4	+1	1383
7	0	2.71	-4	+1	1395
0	1	2.30	+4	-1	1407
8	1	2.34	+4	-1	1431
3	1	2.30	+6	-1	3546
11	1	2.34	+6	-1	3565
12	0	2.55	-6	+1	3818
4	0	2.55	-6	+1	3842

S11. Validation on oxide interfaces

S11.1. ZnO/Zn_2GeO_4 interface.

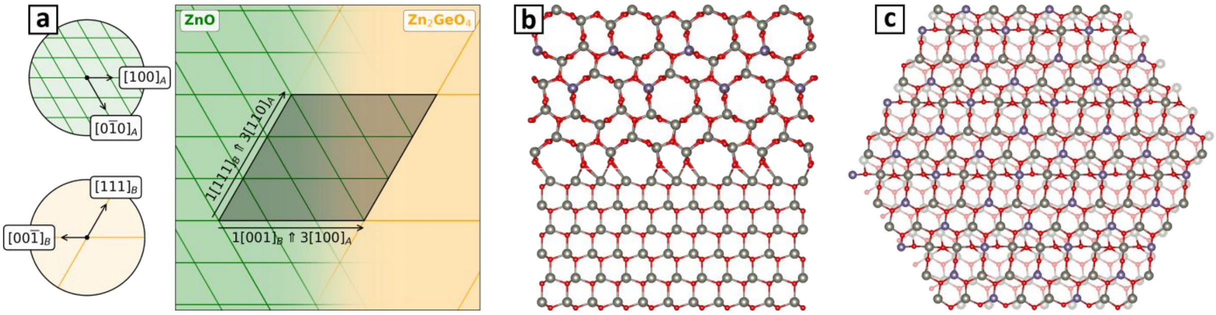

Figure S33. (001)⫽(-110) – ZnO/Zn_2GeO_4 interface. a) 2D-supercell of the interface (strain = 11.9%, area = 82 Å^2). b) Side view of the most stable model as ranked by Ogre (green in **Table S18**). c) Top-down view of the interface layer. Shaded atoms belong to ZnO. Reference structures: ICSD-26170 (**ZnO**) ; ICSD-68382 (**Zn_2GeO_4**).

Table S18. ***Surface ranking*** **results**. Green indicates the most stable interface.

Zn_2GeO_4 slab index	ZnO slab index	Interfacial dist. [Å]	Zn_2GeO_4 slab charge	ZnO slab charge	E_{int} [meV Å^{-2}]
8	0	1.70	-6	0	333
6	1	0.83	10	-2	518
4	0	1.74	0	0	528
1	0	1.69	-2	0	570
5	0	1.96	-8	0	577
3	1	0.73	8	-2	817
2	0	1.70	-10	0	973
0	1	1.41	6	-2	1198
7	1	1.50	2	-2	2087
4	1	2.17	0	-2	2790

S11.2. $LaAlO_3/TiO_2$ (anatase) interface.

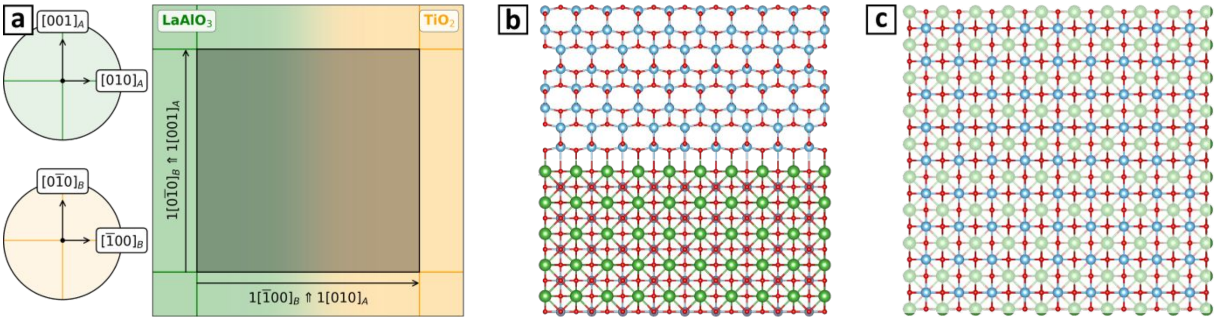

Figure S34. (100)∥(001) – $LaAlO_3/TiO_2$ (anatase) interface. a) 2D-supercell of the interface (strain = 0.2%, area = 14 Å^2). b) Side view of the most stable model as ranked by Ogre (green in **Table S19**). c) Top-down view of the interface layer. Shaded atoms belong to $LaAlO_3$. Reference structures: ICSD-170772 ($LaAlO_3$) ; ICSD-9852 (TiO_2 anatase).

Table S19. ***Surface ranking*** **results**. Green indicates the most stable interface.

TiO_2 slab index	$LaAlO_3$ slab index	Interfacial dist. [Å]	TiO_2 slab charge	$LaAlO_3$ slab charge	E_{int} [meV Å^{-2}]
1	0	2.57	0	+0.5	102
1	1	2.58	0	-0.5	167
2	1	2.09	+2	-0.5	1002
0	0	2.46	-2	+0.5	1369

S11.4. $LaAlO_3$/ZnO interface.

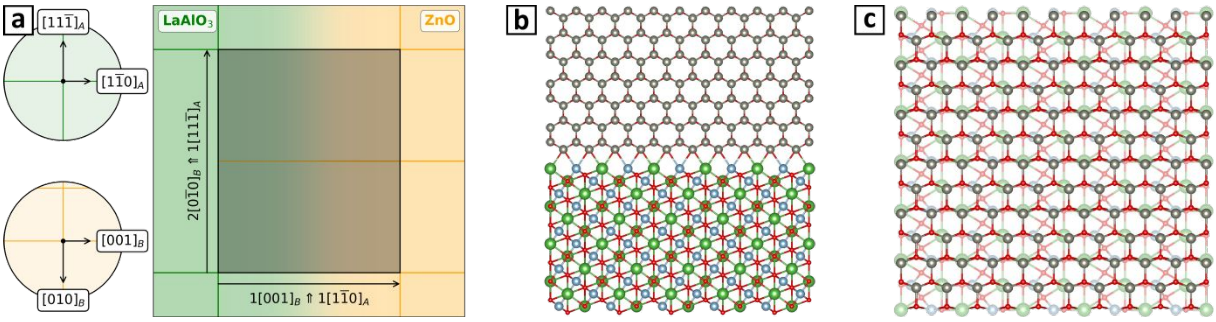

Figure S35. (112)∥(100) – $LaAlO_3$/ZnO interface. a) 2D-supercell of the interface (strain = 2.2%, area = 35 Å^2). b) Side view of the most stable model as ranked by Ogre (green in **Table S20**). c) Top-down view of the interface layer. Shaded atoms belong to $LaAlO_3$. Reference structures: ICSD-170772 ($LaAlO_3$) ; ICSD-26170 (ZnO).

Table S20. ***Geometry optimization*** **results**. Green indicates the most stable interface.

ZnO slab index	$LaAlO_3$ slab index	Interfacial dist. [Å]	ZnO slab charge	$LaAlO_3$ slab charge	E_{int} [meV Å^{-2}]
0	0	2.43	0	2	462
0	1	2.39	0	-2	544

S11.5. $Fe_3O_4/SrTiO_3$ interface.

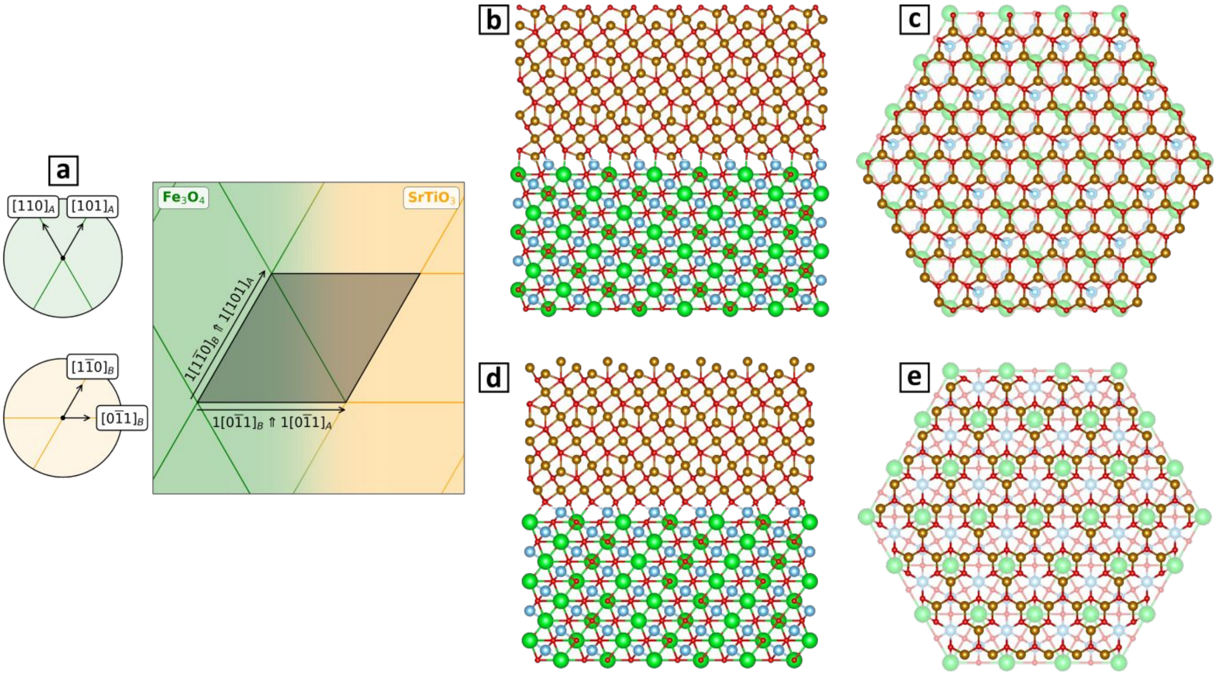

Figure S36. (-111)∥(111) – $Fe_3O_4/SrTiO_3$ interface. a) 2D-supercell of the interface (strain = 7.5%, area = 31 Å^2). b) Side view of the 1st-ranking model (green in **Table S21**). c) Top-down view of the same interface. d) Side view of the 2rd-ranking model by Ogre (blue in **Table S21**). d) Top-down view of the interface layer. Shaded atoms belong to $SrTiO_3$. Reference structures: ICSD- 26410 (Fe_3O_4) ; ICSD- 23076 ($SrTiO_3$).

Table S21. ***Geometry optimization*** **results**. Green indicates the most stable interface.

$SrTiO_3$ slab index	Fe_3O_4 slab index	Interfacial dist. [Å]	$SrTiO_3$ slab charge	Fe_3O_4 slab charge	E_{int} [meV Å^{-2}]
0	5	0.84	+2	-1.3	140
0	4	1.07	+2	-4.0	407
1	0	1.92	-2	+1.3	461
0	2	1.26	+2	-4.0	522
1	1	1.63	-2	+4.0	957
1	3	2.12	-2	+4.0	1080

S12. Introduction to the OgreInterface app

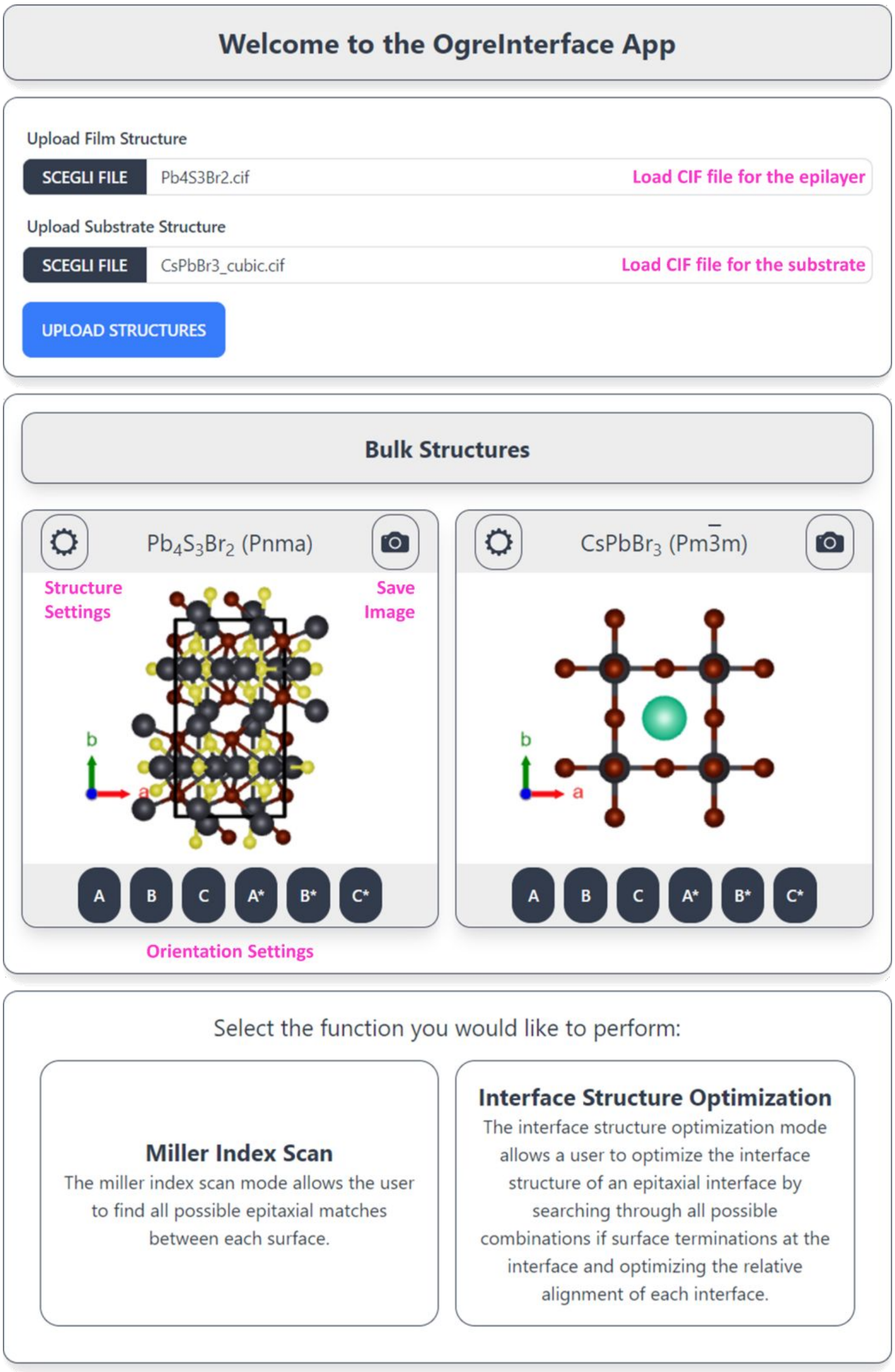

Figure S37. Welcome and structure upload panel of the OgreInterface app. The version shown here is the 0.0.14, and might not reflect the current version available online.

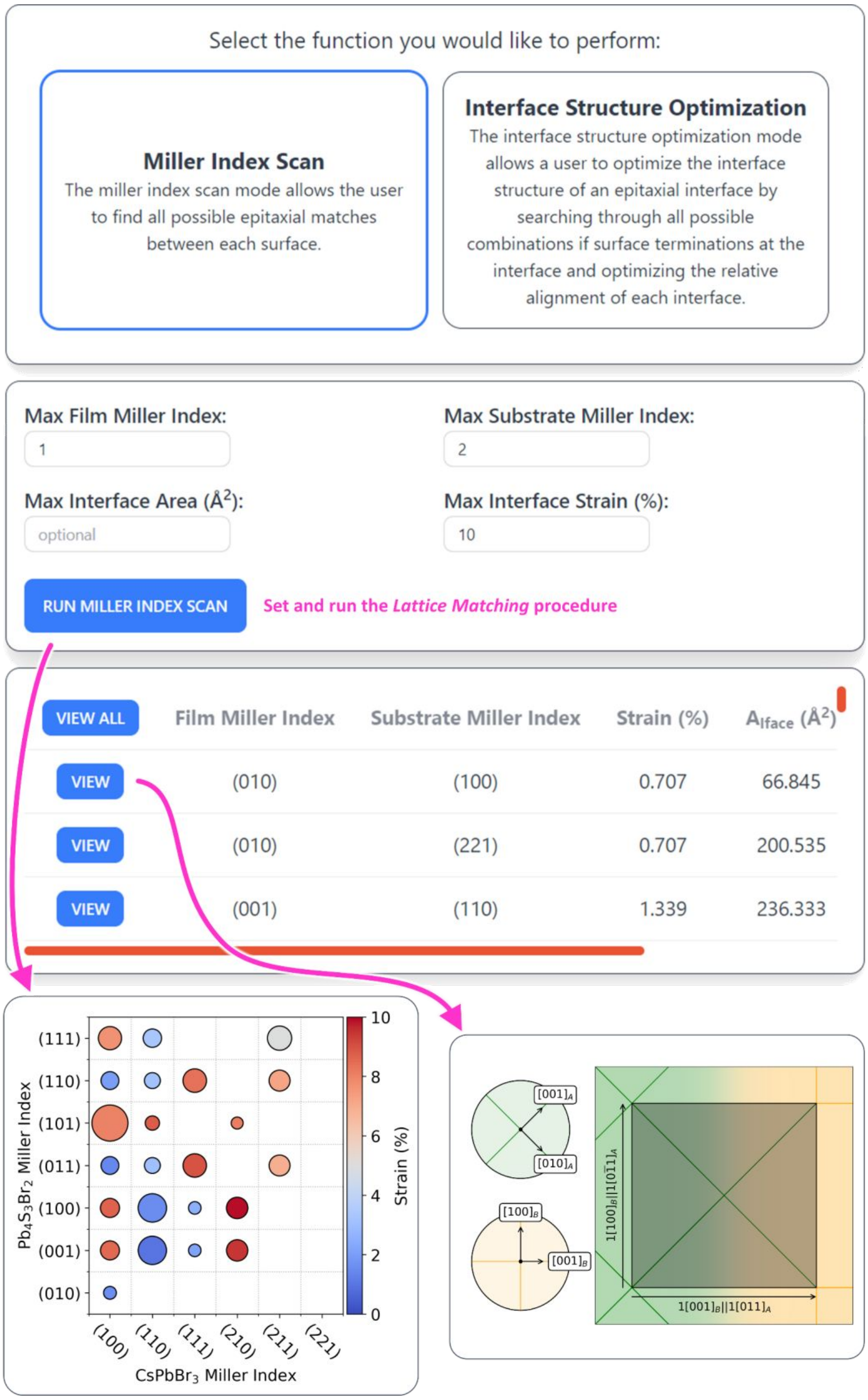

Figure S38. ***Lattice Matching*** **panel of the OgreInterface app.** The purple arrows indicate clickable elements of the panel and their respective outcome when selected.

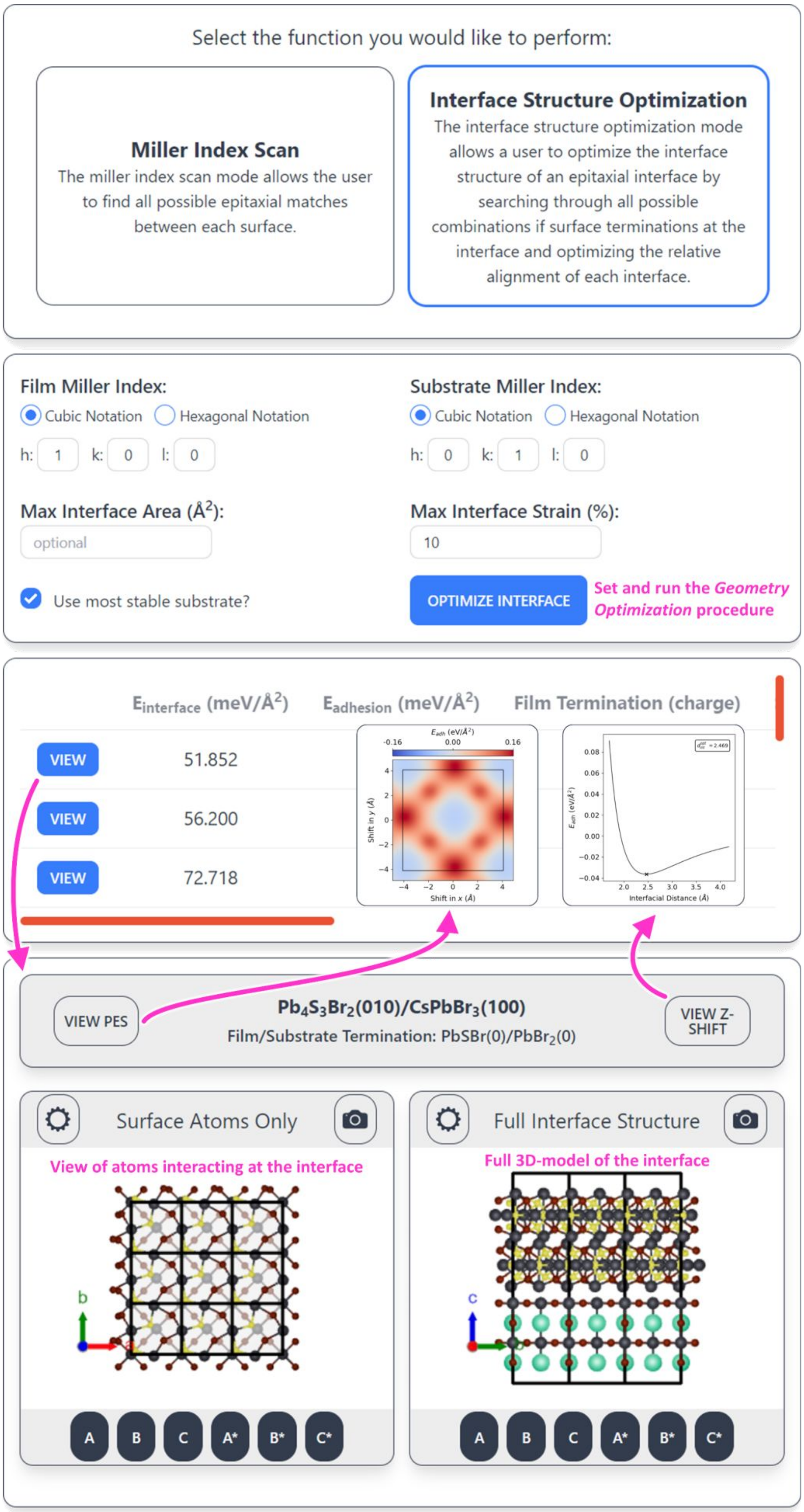

Figure S39. ***Structure Optimization*** **panel of the OgreInterface app.** The purple arrows indicate clickable elements of the panel and their respective outcome when selected.

S13. Ogre performances on a consumer-grade laptop

All simulations based on the Ogre classical potential work were executed on a consumer-grade laptop (ASUS-N552V) equipped with an Intel® Core™ i7-6700HQ CPU @ 2.60GHz processor, 16 GB of RAM and a Samsung SSD 970 EVO Plus hard drive. On this configuration, simulations for the (100)∥(010) – $CsPbBr_3/Pb_4S_3Br_2$ test interface required ~100 s. Factors influencing the duration of calculations are the area of the supercell and the complexity of crystal structures involved, which might significantly increase computation times (up to ~1500 s for the most complex interfaces considered in this work).

The algorithm was executed and is made available in the form of a Jupyter notebook script, in the same form as uploaded to GitHub as a part of the Supplementary Material of this work. An additional script is made available to reproduce all simulations in this work. Note that the execution might require several hours depending on the computer performances.

S14. Supplementary References

(1) Szafrański, M.; Katrusiak, A.; Ståhl, K. Time-Dependent Transformation Routes of Perovskites $CsPbBr_3$ and $CsPbCl_3$ under High Pressure. *J. Mater. Chem. A* **2021**, *9*, 10769–10779.

(2) Rodová, M.; Brožek, J.; Knížek, K.; Nitsch, K. Phase Transitions in Ternary Caesium Lead Bromide. *J. Therm. Anal. Calorim.* **2003**, *71*, 667–673.

(3) Straus, D. B.; Guo, S.; Abeykoon, A. M.; Cava, R. J. Understanding the Instability of the Halide Perovskite $CsPbI_3$ through Temperature-Dependent Structural Analysis. *Adv. Mater.* **2020**, *32*, 2001069.

(4) Bertolotti, F.; Protesescu, L.; Kovalenko, M. V.; Yakunin, S.; Cervellino, A.; Billinge, S. J. L.; Terban, M. W.; Pedersen, J. S.; Masciocchi, N.; Guagliardi, A. Coherent Nanotwins and Dynamic Disorder in Cesium Lead Halide Perovskite Nanocrystals. *ACS Nano* **2017**, *11*, 3819–3831.

(5) Imran, M.; Peng, L.; Pianetti, A.; Pinchetti, V.; Ramade, J.; Zito, J.; Di Stasio, F.; Buha, J.; Toso, S.; Song, J.; Infante, I.; Bals, S.; Brovelli, S.; Manna, L. Halide Perovskite-Lead Chalcohalide Nanocrystal Heterostructures. *J. Am. Chem. Soc.* **2021**, *143*, 1435–1446.

(6) Pradhan, N. Why Do Perovskite Nanocrystals Form Nanocubes and How Can Their Facets Be Tuned? A Perspective from Synthetic Prospects. *ACS Energy Lett.* **2021**, *6*, 92–99.

(7) Bera, S.; Behera, R. K.; Pradhan, N. α-Halo Ketone for Polyhedral Perovskite Nanocrystals: Evolutions, Shape Conversions, Ligand Chemistry, and Self-Assembly. *J. Am. Chem. Soc.* **2020**, *142*, 20865–20874.

(8) Toso, S.; Imran, M.; Mugnaioli, E.; Moliterni, A.; Caliandro, R.; Schrenker, N. J.; Pianetti, A.; Zito, J.; Zaccaria, F.; Wu, Y.; Gemmi, M.; Giannini, C.; Brovelli, S.;

Infante, I.; Bals, S.; Manna, L. Halide Perovskites as Disposable Epitaxial Templates for the Phase-Selective Synthesis of Lead Sulfochloride Nanocrystals. *Nat. Commun. 2022 131* **2022**, *13*, 1–10.

(9) Rusch, P.; Toso, S.; Ivanov, Y. P.; Marras, S.; Divitini, G.; Manna, L. Nanocrystal Heterostructures Based On Halide Perovskites and Lead-Bismuth Chalcogenides. *Chem. Mater.* **2023**, *18*, 40.

(10) Zheng, Y.; Yang, T.; Fang, Z.; Shang, M.; Zhang, Z.; Yang, J.; Fan, J.; Yang, W.; Hou, X.; Wu, T. All-Inorganic Dual-Phase Halide Perovskite Nanorings. *Nano Res.* **2020**, *13*, 2994–3000.

(11) Ravi, V. K.; Saikia, S.; Yadav, S.; Nawale, V. V.; Nag, A. $CsPbBr_3$/ZnS Core/Shell Type Nanocrystals for Enhancing Luminescence Lifetime and Water Stability. *ACS Energy Lett.* **2020**, *5*, 1794–1796.

(12) Oksenberg, E.; Merdasa, A.; Houben, L.; Kaplan-Ashiri, I.; Rothman, A.; Scheblykin, I. G.; Unger, E. L.; Joselevich, E. Large Lattice Distortions and Size-Dependent Bandgap Modulation in Epitaxial Halide Perovskite Nanowires. *Nat. Commun.* **2020**, *11*, 1–11.

(13) Wang, J.; Wang, J.; Li, N.; Du, X.; Ma, J.; He, C.; Li, Z. Direct Z-Scheme 0D/2D Heterojunction of $CsPbBr_3$ Quantum Dots/Bi_2WO_6 Nanosheets for Efficient Photocatalytic CO_2 Reduction. *ACS Appl. Mater. Interfaces* **2020**, *12*, 31477–31485.

(14) Granerød, C. S.; Aarseth, B. L.; Nguyen, P. D.; Bazioti, C.; Azarov, A.; Svensson, B. G.; Vines, L.; Prytz, Ø. Structural and Optical Properties of Individual Zn_2GeO_4 Particles Embedded in ZnO. *Nanotechnology* **2019**, *30*, 225702.

(15) Islam, M.; Rajak, P.; Knez, D.; Chaluvadi, S. K.; Orgiani, P.; Rossi, G.; Dražić, G.; Ciancio, R. HAADF STEM and Ab Initio Calculations Investigation of Anatase TiO_2/$LaAlO_3$ Heterointerface. *Appl. Sci.* **2022**, *12*, 1489.

(16) Tian, J. S.; Wu, Y. H.; Wang, W. L.; Yen, T. C.; Ho, Y. T.; Chang, L. The Role of the Interface Structure on the Growth of Nonpolar (10-1 0) and Semipolar (11-2 -2) ZnO on (112) $LaAlO_3$ Substrates. *Mater. Lett.* **2013**, *109*, 237–239.

(17) Gilks, D.; McKenna, K. P.; Nedelkoski, Z.; Kuerbanjiang, B.; Matsuzaki, K.; Susaki, T.; Lari, L.; Kepaptsoglou, D.; Ramasse, Q.; Tear, S.; Lazarov, V. K. Polar Spinel-Perovskite Interfaces: An Atomistic Study of Fe_3O_4 (111)/$SrTiO_3$ (111) Structure and Functionality. *Sci. Rep.* **2016**, *6*, 1–7.

(18) Talapin, D. V.; Mekis, I.; Götzinger, S.; Kornowski, A.; Benson, O.; Weller, H. CdSe/CdS/ZnS and CdSe/ZnSe/ZnS Core-Shell-Shell Nanocrystals. *J. Phys. Chem. B* **2004**, *108*, 18826–18831.

(19) Zur, A.; McGill, T. C. Lattice Match: An Application to Heteroepitaxy. *J. Appl. Phys.* **1984**, *55*, 378–386.

(20) Cao, Y. W.; Banin, U. Growth and Properties of Semiconductor Core/Shell Nanocrystals with InAs Cores. *J. Am. Chem. Soc.* **2000**, *122*, 9692–9702.

(21) Das, R.; Patra, A.; Dutta, S. K.; Shyamal, S.; Pradhan, N. Facets-Directed Epitaxially Grown Lead Halide Perovskite-Sulfobromide Nanocrystal Heterostructures and Their Improved Photocatalytic Activity. *J. Am. Chem. Soc.* **2022**, *144*, 18629–18641.

(22) Chu, W.; Saidi, W. A.; Zhao, J.; Prezhdo, O. V. Soft Lattice and Defect Covalency Rationalize Tolerance of β-$CsPbI_3$ Perovskite Solar Cells to Native Defects. *Angew. Chemie - Int. Ed.* **2020**, *59*, 6435–6441.

(23) Lai, M.; Obliger, A.; Lu, D.; Kley, C. S.; Bischak, C. G.; Kong, Q.; Lei, T.; Dou, L.; Ginsberg, N. S.; Limmer, D. T.; Yang, P. Intrinsic Anion Diffusivity in Lead Halide Perovskites Is Facilitated by a Soft Lattice. *Proc. Natl. Acad. Sci. U. S. A.* **2018**, *115*, 11929–11934.

(24) Togo, A.; Tanaka, I. Spglib: A Software Library for Crystal Symmetry Search. **2018**.

(25) Ong, S. P.; Richards, W. D.; Jain, A.; Hautier, G.; Kocher, M.; Cholia, S.; Gunter, D.; Chevrier, V. L.; Persson, K. A.; Ceder, G. Python Materials Genomics (Pymatgen): A Robust, Open-Source Python Library for Materials Analysis. *Comput. Mater. Sci.* **2013**, *68*, 314–319.

(26) Slowik, A. Particle Swarm Optimization. *Ind. Electron. Handb. - Five Vol. Set* **2011**, *4*, 1942–1948.

(27) Guofei9987. Scikit-Opt: Genetic Algorithm, Particle Swarm Optimization, Simulated Annealing, Ant Colony Optimization Algorithm,Immune Algorithm, Artificial Fish Swarm Algorithm, Differential Evolution and TSP(Traveling Salesman).

(28) Schütt, K. T.; Kessel, P.; Gastegger, M.; Nicoli, K. A.; Tkatchenko, A.; Müller, K. R. SchNetPack: A Deep Learning Toolbox for Atomistic Systems. *J. Chem. Theory Comput.* **2019**, *15*, 448–455.

(29) Schütt, K. T.; Hessmann, S. S. P. P.; Gebauer, N. W. A. A.; Lederer, J.; Gastegger, M. SchNetPack 2.0: A Neural Network Toolbox for Atomistic Machine Learning. *J. Chem. Phys.* **2023**, *158*, 144801.

(30) Himanen, L.; Jäger, M. O. J.; Morooka, E. V.; Federici Canova, F.; Ranawat, Y. S.; Gao, D. Z.; Rinke, P.; Foster, A. S. DScribe: Library of Descriptors for Machine Learning in Materials Science. *Comput. Phys. Commun.* **2020**, *247*, 106949.

(31) Laakso, J.; Himanen, L.; Homm, H.; Morooka, E. V.; Jäger, M. O. J.; Todorović, M.; Rinke, P. Updates to the DScribe Library: New Descriptors and Derivatives. *J. Chem. Phys.* **2023**, *158*, 234802.

(32) Yeh, I. C.; Berkowitz, M. L. Ewald Summation for Systems with Slab Geometry. *J. Chem. Phys.* **1999**, *111*, 3155–3162.

(33) Fennell, C. J.; Gezelter, J. D. Is the Ewald Summation Still Necessary? Pairwise Alternatives to the Accepted Standard for Long-Range Electrostatics. *J. Chem. Phys.* **2006**, *124*, 234104.

(34) Pan, H.; Ganose, A. M.; Horton, M.; Aykol, M.; Persson, K. A.; Zimmermann, N. E. R.; Jain, A. Benchmarking Coordination Number Prediction Algorithms on Inorganic Crystal Structures. *Inorg. Chem.* **2021**, *60*, 1590–1603.

(35) Shannon, R. D. Revised Effective Ionic Radii and Systematic Studies of Interatomic Distances in Halides and Chalcogenides. *Acta Crystallogr. Sect. A* **1976**, *32*, 751–767.

(36) Baloch, A. A. B.; Alqahtani, S. M.; Mumtaz, F.; Muqaibel, A. H.; Rashkeev, S. N.; Alharbi, F. H. Extending Shannon's Ionic Radii Database Using Machine Learning. *Phys. Rev. Mater.* **2021**, *5*, 043804.

(37) Wiktor, J.; Fransson, E.; Kubicki, D.; Erhart, P. Quantifying Dynamic Tilting in Halide Perovskites: Chemical Trends and Local Correlations. *Chem. Mater.* **2023**, *35*, 6737–6744.

(38) Fransson, E.; Wiktor, J.; Erhart, P. Phase Transitions in Inorganic Halide Perovskites from Machine-Learned Potentials. *J. Phys. Chem. C* **2023**, *127*, 13773–13781.
